# Projecting shifts in thermal habitat for 686 species on the North American continental shelf

**DOI:** 10.1371/journal.pone.0196127

**Published:** 2018-05-16

**Authors:** James W. Morley, Rebecca L. Selden, Robert J. Latour, Thomas L. Frölicher, Richard J. Seagraves, Malin L. Pinsky

**Affiliations:** 1 Department of Ecology, Evolution and Natural Resources, Rutgers University, New Brunswick, New Jersey, United States of America; 2 Virginia Institute of Marine Science, College of William & Mary, Gloucester Point, Virginia, United States of America; 3 Climate and Environmental Physics, Physics Institute, University of Bern, Bern, Switzerland; 4 Oeschger Centre for Climate Change Research, University of Bern, Bern, Switzerland; 5 Mid-Atlantic Fishery Management Council, Dover, Delaware, United States of America; Technical University of Denmark, DENMARK

## Abstract

Recent shifts in the geographic distribution of marine species have been linked to shifts in preferred thermal habitats. These shifts in distribution have already posed challenges for living marine resource management, and there is a strong need for projections of how species might be impacted by future changes in ocean temperatures during the 21^st^ century. We modeled thermal habitat for 686 marine species in the Atlantic and Pacific oceans using long-term ecological survey data from the North American continental shelves. These habitat models were coupled to output from sixteen general circulation models that were run under high (RCP 8.5) and low (RCP 2.6) future greenhouse gas emission scenarios over the 21^st^ century to produce 32 possible future outcomes for each species. The models generally agreed on the magnitude and direction of future shifts for some species (448 or 429 under RCP 8.5 and RCP 2.6, respectively), but strongly disagreed for other species (116 or 120 respectively). This allowed us to identify species with more or less robust predictions. Future shifts in species distributions were generally poleward and followed the coastline, but also varied among regions and species. Species from the U.S. and Canadian west coast including the Gulf of Alaska had the highest projected magnitude shifts in distribution, and many species shifted more than 1000 km under the high greenhouse gas emissions scenario. Following a strong mitigation scenario consistent with the Paris Agreement would likely produce substantially smaller shifts and less disruption to marine management efforts. Our projections offer an important tool for identifying species, fisheries, and management efforts that are particularly vulnerable to climate change impacts.

## Introduction

A major impact of climate change in the oceans has been the redistribution of marine organisms, which have generally been shifting poleward or into deeper waters as temperatures warm [1, 2]. Long-term shifts in species distributions have been linked to directional shifts in their preferred temperatures [3, 4], as the geographic distributions of marine species are strongly linked to temperature tolerance [1]. Further, regional species productivity [5, 6] and phenology [7, 8] can be highly sensitive to variation in water temperatures, which may be a driver of long-term shifts [9]. The implications of geographic shifts of marine species have already been observed in global fisheries catches and changes in catch composition from regional landings data are consistent with poleward shifts in species distributions [10].

The North American continental shelf is an expansive area with some of the most productive fisheries globally [11]. This diverse area also contains some of the most rapidly increasing regions of ocean temperature in the world [12, 13]. The rising temperatures have been linked to major shifts in the distribution of some species [3, 14]. These shifts have led to conflicts between regions over fisheries catch allocation as species shift across management boundaries [15, 16]. Global ocean temperatures are projected to continue rising [17] and areas of the Northeast American shelf may experience some of the most extreme increases [18]. Associated with this warming are predictions for substantial shifts in regional fisheries productivity [11]. Predictions for how ocean warming will impact the living marine resources of the United States and Canada are currently a priority for federal management [19–21].

Projections of future species distribution shifts and biomass changes are an emerging tool for anticipating climate change impacts on marine systems. Predictions about how species will respond to ocean warming are often made by coupling models of species thermal habitat with output from climate projection models [22]. Generally, species projection studies indicate that biomass will shift with preferred thermal habitat [23–25], but shifts may be constrained by other habitat features such as depth [26], seafloor complexity [27], primary productivity [28], salinity [29], or ocean carbonate chemistry [30]. Further, for some species, annual temperature extremes instead of thermal averages can be a primary driver of projected shifts in distribution [31, 32] or of regional biomass [33].

Previous studies that projected species distribution changes have been limited for two major reasons. First, the spatial extents for species distribution projections are often restricted to regional scales [24, 26, 27]. While these regional perspectives are valuable, they limit our ability to anticipate the larger scale changes that will occur over the entire range of species. Further, species habitat models are often based on data from only a portion of the total geographic range, and thus may fail to capture the full extent of a species’ realized thermal niche. For instance, climate change may introduce novel conditions to a region, such as higher annual maximum temperatures, which may be unrepresented in habitat models built from narrow-scale data. In contrast, some notable species projection efforts have been global in scope [25, 34], but these have often been at a coarse spatial grain that makes it more challenging to define accurate thermal envelopes for marine species. The coarse spatial grain of these latter studies also makes them less directly useful at the regional scales that are particularly relevant for questions of community turnover and resource management. A second limitation of many previous studies has been a relative lack of uncertainty estimates for predictions among species [35, 36]. Uncertainty in projections of species distribution comes from multiple sources, including uncertainty over future greenhouse gas emissions scenarios, variation among climate projection model predictions, natural internal variability inherent in the climate system, structural differences among species distribution models, and parameter uncertainty in species distribution models. The first three factors impact predictions of regional ocean temperature changes [37] and can lead to a wide range of predicted outcomes for individual species [31, 33, 38]. There have been relatively few marine species distribution projections to date, and it remains unclear how much uncertainty is acceptable for a projection to be useful. Conducting projections on multiple species within a standard format for assessing uncertainty can provide opportunities for identifying species with more or less robust predictions [23, 28].

Here we summarize results from a comprehensive effort to project future distributions of 686 marine species on the North American continental shelf. For each species, we generated predictions of their distribution throughout the 21^st^ century using sixteen fully coupled general circulation models, each run under a low (Representative Concentration Pathway (RCP) 2.6) and high (RCP8.5) future greenhouse gas emission scenario. Thus, thirty-two projections were simulated for each species. We used three metrics for identifying species with more uncertain or more robust projections based on agreement among modeled outcomes. We have expanded on previous efforts to predict the responses of marine species to climate change by combining extensive survey data from around the continent in order to better define each species’ thermal habitat. Finally, we projected distribution shifts for each species across the North American shelf in order to include a large portion of species’ distributions, rather than limiting the predictions to individual oceanic basins. Compared to previous studies, our projections suggest some of the largest future shifts in species distribution, many exceeding 1000 km. We found that the geometry of the continental shelf played an important role in determining whether species were projected to shift more or less over the 21^st^ century.

## Methods

### Survey data

**Fig 1:**
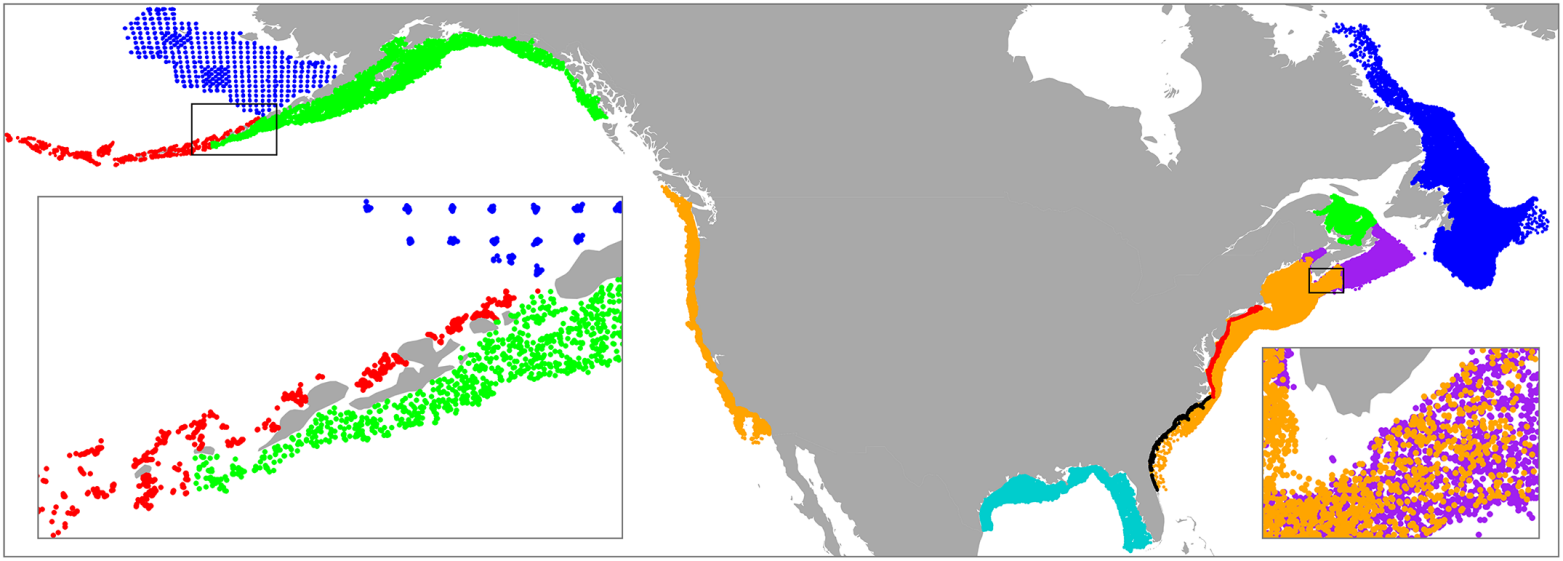
Biological survey regions. Locations for 136,044 bottom trawl hauls from long-term ecological surveys conducted on the North American continental shelf. Hauls are colored to indicate the different survey regions described in Table 1.

Species occurrence and biomass data were taken from 136,044 bottom trawl hauls from twenty long-term surveys that encompassed a majority of the continental shelf regions around the United States and Canada (Fig 1). Within six of the sampling regions, separate surveys were conducted in multiple seasons (Table 1). Most of the survey data have been used in previous studies [3, 9, 39, 40]. A majority of the data from the United States were obtained from the *trawlData* package [39] used with R software version 3.3.2 [41].

**Table 1:** Information for North American bottom trawl surveys.

Region	Seasons	Years	# hauls	Color	Agency
Aleutian Islands	Summer	1983–2014	4588	Red	Alaska Fisheries Science Center, NOAA
Eastern Bering Sea	Summer	1982–2014	12210	Blue	Alaska Fisheries Science Center, NOAA
Gulf of Alaska	Summer	1984–2013	9578	Green	Alaska Fisheries Science Center, NOAA
West Coast Triennial	Summer	1977–2004	4544	Orange	Alaska Fisheries Science Center, NOAA
West Coast Annual	Summer	2003–2014	7589	Orange	Northwest Fisheries Science Center, NOAA
Gulf of Mexico (SEAMAP)	Summer	1982–2014	9277	Cyan	Gulf States Marine Fisheries Commission
	Fall	1983–2014	9564		
Southeast U.S. Shelf (SEAMAP-SA)	Spring	1990–2015	2614	Black	South Carolina Department of Natural Resources
	Summer	1989–2015	2384		
	Fall	1989–2015	2595		
Northeast U.S. Shelf	Spring	1968–2015	16472	Orange	Northeast Fisheries Science Center, NOAA
	Fall	1963–2014	17767		
Northeast U.S. Inner Shelf (NEAMAP)	Spring	2008–2015	1171	Red	Virginia Institute of Marine Science (VIMS)
	Fall	2007–2015	1307		
Scotian Shelf	Spring	1979–2011	5217	Purple	Department of Fisheries and Oceans (DFO), Canada
	Summer	1970–2011	7517		
	Fall	1978–1986	1114		
Southern Gulf of St. Lawrence	Summer	1971–2009	5142	Green	Department of Fisheries and Oceans (DFO), Canada
Newfoundland and Labrador	Spring	1996–2011	4540	Blue	Department of Fisheries and Oceans (DFO), Canada
	Fall	1995–2011	10854		

The season of biological sampling, years of survey data used, number of hauls, color indicator for reference to Fig 1, and conducting agency for twenty regional bottom trawl surveys that were used for fitting species thermal habitat models.

Within each survey, non-standard hauls were omitted (e.g., those with non-standard trawl dimensions or mesh size) in order to ensure consistent sampling gear within surveys. Trawl catch by species was expressed as biomass per unit area swept. Only species-level taxa were considered for distribution modeling. Species names were standardized across surveys. When a species was recorded separately by sex or maturity stage, these biomasses were summed before further analysis. Egg and larval stage samples were excluded from analyses. Because surveys comprehensively record the species found, survey records were expanded to include zeros (observed absences) for species not observed in a given haul, but that were observed in other surveys conducted on the same coast (i.e., Atlantic or Pacific). In addition, we added near-zero biomass values (1.0^−10^) for a fraction of the hauls in regions where a species was never observed (10% of the hauls in a region or 10% of total observations, whichever was smaller) in order to facilitate fitting the full thermal envelope for species biomass models only (i.e., this does not affect presence-absence models, see below). These near-zero values were important to allow biomass models to include a larger range of environmental conditions—as opposed to being restricted to a species present range—which was necessary to conduct biomass predictions at the scale of the North American continental shelf. This approach is similar to the use of “pseudoabsences” in distribution models that are based on presence-only data [42], which are used to develop contrasts between suitable and unsuitable habitats.

### Environmental data

Sea surface and bottom water temperatures for each haul were obtained from the Simple Ocean Data Assimilation (SODA3.3.1) reanalysis of ocean climate, which provides a global reconstruction of historical ocean temperatures [43]. SODA3.3.1 has a spatial resolution of 28 km at the equator with increasingly finer resolution towards the poles (0.25° latitude × 0.25° longitude), and it has a 5-day temporal resolution beginning in 1980. For hauls occurring before 1980, we used the SODA2.2.4 data, which has a 0.5° spatial resolution and monthly temporal resolution. For each haul, we recorded the seasonal average temperature by calculating the average surface and bottom temperatures for a three-month period (including the month when the haul took place and the preceding and following months) and by using the appropriate SODA grid cell based on haul location. This time-interval was chosen to match our projections, which were based on seasonal average temperatures in future years. To check that the SODA-derived seasonal surface and bottom temperatures were reasonable, we compared them to the surface and bottom water temperatures measured at each haul (i.e., not seasonal means) during the course of the surveys. The SODA-derived seasonal average temperatures that we used in the species habitat models were highly correlated with *in situ* measured temperatures (surface temperatures: slope(se) = 0.906(0.001), *P* < 0.001, *r*^*2*^ = 0.90, *DF* = 102048; bottom temperatures: slope(se) = 1.003(0.001), *P* < 0.001, *r*^*2*^ = 0.86, *DF* = 120859).

Marine species temperature preferences are often modeled by only using observed temperatures that are recorded at the time of sampling [3, 24, 27]. However, annual temperature extremes including the summer maximum and winter minimum are also important factors shaping the geographic distribution of marine species [1, 9, 44, 45]. Therefore, we also included the maximum surface and bottom temperatures and the minimum bottom temperature during the preceding twelve-month period for each haul, which were extracted from the SODA data set based on the date and location of each haul. Minimum surface temperature was excluded because it was highly correlated with minimum bottom temperature (slope(se) = 1.027(0.001), *P* < 0.001, *r*^*2*^ = 0.87, *DF* = 136126).

The distribution of marine species on the continental shelf can also be influenced by seafloor rugosity (a measure of the spatial variation in depth) and sediment type [27, 46]. To calculate seafloor rugosity, we obtained depth data from the GEBCO gridded bathymetric data set for the North American shelf at ~1 km resolution [47]. Rugosity was calculated as the mean of the absolute differences between a given grid cell depth and the depths of the eight surrounding cells using the *raster* package in R [48]. To reduce computation time, rugosity was aggregated by averaging to a ~5.6 km resolution (0.05° latitude x 0.05° longitude). Aggregated rugosity values were highly correlated with the non-aggregated values (slope(se) = 0.872(0.001), *P* < 0.001, *r*^*2*^ = 0.77, *DF* = 148535). Rugosity values were assigned to individual hauls from the trawl surveys based on location.

Grain size was used to characterize seafloor sediment types throughout the sampling area using the Wentworth Phi (Φ) scale, where lower values indicate coarser sediments and higher values indicate finer sediments. Sediment data were obtained from multiple sources (Table 2) and mostly consisted of point estimates. Where sediment data in a region had only verbal or categorical descriptions, these were assigned to Folk codes [49] based on naming conventions in the U.S. Chart [50]. These were subsequently matched to quantitative sediment estimates using relationships between grain size, percentage gravel, mud, and sand (%GSM), and Folk codes developed using the full usSEABED database (117,031 point observations) for the Atlantic shelf [51], Gulf of Mexico [52], and U.S. West Coast [53]. In the Gulf of Mexico, grain size for each polygon was calculated from %GSM using Φ values for each pure sediment type (e.g., gravel Φ = -2.7, sand Φ = 1.7, and mud Φ = 7.5). Point estimates for grain size and %GSM were interpolated using inverse distance weighting in the *gstat* package [54, 55] to match the spatial resolution of the rugosity data.

**Table 2:** Sediment data sources.

Region	Source	Ref	Type	Data
U.S. Atlantic Coast	CONMAP	a	Polygon	Categorical
U.S. Atlantic Coast	ecstdb2005	b	Point	Grain Size; %GSM
Gulf of Mexico	Gulf of Mexico Data Atlas	c	Polygon	%GSM
U.S. West Coast	usSEABED	d	Point	Grain Size; %GSM
Eastern Bering Sea	ebssed	e	Point	Grain Size; %GSM
Gulf of Alaska	Gulf of Alaska Digitization Project	f	Point	Verbal
Aleutian Islands	AFSC	g	Point	Verbal
Gulf of St. Lawrence	Loring and Nota (1973) [56]	h	Digitized polygon map	Categorical
Scotian Shelf; British Columbia; Labrador	Natural Resources Canada Expedition Database	i	Point	Grain Size, %GSM
Newfoundland	Sediment Thickness Database	j	Polygons	Categorical

The source and type of data used for quantifying sediment characteristics on different regions of the North American continental shelf. Web links to each source are listed below. %GSM indicates data consisting of percentage gravel, sand, and mud.

^a^http://pubs.usgs.gov/of/2005/1001/data/conmapsg/conmapsg.zip

^b^http://pubs.usgs.gov/of/2005/1001/data/surficial_sediments/ecstdb2005.zip

^c^https://gulfatlas.noaa.gov/

^d^https://pubs.usgs.gov/ds/2006/182/

^e^https://data.noaa.gov/dataset/dataset/ebssed-database-surficial-sediments-of-the-eastern-bering-sea-continental-shelf

^f^https://www.sciencebase.gov/catalog/item/5699855be4b0ec051295ed8b

^g^http://www.afsc.noaa.gov/RACE/groundfish/Bathymetry/Aleutians.htm

^h^http://www.dfo-mpo.gc.ca/Library/1493.pdf

^i^http://ed.gdr.nrcan.gc.ca/grainsize_e.php

^j^http://geogratis.gc.ca/api/en/nrcan-rncan/ess-sst/97fc16ab-aadc-52f5-b33a-9145a78dd21c.html

### Species distribution modeling

To model the observed niche of individual species, we used a two-stage generalized additive model (GAM) approach [3, 4, 24] with the *mgcv* package in R [57]. GAMs were used because they require no *a priori* assumptions about the shape of the relationship that a given species has with a predictor variable and because they allow for nonlinear associations with habitat features [58]. For each species, models were fitted by using either the combined survey data from the east coast (including the Gulf of Mexico) or from the west coast U.S., including all seasons. By including all survey regions and seasons into a single niche model, we were able to more completely describe the range of thermal conditions in which a species is found [24, 40]. For species occurring on both U.S. coasts, the Atlantic and Pacific Ocean distributions were modeled separately.

For each species, the first-stage GAM was fitted to presence and absence data, and assumed a binomial error distribution. The second-stage GAM was fitted to log transformed biomass from non-zero observations and assumed Gaussian errors [3, 4, 24]. Predictor variables for each model included seasonal bottom and surface temperatures, annual minimum and maximum bottom temperatures, annual maximum surface temperature, seafloor rugosity and sediment grain size. Additionally, a categorical indicator for ecological survey was included to account for differences in sampling gear and methods between surveys (i.e., differences in survey catchability), which is a common method for standardization of catch data [59, 60]. We modeled ecological survey as a fixed effect because our data included a majority of the large-scale continental shelf surveys in North America, as opposed to randomly drawing from a population of surveys where random effects might be more appropriate. Further, initial trials indicated that using random effects would greatly increase computer processing time for niche modeling. A majority of the trawl surveys used have had vessel changes during the survey history or they employ multiple vessels each year. Vessel effects can influence catch and this can vary among species and years [61]. However, our niche modeling approach did not include vessel effects in order to reduce model complexity. Thus, our approach assumes that while catchability may vary among vessels, this variability will appear in the error term and does not interact with the environmental variables we are interested in. Depth was not included as a predictor variable so that projections into the future could allow for species to shift into deeper water [27], which has been observed to occur as a result of ocean warming [3, 4, 14]. Previous research has indicated that including depth as a predictor would greatly limit the ability of these models to explain historical shifts in depth, and that models without depth have greater explanatory power [3]. Further, a majority of the included species had survey observations that occurred throughout the sampled areas. Thus our approach assumes that any apparent relationship that species have with depth is driven by temperature variables and seafloor characteristics. The likelihood of overfitting the GAMs was reduced by including a gamma setting during model fitting, which acts as a penalty against model complexity. Gamma for each GAM was set to the log of the number of samples divided by two [58]. Predictions of biomass from the two-stage GAMs were calculated using the product of the predicted probability of occurrence and the exponentiated prediction of log-biomass.

Several criteria were used to determine which species to include in projections. First, we limited niche model fitting to species that had at least 250 occurrences within the combined survey data, which resulted in 703 species that were included for niche modeling. Second, we fitted a presence-absence model for each species, as described above, to a training data set that consisted of the initial 80% of hauls that occurred within each region. The remaining 20% of observations were used as a testing data set. The area under the receiver operator curve (AUC) was calculated using predicted and observed responses from the testing data with the *dismo* package in R [62]. Fourteen species were dropped from the analysis based on AUC scores below 0.75 [63] and three other species were dropped because observations were restricted to the testing data set.

### Projecting species distributions

Output from sixteen fully coupled general circulation models (GCMs) that participated in the Coupled Model Intercomparison Project 5 (CMIP5) were used to generate a range of projections for ocean temperature changes over the 21^st^ century (Table 3). For each GCM, we used output from simulations that were run under two future greenhouse gas emissions scenarios: a “strong mitigation” (RCP 2.6) and a “business as usual” scenario (RCP 8.5)[64]. The latter predicts continued global warming in excess of 4°C by 2090, while the former is expected to lead to global warming that is roughly consistent with the 2°C target of the Paris Agreement [17].

**Table 3:** Climate projection models.

Model	Modeling Center
bcc-csm1-1-m	Beijing Climate Center, China Meteorological Administration, China
bcc-csm1-1	Beijing Climate Center, China Meteorological Administration, China
CanESM2	Canadian Centre for Climate Modelling and Analysis, Canada
CCSM4	National Centre for Atmospheric Research, U.S.A.
CESM1-CAM5	National Science Foundation, Department of Energy;
	National Center for Atmospheric Research, U.S.A.
CNRM-CM5	Centre National de Recherches Meteorologiques, France
GFDL-CM3	Geophysical Fluid Dynamics Laboratory, U.S.A.
GFDL-ESM2M	Geophysical Fluid Dynamics Laboratory, U.S.A.
GFDL-ESM2G	Geophysical Fluid Dynamics Laboratory, U.S.A.
GISS-E2-R	NASA Godard Institute for Space Studies, U.S.A.
GISS-E2-H	NASA Godard Institute for Space Studies, U.S.A.
IPSL-CM5A-LR	L’Institut Pierre-Simon Laplace, France
IPSL-CM5A-MR	L’Institut Pierre-Simon Laplace, France
MIROC-ESM	Japan Agency for Marine-Earth Science and Technology;
	Atmosphere and Ocean Research Institute (The University of Tokyo);
	National Institute for Environmental Studies, Japan
MPI-ESM-LR	Max Planck Institute for Meteorology, Germany
NorESM1-ME	Norwegian Climate Centre, Norway

The 16 general circulation models used for climate projections including the laboratory of origin. The spatial resolution of each model can be found at: https://portal.enes.org/data/enes-model-data/cmip5/resolution

Each GCM was first regridded to match the finer spatial resolution of the SODA3.3.1 data (0.25° latitude and longitude at the equator), and the depth strata for projecting bottom temperatures was refined according to this finer spatial resolution of bathymetry. The delta method was used to downscale surface and bottom temperatures from the CMIP5 models. For this procedure, we first calculated the difference (i.e., delta value) between future temperatures and a modeled baseline period (mean of 1995–2014) with each GCM and for each scenario. These delta values were then added to a mean temperature climatology developed from the SODA3.3.1 data for 1995–2014. For the SODA grid cells outside the domain of a CMIP5 model, we used the nearest CMIP5 grid cell. For a majority of the GCMs (*N* = 12), 5% or less of the SODA grid cells needed to be populated in this fashion. Four of the GCMs had more restricted coverage on the continental shelf and between 18 and 35% of grid cells needed to be populated with neighboring cells. Finally, the climate projection grid was refined to 0.05° latitude x 0.05° longitude based on the spatial grain of the rugosity and sediment variables. We assumed that rugosity and sediment variables would be constant for the 21^st^ century and predictions were limited to depths shallower than 401m. Our resulting projection grid for the North American shelf consisted of 65,826 individual cells on the Pacific coast, 69,209 cells on the Atlantic coast, and 13,383 cells in the Gulf of Mexico, which was separated from the Atlantic coast at -80.75° longitude off the southern tip of Florida. For each species, we generated 32 projection series (16 GCMs × 2 RCPs) of annual biomass estimates from 2007 to 2100 for each grid cell during the summer season (July-September). Data from each series were aggregated by averaging projections within twenty-year bins. Projections ran for 7.7 days on Centauri, a 640-core computer cluster in the School of Environmental and Biological Sciences at Rutgers University.

### Analysis and uncertainty estimation

For each species, we calculated the centroid for the present time period (2007–2020) and the end of the century (2081–2100) for each GCM and RCP. We calculated the centroid as the predicted biomass-weighted mean latitude and longitude [3]. Projection grid cell areas decline towards the poles due to converging longitudes and this was factored into centroid calculations using the *raster* R package [48]. Centroid calculations were conducted separately for the Gulf of Mexico and the Atlantic coast for species with historic survey observations in both regions. However, species that only had historic observations in the Gulf of Mexico were analyzed in that region only. From initial and future centroid locations, we calculated the predicted distance shifted in km and the direction of the shift in degrees using the *geosphere* R package [65]. The change in latitudinal centroid was also calculated. We then calculated the mean and standard deviation among GCM projections within an RCP for distance shifted and change in latitude. We were not able to calculate shift distances that followed the coastline, as is often done at smaller spatial scales [14], because there was no simple path northward that spanned our projection grid on either coast. Centroids were not confined to the projection grid, and for this study, we used projected shifts in centroids to indicate the magnitude of change in species’ distributions.

We also calculated a metric for directional agreement among GCM projections by first converting each projected shift direction into Cartesian coordinates on a unit circle of radius one. We then averaged these Cartesian coordinates across GCMs and calculated the radius of that mean position from the origin as an indicator of directional agreement. A radius of zero indicated a complete lack of agreement in shift direction, and a value of one indicated that all GCM predictions pointed in an identical direction.

We used two methods that we termed distance-directional uncertainty (DDU) and latitudinal uncertainty (LU) to identify which species had relatively robust projections of distribution changes (strong agreement among models) and which species had poor agreement among projections. DDU combined metrics for variation in projected shift distance and direction among the sixteen GCMs. For DDU, we first used linear regression to relate the standard deviations of shift distance to the mean predicted shift distances (km) of projected species. The regression was done on a log-log scale to normalize residual errors. We then used the residual error values from this regression model to indicate the relative uncertainty in shift distance among species, where positive (negative) residuals indicated projections that were more (less) uncertain than would be expected given the distance shifted. The residual error values were then plotted against values for GCM directional agreement. Species falling above the 75^th^ percentile for either the residual error values from the regression or for directional agreement were considered to have medium uncertainty, while species falling above the 95^th^ percentile were considered to have high uncertainty.

Latitudinal uncertainty (LU) was calculated by regressing the standard deviations among the sixteen GCM predictions against the absolute values of mean predicted shifts in latitude. This was done on a log-log scale to normalize residual errors. Quantile regression, using the *quantreg* package in R [66], of the 95^th^ and 75^th^ percentiles was used to indicate species with high or medium uncertainty, respectively. For each RCP, species classified as “high uncertainty” with either the DDU or LU methods were considered to have poor model agreement among projections, while species with “low uncertainty” for both methods were considered to have robust projections. The low uncertainty species were then used to make comparisons of assemblage-scale shifts among the major oceanic basins of North America.

While centroid calculations reflect spatial patterns of thermal habitat, they do not elucidate the influence of climate change on the overall amount of thermal habitat available. Therefore, for each GCM we calculated the projected change in total mean annual thermal habitat during the 21^st^ century. Average annual thermal habitat availability was calculated as the sum of all projected biomass values (biomass per swept area × grid cell area) from the projection grid. Predicted change was calculated as a percentage of the mean predicted thermal habitat for the 2007–2020 period. The mean percentage change in annual thermal habitat of the sixteen GCMs was calculated for each species that was classified as low uncertainty.

Our approach for projecting shifts in species distribution and biomass modeled only the changes in thermal habitat for each species and did not include other important factors like the influences of fishing or changes in primary productivity and carbonate chemistry on species distribution and abundance. In this way, our projections are only projections of potentially suitable habitats. Interpreting our projections of thermal habitat as projections of species distributions assumes that species are able to colonize all thermally suitable habitat within our projection region and that shifts are not limited by reproduction and recruitment dynamics. Our goal was to isolate temperature effects, and thus provide an indication of the anticipated magnitude of changes among species and regions as a result of climate change in the 21^st^ century. Further, we use the term ‘thermal habitat availability’ (alt. ‘thermal habitat’) to indicate that we are not making future predictions of absolute biomass.

## Results

### Species distribution model summaries and uncertainty analysis

A total of 383 Atlantic species and 303 Pacific species met the criteria during thermal niche model fitting for conducting projections. This list of species included teleost fishes, elasmobranchs, three hagfishes, a species of chimaera, a variety of crustaceans, cephalopods, echinoderms and other invertebrates, and a species of sea turtle (S1 Appendix). The number of occurrences within the survey data ranged among species from 298 to 35018, with a median value of 1359 occurrences. The amount of variation in survey data that was explained by the thermal niche models varied widely by species. For the presence-absence GAMs, all species had AUC values greater than 0.79, with a median value of 0.94 among species. The percentage of deviance explained for the presence-absence GAMs ranged from 11.7 to 77.7%, with a median value of 38.3%. For the logged biomass GAMs, the percentage deviance explained ranged from 3.3 to 98.6% and the median value was 90.0%.

**Fig 2:**
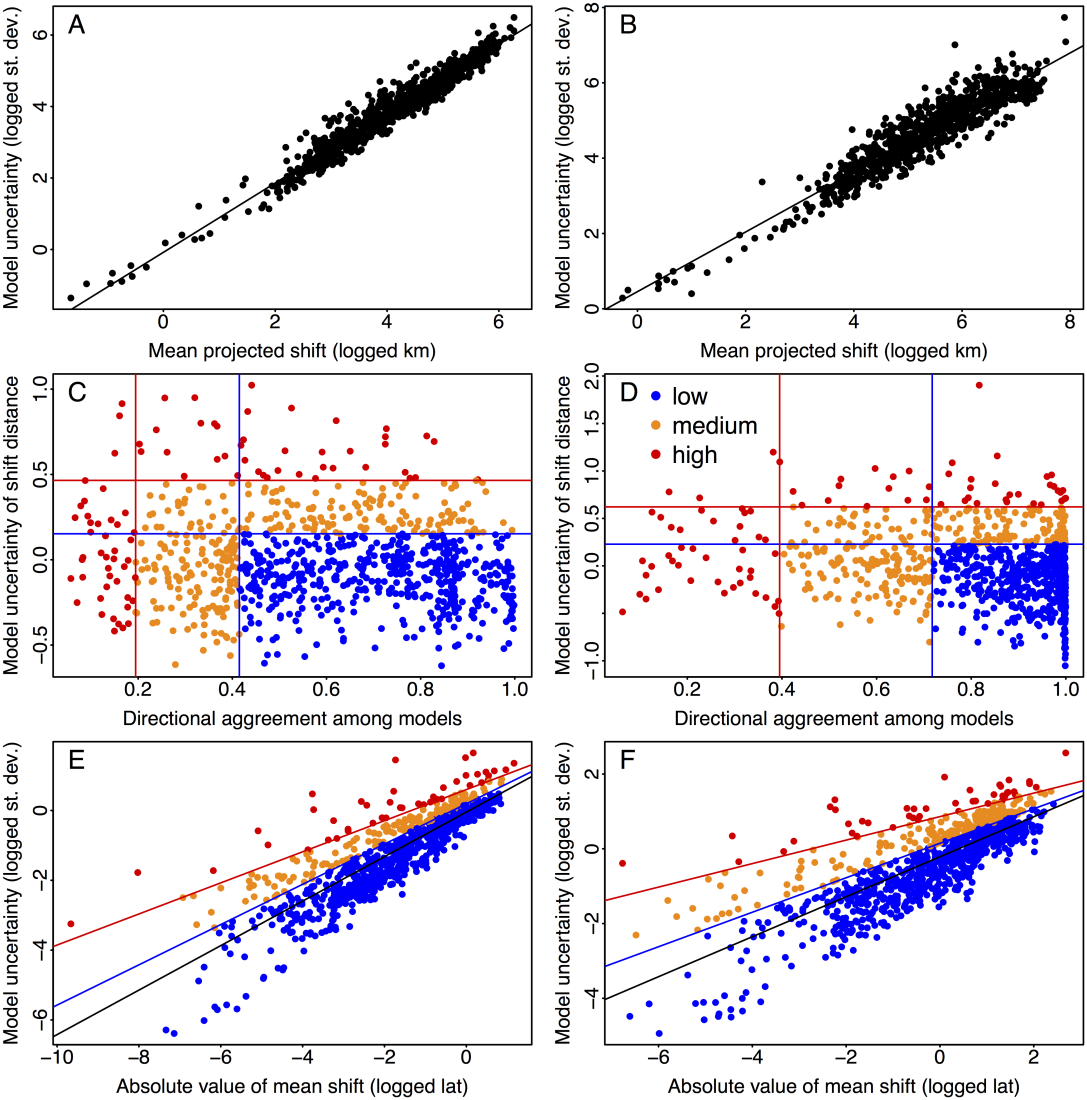
Species projection uncertainty. Categorizing uncertainty for 878 species projections within the RCP 2.6 (A, C, and E) and RCP 8.5 (B, D and F) scenarios. (A and B) The logged standard deviation of projected shift distance plotted against the logged mean shift distance in km among 16 GCMs. (C and D) The distance-directional uncertainty (DDU) method where the residual error values from panels (A) and (B) were plotted against shift directional agreement among GCMs. (E and F) The latitudinal uncertainty (LU) method where the logged standard deviation in projected shift in latitude was plotted against logged mean shift in latitude among 16 GCMs. Red and blue lines indicate the 95^th^ and 75^th^ percentiles (C and D) or quantiles (E and F), respectively. Points are colored according to their level of uncertainty where red indicates high uncertainty, orange is medium, and blue is low uncertainty (C–F).

Species varied greatly in how much the 16 GCMs agreed in their distribution projections. The standard deviation of total centroid shift distance among GCMs increased linearly with mean predicted shift for both RCPs (Fig 2A and 2B). For the DDU metric, residual values from these linear relationships were plotted against the directional agreement among GCM predictions (Fig 2C and 2D). Directional agreement among predictions was much higher for RCP 8.5 projections (75^th^ percentile at 0.72) than for RCP 2.6 (75^th^ percentile at 0.41). This difference among RCPs was primarily due to many species with small projected shift distances under RCP 2.6, which allowed for more variation in direction among GCMs. The DDU method categorized 498 (507) species projections as low uncertainty for RCP 2.6 (RCP 8.5), 295 (286) as medium uncertainty, and 85 (85) as high uncertainty. Note that some species have separate projections for the east coast and the Gulf of Mexico, which is why the total exceeds 686 species for each RCP.

The standard deviation of predicted latitudinal shifts (positive or negative) of centroids among GCMs was linearly related to absolute values of the mean predicted shifts in latitude (Fig 2E and 2F). For categorizing species based on latitudinal uncertainty (LU) for the RCP 2.6 (8.5) scenario, our method grouped 658 (658) species as low uncertainty, 175 (177) as medium uncertainty, and 45 (43) as high uncertainty.

There was a significant association between our two methods (DDU and LU) for categorizing species projection robustness (Pearson’s Chi-square test for RCP 2.6: *Χ*_4_ = 97.84, *P* < 0.001; RCP 8.5: *Χ*_4_ = 141.4, *P* < 0.001). For RCP 2.6 projections, 59% of species had an identical uncertainty rating (i.e., high, medium or low) for each method, and for RCP 8.5 projections 62% of species had identical ratings. Based on combining DDU and LU methods for identifying species with relatively robust projections, for RCP 2.6 (RCP 8.5) we grouped 429 (448) species as low uncertainty, 329 (314) as medium uncertainty, and 120 (116) as high uncertainty (Table 4; S1 Appendix). The level of projection uncertainty for RCP 2.6 was affected by how often a species was encountered in survey data (ANOVA: *F*_2, 875_ = 7.714, *P* < 0.001) and species in the medium and high uncertainty categories had significantly fewer observations than low uncertainty species. Among the RCP 8.5 projections there was no difference in the number of survey observations between the low, medium and high uncertainty groups (*F*_2, 875_ = 0.004, *P* = 0.99).

**Table 4:** Projection uncertainty of species by region.

Region	RCP 2.6			RCP 8.5		
	Low	Medium	High	Low	Medium	High
Eastern Bering Sea	84	48	12	99	31	14
G. Alaska-W. Canada	71	22	6	61	29	9
E. Canada	67	32	12	62	34	15
Northeast U.S.	17	13	8	8	20	10
West U.S.	34	16	10	35	18	7
Southeast U.S.	80	71	36	100	64	23
Gulf of Mexico	76	127	36	83	118	38
**Total**	429	329	120	448	314	116

Number of species categorized as low, medium, and high uncertainty within each RCP scenario by region.

### Projections of centroid shifts

**Fig 3:**
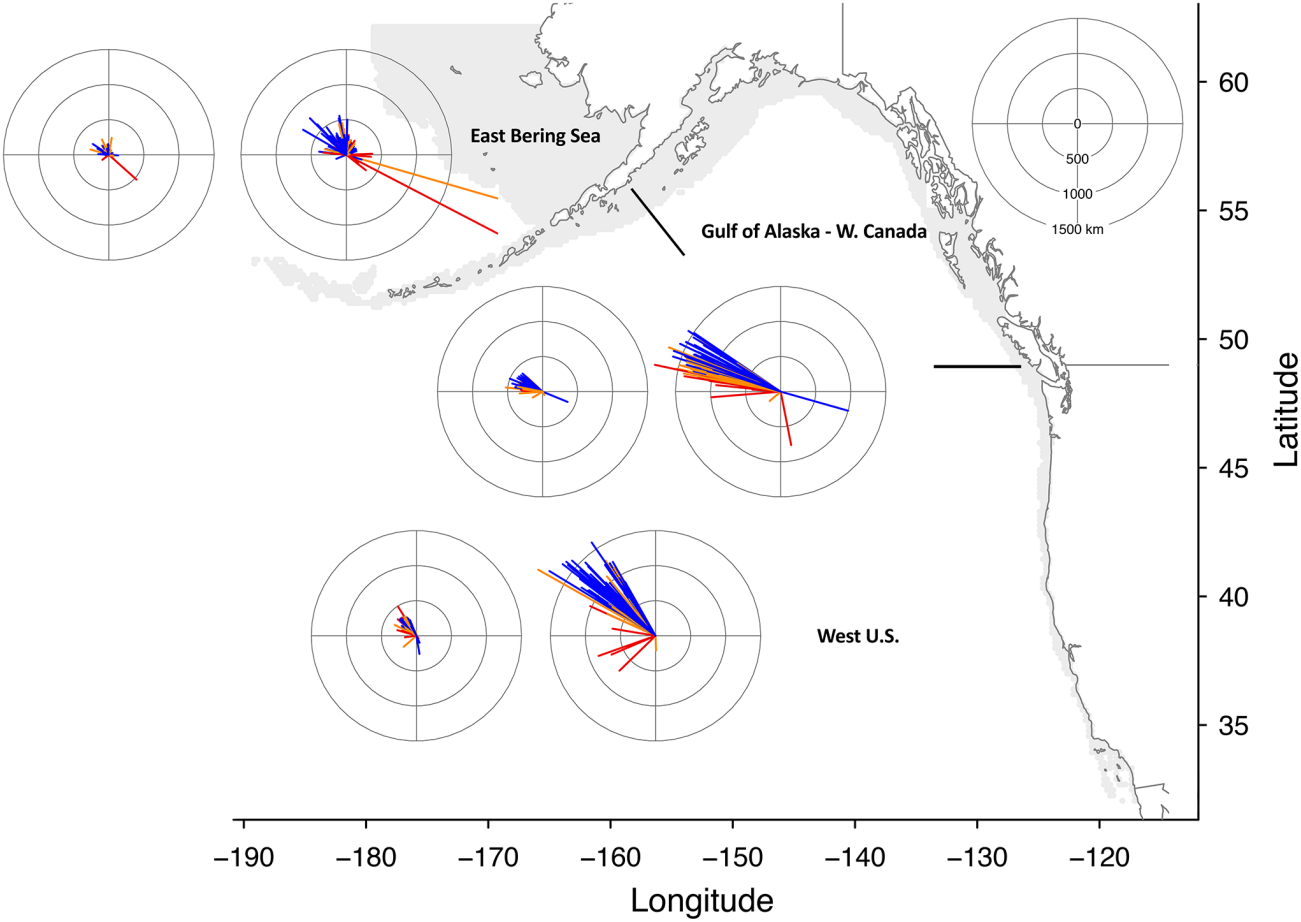
Projected shifts in distribution for west coast species. The direction and magnitude in km of projected shifts in centroids for 303 species on the North American west coast. Species were grouped into regions based on projected origin of centroid averaged over 2007–2020, but all projections were on a coast-wide scale. Each regional pair of plots consists of RCP 2.6 projections on the left and RCP 8.5 projections on the right. Projections colored blue indicate low uncertainty, orange indicates medium uncertainty, and red indicates high uncertainty. Note that the distance scales on the compass plots and that of the map do not match. The gray area on the map indicates the projection area on the continental shelf.

**Fig 4:**
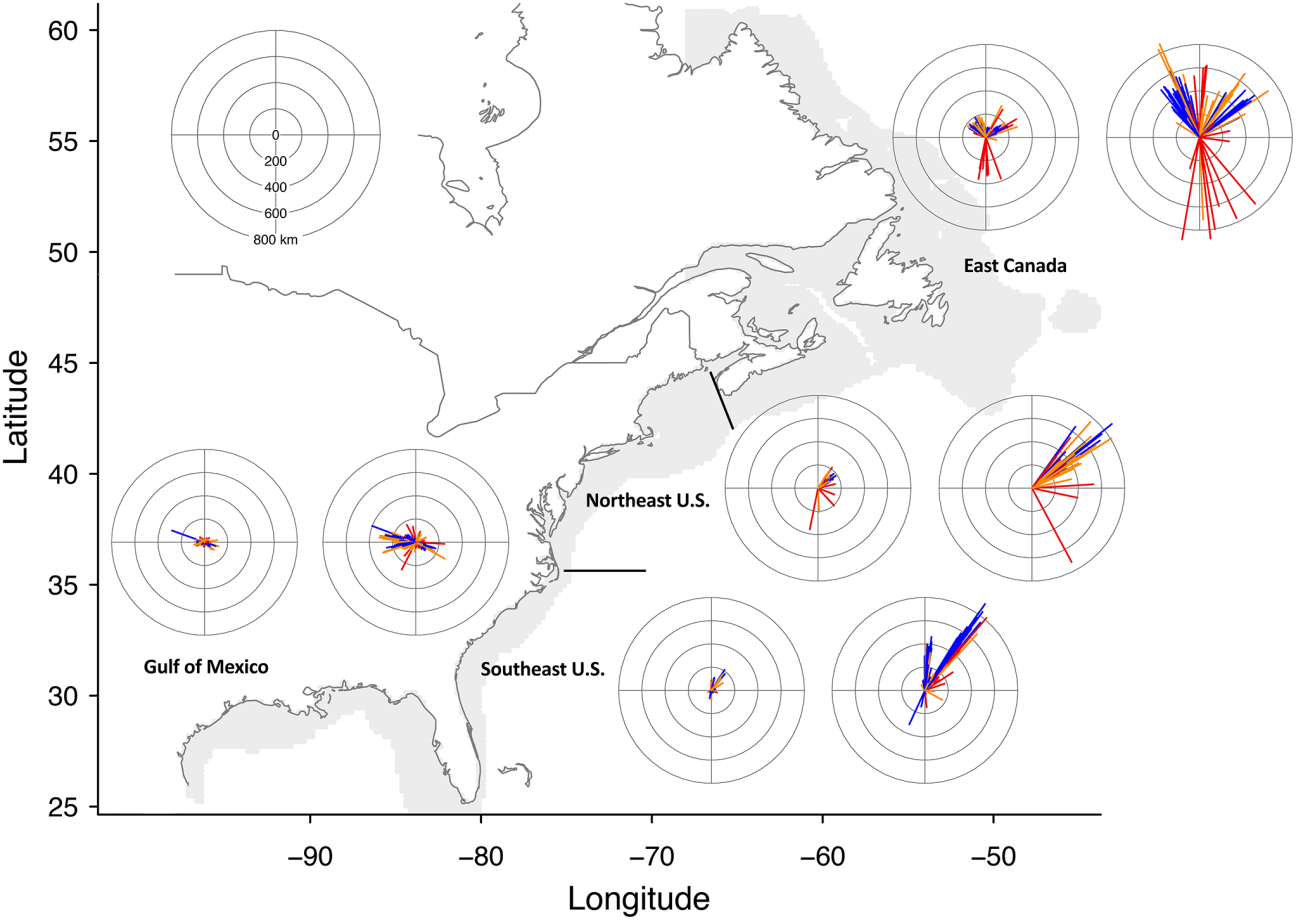
Projected shifts in distribution for east coast species. The direction and magnitude in km of projected shifts in centroids for 336 species on the North American east coast and 239 species in the Gulf of Mexico. For additional detail refer to Fig 3 caption. Note that the scale of the compass plots for the east and west coasts differ.

For presenting the projections of species centroid shifts, we grouped species into seven regions based on projected centroid location for the 2007–2020 time period (Figs 3 and 4). However, projections for all species were conducted at a coast-wide scale. Under the RCP 2.6 emissions scenario, the low uncertainty projections of species centroid shifts were generally less than 200 km (Figs 3 and 4). However, multiple projections for RCP 2.6 in the Gulf of Alaska and West Coast U.S. exceeded 300 km (Fig 3). The magnitudes of shifts were much larger under the RCP 8.5 scenario, and multiple species shifts exceeded 1500 km on the west coast and exceeded 600 km on the east coast (Figs 3 and 4). Projected shifts generally followed the coastline towards increasing latitude. For example, species that were primarily caught in the U.S. Northeast Shelf shifted to the northeast, while species originating from the Gulf of Alaska shifted west and north. Species from the northernmost regions, such as Eastern Bering Sea and Newfoundland, had smaller projected shifts, which is probably the result of these regions being constrained by the northern boundary of our projection region. Projections in the Gulf of Mexico tended to shift westward or towards the southeast and were generally of lower magnitude, which probably was the result of this region being constrained by the U.S. coast.

Within regions, there was wide variation in species’ projected responses to ocean warming, both in shift direction and magnitude (Figs 3 and 4). For example, species originating from the Gulf of Alaska and the west coast of Canada that had low uncertainty projections ranged in projected shift distance from 267 to 1630 km under RCP 8.5. Generally, projections among species with low uncertainty had a high level of agreement in shift direction. Although in some regions, shifts clustered in more than one general direction. For example, in the southeast many tropical species expanded northward into this region from the Florida shelf, while another group of species originating from the southeast shifted towards the northeast into the mid-Atlantic U.S. region. Conversely, shifts in atypical directions for a region were most often categorized as medium or high uncertainty, illustrating the low level of GCM model agreement for these species (Figs 3 and 4). West coast projected shifts were generally more robust; in particular species originating from the Gulf of Alaska and Eastern Bering Sea regions tended to have robust projections, with both regions having at least 62% of species categorized as low uncertainty for both RCPs (Table 4). Conversely, in the Gulf of Mexico and Northeast U.S. regions, less than 45% of species had low uncertainty projected shifts for both RCPs.

**Fig 5:**
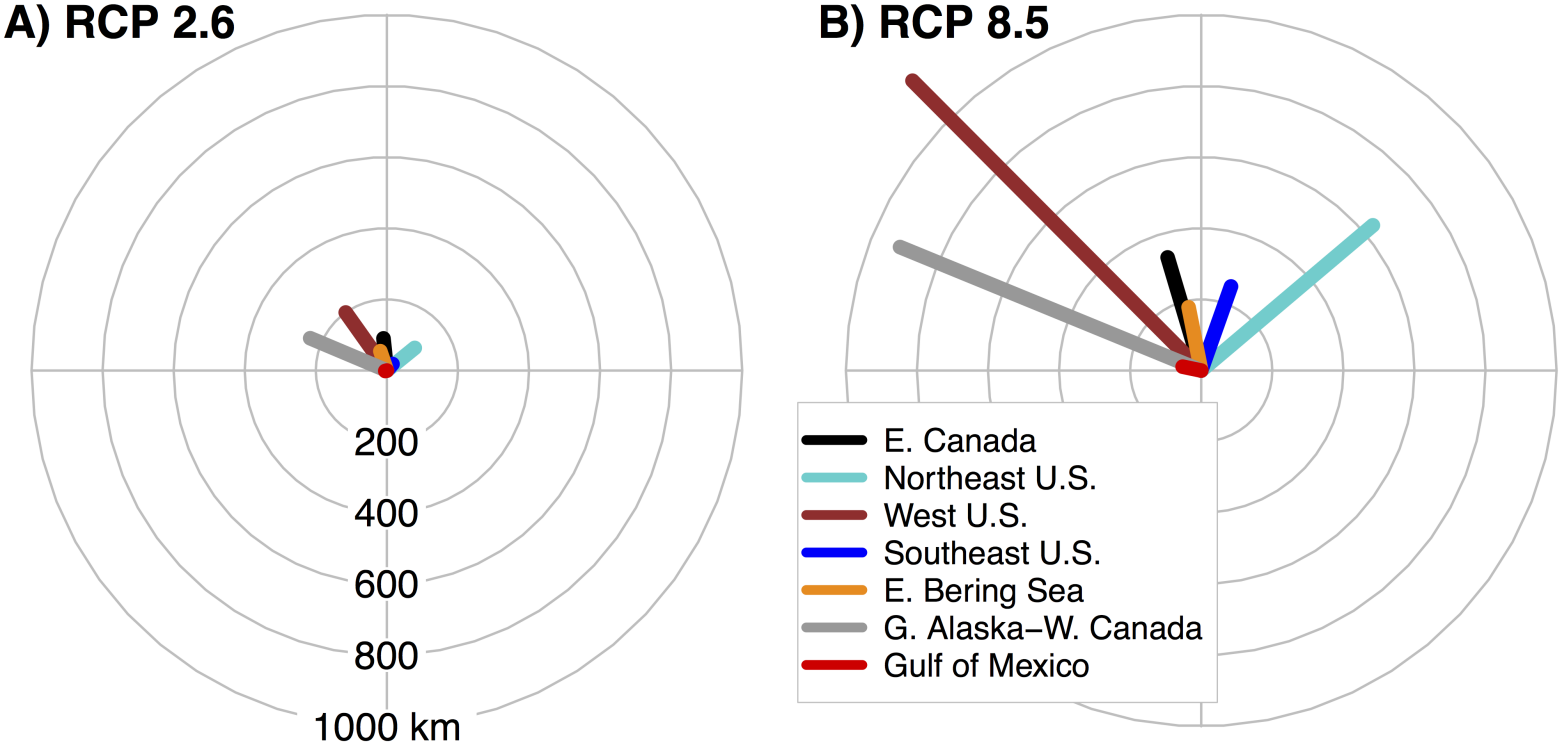
Regional difference in projected distribution shifts. Mean direction and magnitude of projected centroid shifts over the 21^st^ century for low uncertainty species originating from seven regions of the North American shelf for (A) RCP 2.6 and (B) RCP 8.5.

To compare among regions, we only considered species with low uncertainty projections, and combined both shift distances and directions to calculate the average assemblage shift for each region. Under RCP 2.6, the mean shift distance across species was greatest for West Coast U.S., and Gulf of Alaska and West Coast Canada species (mean shift distance of 224 and 248 km, respectively) (Fig 5A). In contrast, species originating from the Gulf of Mexico and the Southeast U.S. both had mean shifts less than 40 km. For the RCP 8.5 scenario, the highest magnitude average shifts again occurred for the West Coast U.S. at 1162 km and the Gulf of Alaska at 954 km, but the Northeast U.S. also had relatively high magnitude projections at 637 km (Fig 5B). Species from the Gulf of Mexico again had the lowest magnitude projected shifts under RCP 8.5, which partly results from greater variation in projected shift direction among species (Fig 4).

### Projections of change in thermal habitat availability

**Fig 6:**
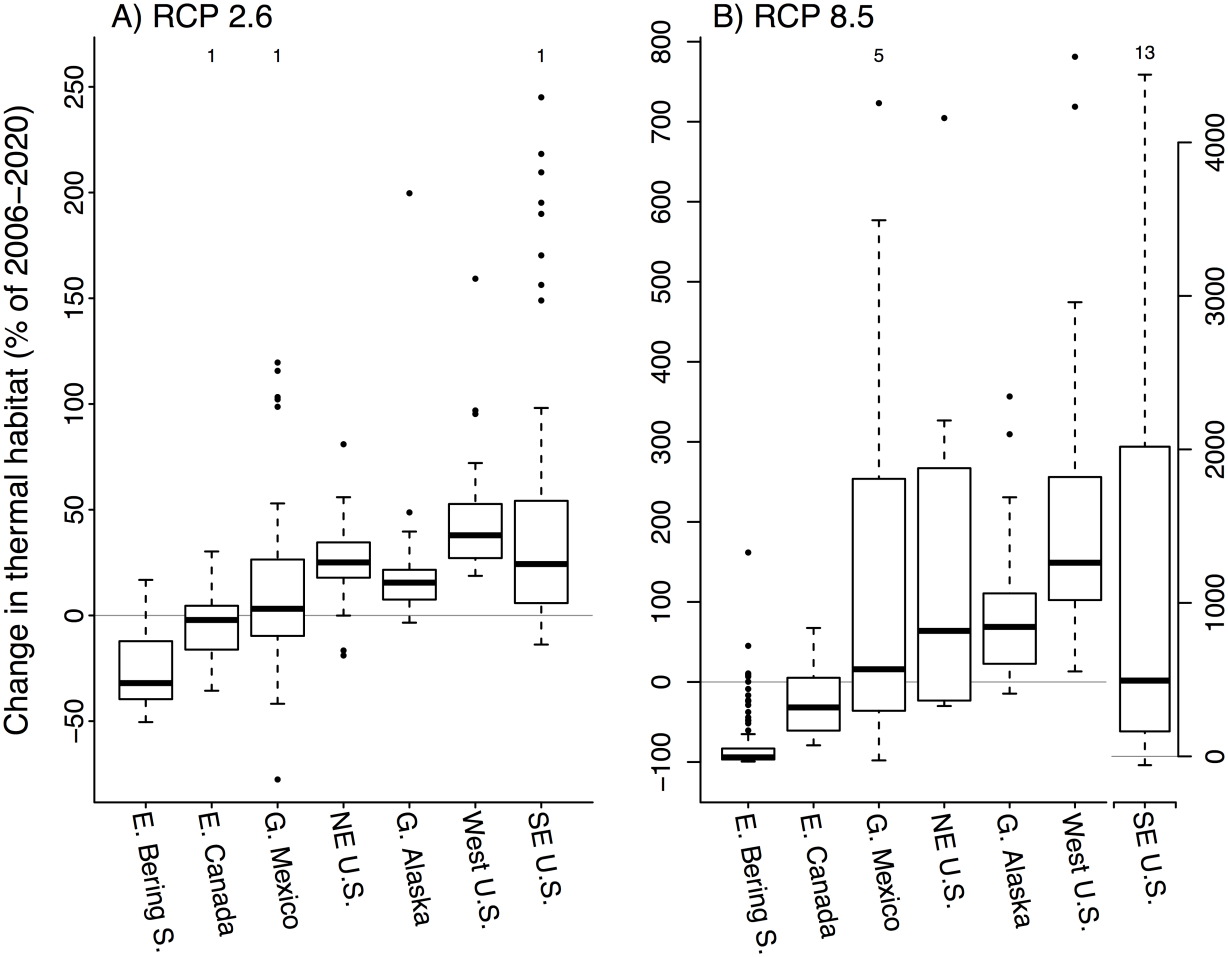
Projected change in thermal habitat availability. Mean percentage change in projected thermal habitat availability over the 21^st^ century for low uncertainty species originating from seven regions of the North American shelf for (A) RCP 2.6 and (B) RCP 8.5. Boxes indicate the median and the 25^th^ and 75^th^ percentiles, whiskers extend to within 1.5 of the interquartile range or to data extremes. Number of extreme data points occurring out of the plotting range is indicated for each region at the top. The right y-axis in (B) applies to SE U.S. only. Sample size for each region is provided in Table 4.

**Fig 7:**
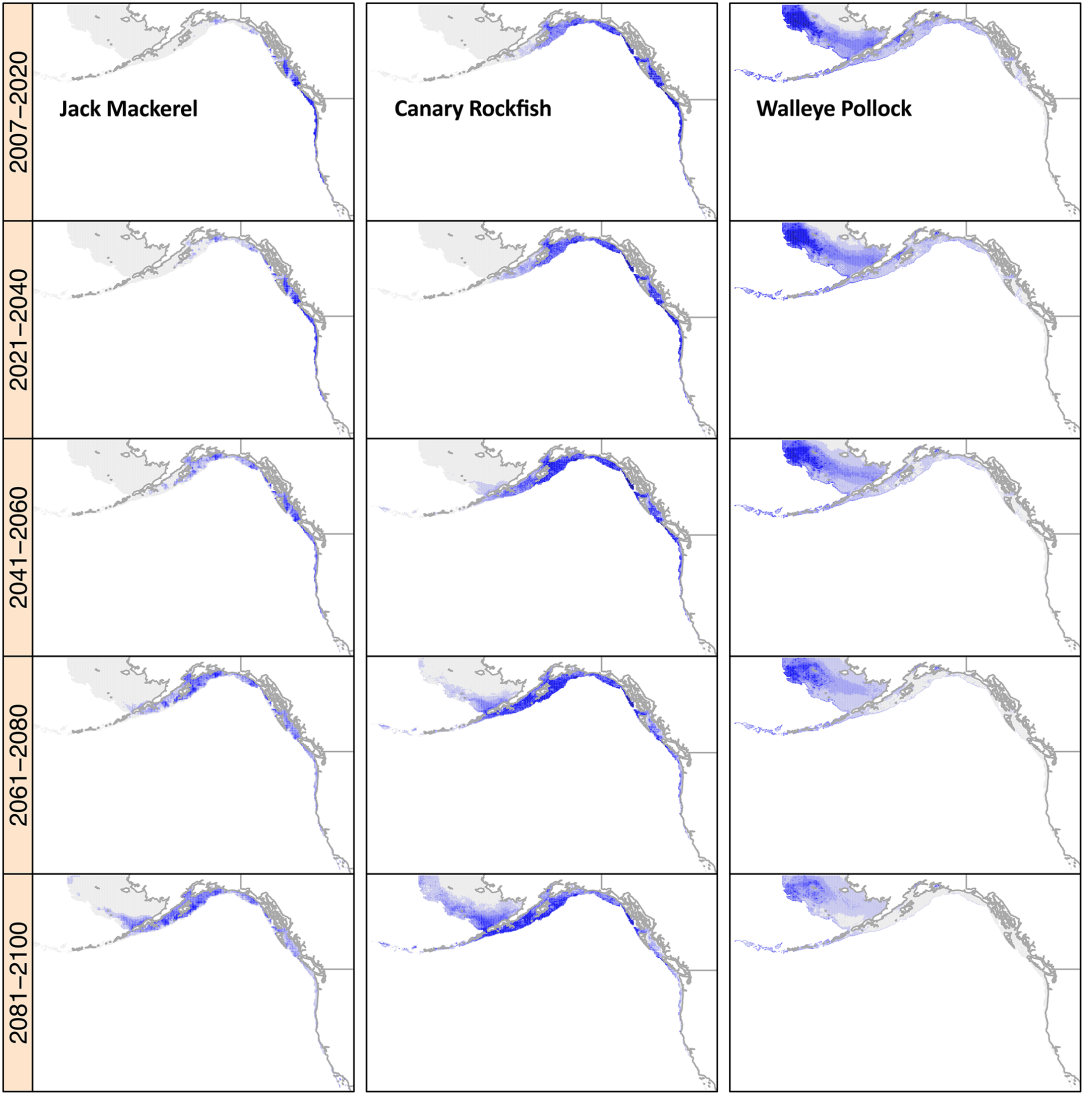
Examples of west coast species projections. Jack mackerel *Trachurus symmetricus* is in the left-most column, canary rockfish *Sebastes pinniger* is in the middle column, and walleye pollock *Theragra chalcogramma* is in the right-most column. Mean annual thermal habitat suitability during summer under RCP 8.5 is shown for twenty-year periods in the 21^st^ century. Habitat quality is higher in areas of greater blue intensity. Gray areas indicate regions of the projection grid that are not suitable thermal habitat. White areas indicate regions not included in the projections (either land or deep water).

**Fig 8:**
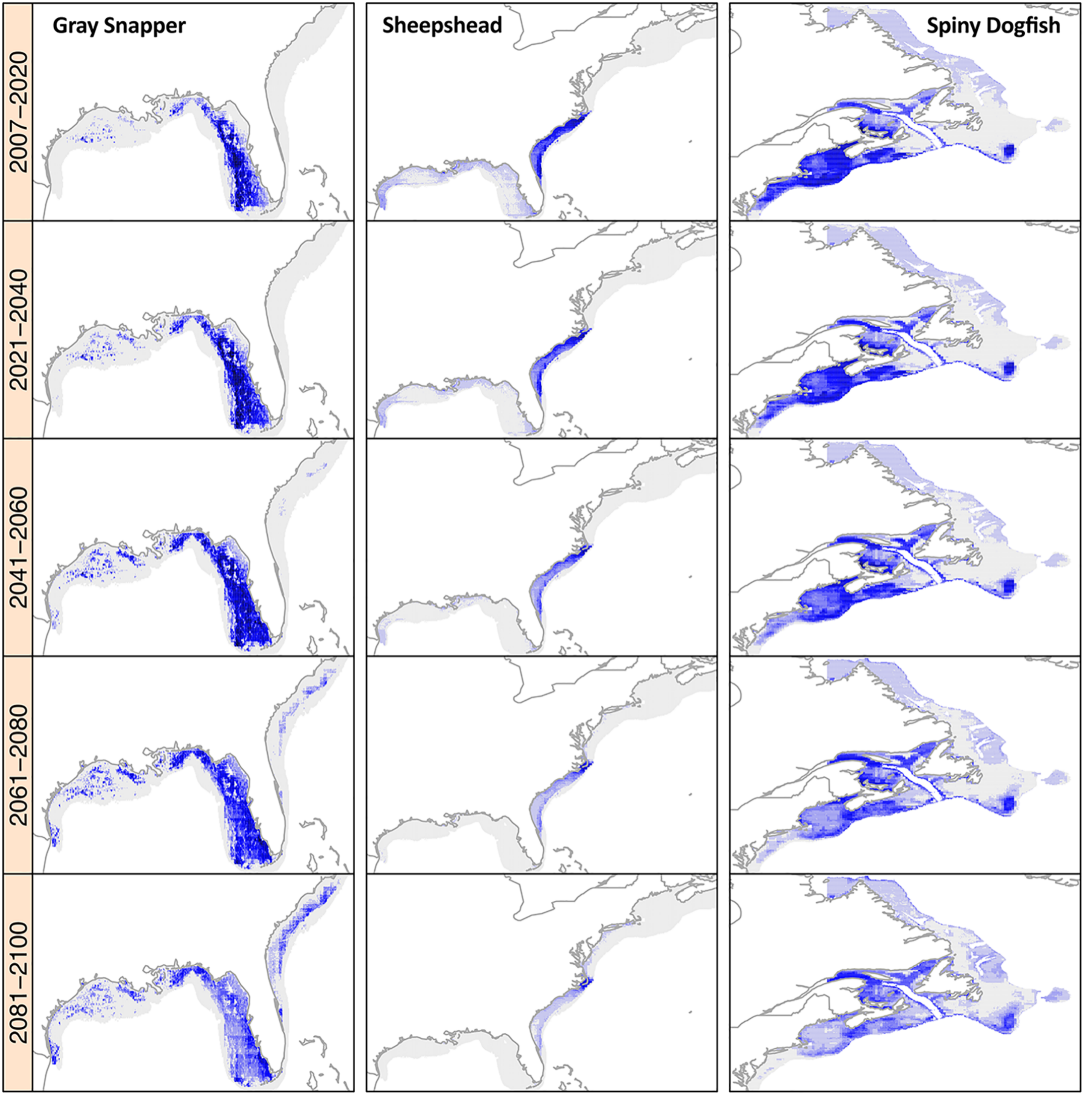
Examples of east coast species projections. Gray snapper *Lutjanus griseus* is in the left-most column, sheepshead *Archosargus probatocephalus* is in the middle column, and spiny dogfish *Squalus acanthias* is in the right-most column. For other details, refer to Fig 7 caption.

Predicted changes in the availability of thermal habitat over the course of the 21^st^ century were generally of greater magnitude, both positive and negative, for RCP 8.5 predictions as compared to RCP 2.6 (Fig 6). Many species were projected to experience overall increases in thermal habitat availability in North America over the 21^st^ century, particularly those from the Southeast, Northeast, and West U.S. Coasts, and the Gulf of Alaska and West Coast of Canada. These positive responses resulted from two major patterns. First, some species expanded into regions with larger areas of continental shelf habitat. For example, on the U.S. West coast, both jack mackerel (*Trachurus symmetricus*) and canary rockfish (*Sebastes pinniger*) had projected centroid shifts that exceeded 1300 km as they expanded into the Gulf of Alaska and Eastern Bering Sea, respectively (Fig 7). Associated with these changes in distribution were large (greater than 90%) predicted increases in thermal habitat availability. The second mechanism by which projections for thermal habitat availability increased was for species of tropical origin that expanded into the projection area as temperatures increased. Gray snapper (*Lutjanus griseus*) was initially most abundant west of Florida, but its thermal habitat expanded throughout the Gulf of Mexico (71% increase; Fig 8). In the Southeast U.S. shelf gray snapper was initially projected to have negligible habitat, and so the expansion of this species into the region by the end of the 21^st^ century led to a large estimate of increase in thermal habitat (96,663% increase), which was a common trend in this region (Fig 6).

Not all species that expanded northward had major increases in thermal habitat availability. Sheepshead (*Archosargus probatocephalus*) was projected to shift northward on the east coast by 2.4 degrees of latitude, but a 46% decline in total suitable habitat was predicted for this species because habitat gained in the Northeast U.S. shelf was still marginal (Fig 8). Temperatures became less favorable in the Gulf of Mexico for sheepshead resulting in a loss of 83% of initial habitat. The projected loss in habitat for sheepshead within the Gulf of Mexico was among the medium uncertainty group, despite having a low uncertainty projection on the Atlantic coast. Nevertheless, 43% of Gulf of Mexico species with robust projections also lost thermal habitat during the 21st century (Fig 6). Spiny dogfish (*Squalus acanthias*) was projected to have an increase in habitat suitability during the 21^st^ century in areas of the Newfoundland shelf and in the Gulf of St. Lawrence as the center of distribution shifted 483 km (Fig 8). However, spiny dogfish was predicted to have a 32% net loss of thermal habitat coast-wide due to a major decline in habitat availability in the southern half of its range.

Species common to the northern extreme areas of the Eastern Bering Sea and Eastern Canada shelf generally had negative trends in thermal habitat availability within our study region (Fig 6). The large predicted loss of thermal habitat for these northern species may partly result from their distributions moving out of our projection area. For example, walleye pollock (*Theragra chalcogramma*) was initially abundant throughout the Alaskan shelf, but by the end of the century it experienced a loss of 44% of thermal habitat and was projected to be largely restricted to the Eastern Bering Sea at reduced densities (Fig 7).

## Discussion

Water temperature is a major factor in determining the geographic distribution and preferred habitats of marine species, though the mechanism for these relationships is perhaps mediated by oxygen demand and availability [1, 3, 67, 68]. We have shown that climate change in the 21^st^ century will shift the location and available area of suitable thermal habitat for species inhabiting the North American shelf. These shifts in thermal habitat can interact in complex ways with seafloor characteristics and variations in the width of the continental shelf to produce a wide range of predicted species responses. Despite this strong variation among species and among climate models, the general trend was for poleward shifts in thermal habitat, which is similar to previous studies predicting changes in species distribution [23–25, 28, 31]. However, most previous studies have either taken a more regional approach to species projections and focused on changes in available habitat within the bounds of a biological survey, or have taken a coarse global approach. Here we have presented some of the first fine-grained projections for distribution changes that encompass the majority of the geographic ranges for hundreds of marine species in North America.

There are few existing studies that have also projected species distribution shifts at a larger geographic scale. Robinson et al. [23] found a mean projected poleward shift of ~400 km under a high emissions scenario for a group of nine pelagic species off eastern Australia by the year 2070. Our projection summaries were for the end of the 21^st^ century, but the projected shift distances from many of our regional assemblages were similar to Robinson et al. [23]. Cheung et al. [25] conducted projections for 1066 species globally and found that the median projected poleward shift of the center of marine species’ ranges was 79 km for a high carbon emissions scenario. This study projected shifts between 2000–2005 and 2040–2060 and used a single climate projection model. Further, the bioclimate envelope models used to project distribution shifts in their study were different than our approach, and incorporated population dynamics such as dispersal and population growth. Nevertheless, when we made a similar calculation for poleward shifts in latitudinal centroid among our projected species we found a median value of 164 km (~20.6 km per decade). Simple comparisons between our results and Cheung et al. [25] are difficult to make for multiple reasons (e.g., different species and regions examined, different climate models used), but considering that our projections are to the years 2081–2100, then the median latitudinal shift values between our studies are similar. More recently, Jones and Cheung [69] projected distribution shifts on a global scale for 802 marine species and compared three different approaches of presence-only niche modeling, using a single climate projection model. They found a median poleward shift of 25.6 km per decade for species centroids under RCP 8.5, which is similar to our value of ~20.6 km for North American species. While similarities among results from these projection studies are encouraging, there is still a poor understanding of how these different approaches to species niche modeling influence projections [36].

Species from the U.S. West Coast, Gulf of Alaska and West Canada Coast assemblages had relatively large predicted shifts in distribution, some exceeding 1500 km for the high emissions scenario (RCP 8.5). It is challenging to assess the realism of these projections by looking at historic trends in marine species distribution shifts, largely because available historical data are typically restricted to a region. However, even at a regional scale species distribution shifts greater than 10 km per year have been observed [3, 70], which would scale to 1000 km over a century. Further, ocean warming rates under the RCP 8.5 scenario are projected to be greater than what has been observed during the past four decades, the decades during which most long-term surveys were operational [71]. This suggests that our projections are likely consistent with historical observations.

The dramatic predictions for centroid shifts for Pacific species (excluding Eastern Bering Sea) might be expected because the spatial gradient of temperature change on the west coast is much weaker than on the east coast [12]. Consequently, the projected climate velocity (the rate and direction of isotherm shift) on the west coast of North America is predicted to have a much stronger along-shore and poleward trajectory than on the east coast [72]. This is important because predicted shifts in species distribution can be greater in areas with weak spatial gradients in temperature change [23]. Another reason for the large magnitude shifts among Pacific species is the greater continental shelf habitat available to the north. In other words, as habitat becomes suitable at latitudes corresponding to the Gulf of Alaska or Eastern Bering Sea, the greater amount of continental shelf in these regions results in major thermal habitat increases, which strongly influenced estimates for geographic centers of biomass.

Interestingly, the projected shifts of marine species for the U.S. West Coast assemblage represented a contrast to historic trends in this area, where ocean temperatures have been relatively static or even cooling in recent decades [12]. Consequently, the distribution of species in this region has been relatively stable compared to other North American areas [3]. Our predictions show a similar contrast for the Southeast U.S., where temperatures and species distributions have also been relatively static in recent decades [9], but where our projections suggested rapid northward shifts in the future.

Species common to the northern parts of our projection area had lower magnitude projections for shift distance, and projections for changes in thermal habitat availability were generally negative. However, many of these species may be able to redistribute northwards and out of our study region, where continental shelf habitat is also available. For example, in the Barents Sea, more temperate Atlantic species have been spreading into areas where seasonal sea ice occurs, displacing Arctic species [73]. We did not extend our projection grid towards Arctic waters due to a lack of biological survey data in these regions, along with a lack of data on sediment characteristics. However, Wisz et al. [74] used niche habitat modeling for north Atlantic and Pacific species and projected that Arctic regions will become suitable for many temperate species during the 21st century.

Predicted shift distances were also small in the Gulf of Mexico and there was the least agreement in shift direction among the low uncertainty species. This may be unsurprising as temperatures are more uniform across the Gulf of Mexico compared to other regions of the North American shelf, and there was not a strong along-shore directional gradient in our temperature climatology for this region. Further, projected isotherm shifts in this region are generally poleward as opposed to along shore [72]. As a result, shifts in this region were often driven by tropical species expanding throughout the region (e.g., gray snapper, Fig 8) or by species generally losing habitat (e.g., sheepshead, Fig 8). Also, some species were projected to become increasingly restricted to deeper habitats, which has been shown with historic observations from survey data [3, 4].

Despite the detailed data that went into our species distribution models, the models were fitted in an automated fashion and did not account for a detailed understanding of each species’ natural history. However, the benefit of an automated approach to model fitting is that we could make projections for a wide range of species. A closer examination of gray snapper (Fig 8) helps to illuminate the caveats of this approach. Climate change impacts for this species have been studied in detail, and so this offers a good opportunity for comparison. Hare et al. [31] predicted gray snapper to be resident on the U.S. Southeast coast to a maximum extent of between 31 and 31.5° N by the end of the century for a high greenhouse gas emissions scenario. Their study projected gray snapper distributions based on low temperature tolerance during winter through effects on overwinter survival in estuaries. Similarly, minimum annual bottom water temperatures were an important predictor for our gray snapper niche model. However, we predicted gray snapper to extend to ~34° N under RCP 8.5. This difference between studies suggests the importance of recruitment dynamics. Gray snapper are estuarine dependent and early life stages occur close to shore [31]. Indeed, a close inspection of our projection for gray snapper at the end of the century indicated that above ~31.5° N, gray snapper were excluded from the shallowest areas of the shelf, where winter temperatures were relatively low. The process our model does not capture is that excluding gray snapper from the shallowest parts of the shelf also excludes the adults from the same latitude further offshore [31, 75]. However, our results are surprisingly consistent with Hare et al. [31] when examining nearshore habitat on the shelf, despite our two studies using very different niche modeling approaches.

### Uncertainty in species projections

The magnitude and direction of projected shifts in distribution varied widely among species, but we also found variation in the robustness of projections. We used sixteen different GCMs for species projections so as to examine a range of possible temperature futures within each RCP. While each of these GCMs predict global ocean warming under both greenhouse gas scenarios, they vary significantly in the magnitude of their predictions, particularly at a regional scale [37]. Uncertainty among GCM predictions is higher at the smaller spatial scales at which predictions of living marine resource responses to climate are generally made [22, 36, 37]. Therefore, major differences in species predictions can occur when using different GCMs. Calculating a mean response from multiple projections reduces the bias from any one model and also allows the calculation of variance around projections [22, 23, 31]. However, the amount of variation among GCM projections can be difficult to put into context without comparing across species. Our results for projections of 686 species suggest that results for some species may be unreliable based on poor model agreement. Therefore, there are major benefits in conducting projections with many species under a similar framework, because less robust projections can be down-weighted during interpretation of general trends.

Our method for identifying robust projections was based on two metrics that quantified the distance-directional and latitudinal uncertainty in projected shifts. These two metrics were used, as opposed to using only one, in order to maximize our ability to identify species with greater uncertainty so that regional assemblages could be compared using only robust projections. Further, we did not assess if one metric for uncertainty is more robust than the other. Indeed, some species had different ratings for uncertainty between these two metrics, suggesting that one method to characterize uncertainty may not always be adequate. The uncertainty metrics were essentially based on a ranking of species’ model agreement (i.e., quantiles) and were somewhat arbitrary in that we chose the 95^th^ and 75^th^ percentiles to indicate high and medium uncertainty species, respectively. However, this framework nonetheless provided an objective way to omit less robust projections for analyses of regional trends.

Most of the regional assemblages had at least some unexpected projections (e.g., equatorward shifts in the East Canada region) and our metrics for uncertainty indicated that these were generally less robust. However, we note that our uncertainty methods were potentially conservative, and some species categorized with medium or high uncertainty may still have useful predictions. Further, some regions may have been more likely to have medium or high uncertainty projections based on the coastal geometry and climatology. For example, species originating from the Northeast U.S. were generally shifting into a large area of continental shelf (e.g., Gulf of St. Lawrence and the Grand Banks) and so the potential for variation among GCM predictions was greater there. Similarly, shifts in the Gulf of Mexico were particularly sensitive to the DDU metric (only 38% had low uncertainty for DDU), which probably arises from the lack of a strong along-shore direction in climate velocity, as discussed above. For example, sheepshead (Fig 8) were considered medium uncertainty in the Gulf of Mexico because of the DDU metric, but all 16 GCMs predicted 64% or greater loss in thermal habitat during the 21^st^ century for this species. In addition to considering potential regional biases in our uncertainty metrics, it was also apparent that the level of agreement in projections among the 16 GCMs varied spatially for some species. Thus, even species considered to have high uncertainty projections at the coastwide scale, may still have strong agreement among GCMs within portions of their ranges. This may occur when projections vary more at species range edges than in core areas of distributions. This indicates potential threshold effects [36], where a portion of GCMs predict temperature changes that exceed a limiting value and cause a shift or expansion in the distribution of a species, which leads to greater uncertainty among GCMs. Therefore, projection uncertainty may depend on the spatial scale of interest, and for this study we have focused on coastwide predictions.

Atlantic cod (*Gadus morhua*) are an example of a species with useful projection results at a regional scale, despite not being grouped with low uncertainty projections. This species was generally predicted to occur throughout the north Atlantic, with a southern limit in the Gulf of Maine and Georges Bank during 2007–2020. Atlantic cod was considered medium uncertainty due to relatively high variability in the extent of latitudinal shift among GCMs (i.e., latitudinal uncertainty). Nevertheless, there was strong agreement among the GCMs for a southern range contraction. There was a 90.7% mean (16.1% s.d.) projected loss of thermal habitat for Atlantic cod in U.S. waters, which includes the Gulf of Maine and Georges Bank, during the 21st century under RCP 8.5; thirteen out of the sixteen GCMs that we used projected a greater than 90% loss for this region. Similarly, Kleisner et al. [24] modeled historic and future abundance of Atlantic cod in the Northeast U.S. region based on a climate projection model that had a higher resolution than any of the 16 GCMs in our study [18]. Their results also indicated that thermal habitat in this region will become unsuitable for cod during the 21st century, which was consistent with historical trends of habitat loss [24]. Thus, our medium or high uncertainty species projections may still be useful to resource management depending on the spatial context. Similarly, projected shifts in thermal habitat may differ between coastwide trends, as we reported in our study, and subregions within a species geographic range. For example, Kleisner et al. [4] showed that historic species shifts in latitude and depth can vary among major regions of the continental shelf. Therefore, it is important for resource managers to consider projected changes in thermal habitat at coastwide and regional scales.

Our results incorporated two major sources of uncertainty that may affect future climatic conditions, namely scenario uncertainty of future greenhouse gas concentrations (RCP 2.6 versus RCP 8.5), and model uncertainty among climate projections (i.e., 16 GCMs). We did not include uncertainty from natural internal variability within GCMs—which can affect predicted outcomes of ocean temperatures [36, 37]—because variation among different GCMs was expected to be greater on decadal to centennial timescales [37]. Among the 16 GCMs that we used for projections, the horizontal and vertical resolution varied, along with the extent of spatial coverage on the continental shelf. Further, all of these climate models projected ocean temperature changes at a relatively coarse grain (i.e., 0.25° latitude and longitude or greater), which does not allow for precise representation of mesoscale ocean features (e.g., ocean eddies or upwelling dynamics), complex bathymetry (e.g., deep-water channels), or shallow-coastal areas of the continental shelf [18]. Despite the relatively coarse resolution, IPCC-class global climate models like the ones we used have been widely used to study future changes in upwelling regions, but results have varied. For example, some studies have concluded that the locations of prominent upwelling regions are expected to shift poleward in the 21st century [76], while others predict that the seasonal duration and intensity of upwelling regions will become more homogenous across latitude [77]. Presently, high-resolution (e.g., 10 km horizontal grain) climate projection models that can be run on centennial timescales are rare and present limited opportunities for ensemble modeling [18]. However, as more high resolution GCMs become available, an examination of how climate model resolution impacts the projected response of marine species would be valuable.

Another source of uncertainty that we did not address is associated with species habitat model structure [36]. Previous research has suggested that the structure of habitat models can affect predicted outcomes [69, 78]. Robinson et al. [23] used two types of thermal habitat modeling frameworks for projecting distribution shifts for a group of pelagic fishes. They found differences between the two model types, but uncertainty between model types was generally lower than that attributed to climate projection uncertainties. Parameter uncertainty may also influence predicted outcomes. Hare et al. [31] show that uncertainty in thermal tolerance in gray snapper was the primary source of projection uncertainty. Generally, our niche models performed well at the coast-wide scale in terms of the amount of variation explained (i.e., percent deviance) in the trawl catch data. Further, the presence-absence models had high predictive power when applied to independent testing data (median AUC: 0.93). Nevertheless, a modeling structure that, for example, accounted for individual vessel effects [59] or spatial autocorrelation [79] might refine parameter values. There is still a relatively poor understanding for how these habitat model uncertainties might affect projections and how these uncertainties vary among species [36]. Future work will be devoted to quantifying habitat model uncertainties with a subset of the species analyzed here.

### Implications for the management of living marine resources

Our results contribute to a growing body of work that stresses the importance of the level of global warming for the magnitude of changes in living marine resources by the end of this century. We found dramatic differences in the magnitude of distribution and thermal biomass changes between RCP 2.6 and 8.5. These major difference result from only about 2 to 3°C global warming difference [17]. Marine species responses to temperature are often nonlinear, and so small increases in temperature can have large impacts on predicted outcomes [31, 68, 80]. A high greenhouse gas emissions scenario has been predicted to have large impacts on regional biodiversity [34] and a net loss in fisheries productivity in most coastal regions of North America [81]. Our results add to this and suggest that a future under a ‘business-as-usual’ greenhouse gas emissions scenario (i.e., RCP 8.5) will lead to large shifts in the distribution of species important to fisheries. These shifts in turn may lead to a host of management challenges, including shifts in fishing locations [82], conflict over regional allocation of fisheries quota, displaced fisherman, and changes in stock boundaries [83, 84]. However, if emissions are curtailed to a level that is consistent with the Paris Agreement (i.e., RCP 2.6), then dramatic shifts in species distribution can be mostly avoided.

A primary motivation for producing these species projections was to contribute to a set of tools available to policy makers and managers considering climate adaptation of marine fisheries management. In the United States, fisheries are managed regionally, including species that are managed by individual states and federally managed fisheries that are governed by regional councils with representatives from neighboring states. Existing tools for climate adaptation in fisheries management include descriptions of historical species distribution shifts from biological survey data [3], expert judgment climate vulnerability assessments for marine species [20], and social vulnerability assessments [85]. The projections produced here can also help regional managers identify species that are most likely to experience major changes in availability to fisheries. At the regional management scale, there will be fisheries that experience negative consequences of ocean warming, but also potentially positive outcomes when valuable species expand into an area [24]. As the availability of species shift, coordination among regional management groups will be critically important, and the projections summarized here may offer an objective tool for management groups to begin communication prior to conflict over, for example, regional allocation [84]. Further, these projections can be utilized for future efforts to assess the risk of shifting fisheries for different coastal communities [85].

## Supporting information

S1 Appendix List of projected species. The proportion of deviance explained for each GAM habitat model (presence-absence and biomass) along with the geographic region used to group each species, the level of projection uncertainty, and the mean and standard deviation of projections in both distance shifted (km) and change in habitat availability (%) over the course of the 21^st^ century for both RCP 2.6 and 8.5.
(PDF)
