# Supplementary material for: Projecting shifts in thermal habitat for 686 species on the North American continental shelf
Source: PLoS One. 2018 May 16;13(5):e0196127. doi: 10.1371/journal.pone.0196127 (PMC5955691; doi:10.1371/journal.pone.0196127)
Supplement: S1 Appendix — The proportion of deviance explained for each GAM habitat model (presence-absence and biomass) along with the geographic region used to group each species, the level of projection uncertainty, and the mean and standard deviation of projections in both distance shifted (km) and change in habitat availability (%) over the course of the 21st century for both RCP 2.6 and 8.5. (PDF) [file pone.0196127.s001.pdf]

Species	devPA	devBiom	Region	RCP	Uncert.	Shift	sd_shift	%Hab.	sd_hab
acanthephyra pelagica	0.68	0.90	E. Canada	26	medium	164.7	124.6	13.1	20.3
				85	low	437.6	150.2	10.6	42.7
			G. Mexico	26	low	297.5	147.9	-77.6	14.5
				85	low	401.7	192.1	-98.0	1.1
acanthostracion quadricornis	0.48	0.97	SE U.S.	26	low	53.7	44.6	149.0	128.1
				85	low	148.1	87.8	2006.8	1266.9
			G. Mexico	26	low	19.0	17.3	24.1	20.7
				85	low	55.9	27.1	127.7	47.9
achelous spinicarpus	0.53	0.89	G. Mexico	26	high	18.0	39.4	20.2	52.8
				85	high	52.9	116.6	-64.4	36.5
achelous spinimanus	0.37	0.96	SE U.S.	26	medium	17.3	9.4	61.7	52.5
				85	low	46.4	22.0	160.9	124.1
			G. Mexico	26	low	21.9	11.6	15.3	10.7
				85	medium	39.3	17.3	27.6	15.1
actinauge verrilli	0.35	0.15	G. Alaska	26	low	136.9	78.4	-1.8	5.7
				85	low	383.9	139.8	0.7	11.9
aforia circinata	0.42	0.97	E. Bering S.	26	medium	53.7	36.5	-36.4	56.7
				85	low	139.7	39.2	-96.5	9.1
albatrossia pectoralis	0.54	0.92	G. Alaska	26	low	365.8	278.9	15.7	26.9
				85	low	1184.3	302.9	147.1	82.5
alectis ciliaris	0.24	0.94	SE U.S.	26	low	8.0	5.1	26.3	17.1
				85	medium	32.6	35.4	137.9	53.7
			G. Mexico	26	low	17.0	12.4	29.0	19.4
				85	medium	34.4	16.0	109.8	36.4
alepocephalus bairdii	0.49	0.88	E. Canada	26	medium	157.0	140.0	5.6	24.0
				85	low	494.3	198.4	21.7	45.4
alepocephalus tenebrosus	0.71	0.97	G. Alaska	26	high	299.5	280.8	5.3	34.0
				85	medium	1417.7	567.6	268.9	172.0
allosmerus elongatus	0.32	0.89	G. Alaska	26	low	384.2	292.8	39.7	29.0
				85	low	1491.7	379.5	226.7	125.5
alosa aestivalis	0.41	0.78	NE U.S.	26	high	204.7	171.2	-2.3	55.9
				85	high	400.2	208.1	-66.5	25.3
alosa pseudoharengus	0.37	0.78	NE U.S.	26	high	149.9	141.6	-24.9	24.9
				85	medium	660.0	272.3	-71.5	21.1
alosa sapidissima	0.27	0.79	NE U.S.	26	low	65.0	50.9	20.6	28.6
				85	low	337.6	188.0	-19.9	57.5
	0.43	0.90	West U.S.	26	low	332.6	246.4	34.8	35.0
				85	low	1600.7	381.0	151.7	106.9

Species	devPA	devBiom	Region	RCP	Uncert.	Shift	sd_shift	%Hab.	sd_hab
aluterus schoepfii	0.34	0.94	SE U.S.	26	medium	17.1	10.8	84.1	70.4
				85	medium	41.1	26.4	1032.8	537.1
			G. Mexico	26	high	11.1	9.7	24.1	24.5
				85	medium	23.4	24.4	143.4	59.9
amblyraja radiata	0.40	0.87	E. Canada	26	low	118.2	70.4	5.5	11.6
				85	medium	442.1	244.0	-15.5	35.6
ammodytes dubius	0.35	0.76	E. Canada	26	medium	92.8	59.3	-23.0	15.5
				85	high	256.4	135.8	-64.9	24.3
ammodytes hexapterus	0.23	0.37	E. Bering S.	26	low	22.1	18.1	2.2	18.4
				85	medium	69.7	34.8	-22.9	52.5
ampheraster marianus	0.43	0.95	G. Alaska	26	medium	237.3	202.4	2.6	17.8
				85	medium	1273.6	417.8	76.8	49.3
amusium papyraceum	0.56	0.92	G. Mexico	26	medium	0.2	0.3	130.8	127.7
				85	medium	0.8	1.6	714.2	704.3
anadara baughmani	0.49	0.93	G. Mexico	26	low	36.4	26.4	30.3	33.5
				85	medium	51.0	34.2	120.9	71.5
anarhichas denticulatus	0.45	0.86	E. Canada	26	low	133.9	114.6	2.4	12.3
				85	low	456.2	150.1	-32.0	41.2
anarhichas lupus	0.29	0.89	E. Canada	26	low	124.8	65.4	3.4	9.8
				85	medium	432.4	245.3	-24.0	35.5
anarhichas minor	0.44	0.85	E. Canada	26	low	71.3	49.3	-18.4	32.8
				85	low	202.7	105.2	-66.3	42.6
anasimus latus	0.47	0.91	G. Mexico	26	medium	0.4	0.5	82.9	84.4
				85	low	1.9	2.7	153.6	182.5
anchoa hepsetus	0.40	0.91	SE U.S.	26	medium	15.4	16.7	4.7	13.7
				85	high	159.1	173.8	41.0	42.0
			G. Mexico	26	medium	19.6	14.3	-8.2	10.2
				85	medium	61.2	42.2	-64.1	18.9
anchoa lyolepis	0.26	0.95	SE U.S.	26	low	16.2	14.9	7.5	11.0
				85	low	91.2	37.8	-0.3	19.9
			G. Mexico	26	low	24.0	17.7	-12.6	9.3
				85	low	82.2	30.9	-46.2	9.8
anchoa mitchilli	0.32	0.84	SE U.S.	26	high	68.8	91.8	0.3	25.2
				85	low	589.3	226.9	150.5	134.1
			G. Mexico	26	medium	24.0	24.3	-24.5	12.8
				85	high	53.3	43.5	-77.3	7.9

Species	devPA	devBiom	Region	RCP	Uncert.	Shift	sd_shift	%Hab.	sd_hab
ancylopsetta dilecta	0.49	0.91	SE U.S.	26	medium	77.8	96.4	97.9	117.2
				85	low	321.9	137.1	621.0	1508.7
			G. Mexico	26	medium	68.8	68.7	-7.0	23.3
				85	low	254.5	150.9	-74.5	24.6
ancylopsetta ommata	0.41	0.98	SE U.S.	26	low	9.1	7.7	23.6	27.6
				85	low	57.4	38.7	66.0	40.2
			G. Mexico	26	low	8.0	4.2	1.2	3.2
				85	high	16.7	10.1	-5.4	9.2
anoplopoma fimbria	0.46	0.24	G. Alaska	26	low	181.9	116.1	0.2	4.6
				85	low	689.2	105.9	-10.9	14.9
anthoptilum grandiflorum	0.42	0.98	G. Alaska	26	medium	249.6	194.2	5.9	31.9
				85	medium	1463.4	414.8	77.7	85.1
antimora microlepis	0.57	0.92	West U.S.	26	low	262.4	170.8	159.3	81.3
				85	high	1022.4	865.5	2040.3	4826.3
antimora rostrata	0.74	0.91	E. Canada	26	medium	185.1	139.3	-0.8	16.3
				85	low	526.1	189.3	5.3	40.8
aphrocallistes vastus	0.27	0.69	E. Bering S.	26	medium	271.0	177.6	17.3	36.5
				85	low	709.1	355.2	-16.7	110.0
aphrodita negligens	0.19	0.78	E. Bering S.	26	high	48.1	64.0	-22.0	31.3
				85	medium	128.0	122.1	-86.0	17.4
apostichopus leukothele	0.34	0.92	G. Alaska	26	low	290.3	168.2	14.8	16.3
				85	low	888.4	229.7	16.3	51.6
apristurus brunneus	0.59	0.97	G. Alaska	26	high	228.7	196.1	-5.2	9.6
				85	high	924.4	370.9	24.6	25.5
apristurus profundorum	0.50	0.92	E. Canada	26	low	159.7	103.8	-2.9	19.4
				85	low	324.0	139.1	24.5	35.4
aptocyclus ventricosus	0.16	0.87	E. Bering S.	26	low	79.2	55.9	-28.3	26.6
				85	low	225.2	69.0	-83.9	24.0
archosargus probatocephalus	0.32	0.97	SE U.S.	26	low	34.5	25.9	-13.8	11.0
				85	low	333.8	170.8	-46.4	16.9
			G. Mexico	26	medium	23.5	18.3	-24.4	15.5
				85	medium	49.2	25.6	-82.7	7.3
arctomelon stearnsii	0.23	0.83	E. Bering S.	26	low	139.5	107.0	-34.2	48.2
				85	low	528.2	131.9	-93.9	12.1
arctozenus risso	0.44	0.84	E. Canada	26	medium	303.3	216.2	23.9	76.6
				85	medium	672.1	278.9	52.4	69.8

Species	devPA	devBiom	Region	RCP	Uncert.	Shift	sd_shift	%Hab.	sd_hab
arenaeus cribrarius	0.48	0.95	SE U.S.	26	medium	19.6	15.8	28.0	42.3
				85	medium	60.7	35.5	7.7	101.4
			G. Mexico	26	medium	31.3	19.6	7.8	18.7
				85	medium	82.4	60.1	-37.8	19.6
argentina sialis	0.32	0.95	West U.S.	26	low	167.0	124.1	42.9	36.8
				85	low	791.2	259.1	257.0	176.8
argentina silus	0.35	0.75	E. Canada	26	low	118.6	92.8	24.2	33.9
				85	low	491.3	205.7	25.2	123.1
argis dentata	0.55	0.85	E. Canada	26	low	79.2	66.7	-28.7	32.6
				85	medium	199.0	116.7	-79.9	28.6
	0.16	0.95	E. Bering S.	26	low	32.5	15.3	-46.2	46.7
				85	low	86.5	42.5	-98.1	3.9
argis lar	0.19	0.96	E. Bering S.	26	low	40.4	29.3	-41.5	34.1
				85	low	117.4	49.9	-95.7	7.4
argopecten gibbus	0.31	0.86	SE U.S.	26	medium	87.0	93.4	243.3	294.1
				85	medium	401.8	207.6	5218.8	5224.3
			G. Mexico	26	medium	37.6	40.5	-0.3	29.7
				85	medium	221.2	146.9	-82.3	24.2
ariopsis felis	0.43	0.98	SE U.S.	26	low	29.9	23.7	84.5	102.1
				85	low	90.3	57.1	484.8	635.6
			G. Mexico	26	high	31.3	46.9	-0.5	13.9
				85	medium	95.8	99.5	-57.1	24.0
artediellus uncinatus	0.29	0.74	E. Canada	26	medium	202.1	195.5	-14.1	42.0
				85	high	621.4	472.5	-57.9	28.5
aspidophoroides bartoni	0.26	0.95	E. Bering S.	26	medium	89.5	69.1	-33.5	65.6
				85	low	184.1	87.1	-97.5	5.6
aspidophoroides monopterygius	0.30	0.83	E. Canada	26	low	127.8	84.4	-9.4	26.8
				85	low	261.9	143.3	-67.7	32.5
aspidophoroides olrikii	0.49	0.89	E. Canada	26	low	120.7	79.0	-35.6	39.2
				85	medium	249.5	107.2	-80.4	32.3
asterias amurensis	0.70	0.94	E. Bering S.	26	medium	98.1	73.9	-2.0	148.1
				85	low	200.8	115.4	-97.9	4.2
astropecten cingulatus	0.46	0.92	G. Mexico	26	high	36.2	25.6	45.7	46.0
				85	medium	51.9	57.2	297.0	168.7
astropecten duplicatus	0.40	0.90	G. Mexico	26	medium	81.1	85.6	62.0	63.3
				85	medium	312.2	199.1	15.6	98.8
atheresthes stomias	0.58	0.89	G. Alaska	26	low	401.3	318.0	9.3	28.3
				85	low	1522.8	341.0	15.7	60.3

**Species**	**devPA**	**devBiom**	**Region**	**RCP**	**Uncert.**	**Shift**	**sd_shift**	**%Hab.**	**sd_hab**
atlantopandalus propinqvus	0.41	0.83	E. Canada	26	medium	91.1	52.9	22.9	24.1
				85	medium	339.1	254.6	15.3	42.6
bagre marinus	0.35	0.98	SE U.S.	26	medium	82.9	86.0	515.7	437.2
				85	high	145.9	93.1	13751.7	17110.7
			G. Mexico	26	high	49.2	53.9	439.4	476.4
				85	high	136.7	124.6	30123.2	49134.1
bairdiella chrysoura	0.42	0.93	SE U.S.	26	low	29.7	24.0	-11.8	10.0
				85	medium	434.7	328.9	2.6	56.6
			G. Mexico	26	low	16.8	9.0	-7.8	5.8
				85	medium	30.9	16.4	-22.5	7.8
balistes capriscus	0.36	0.93	SE U.S.	26	medium	21.4	14.4	50.8	44.8
				85	low	74.2	47.5	503.0	160.3
			G. Mexico	26	low	39.8	34.9	13.4	20.2
				85	low	183.8	91.6	-28.1	48.0
bathyagonus nigripinnis	0.28	0.15	G. Alaska	26	low	161.0	109.3	-3.4	7.8
				85	low	739.3	258.6	-14.5	19.4
bathybembix bairdii	0.60	0.96	G. Alaska	26	medium	327.3	342.3	3.0	28.0
				85	medium	1412.6	514.7	215.7	129.7
bathylagus euryops	0.68	0.91	E. Canada	26	low	131.5	74.2	6.5	13.4
				85	medium	232.4	114.4	4.9	29.3
bathymaster signatus	0.24	0.03	E. Bering S.	26	low	104.6	59.3	-2.9	6.1
				85	low	276.0	83.1	-24.0	12.1
bathypolypus arcticus	0.38	0.75	NE U.S.	26	low	158.9	135.1	27.0	61.2
				85	low	728.5	268.9	-30.1	57.9
bathyraja spinicauda	0.45	0.89	E. Canada	26	medium	153.9	131.6	3.9	16.8
				85	low	556.2	195.5	-12.1	42.0
bellator militaris	0.38	0.87	SE U.S.	26	high	61.4	107.9	297.6	403.5
				85	low	397.4	172.2	3129.0	3739.5
			G. Mexico	26	medium	55.8	67.9	3.6	29.3
				85	medium	227.9	150.5	-66.9	48.7
beringius behringi	0.28	0.97	E. Bering S.	26	medium	72.0	44.7	-38.6	60.8
				85	medium	148.8	74.9	-98.0	4.3
berryteuthis magister	0.37	0.10	G. Alaska	26	low	110.3	62.6	-2.6	4.6
				85	low	353.5	115.6	-5.5	12.9
bollmannia communis	0.38	0.91	G. Mexico	26	low	43.7	37.9	-1.9	17.0
				85	medium	133.4	79.9	-72.9	24.1
boltenia ovifera	0.46	0.95	E. Bering S.	26	medium	102.3	75.2	-4.8	148.6
				85	high	282.7	208.9	-99.4	1.3

Species	devPA	devBiom	Region	RCP	Uncert.	Shift	sd_shift	%Hab.	sd_hab
boreogadus saida	0.69	0.88	E. Canada	26	medium	71.6	63.0	-30.4	38.0
				85	medium	154.2	85.0	-82.6	22.9
	0.61	0.96	E. Bering S.	26	medium	19.8	15.8	-51.0	40.1
				85	medium	73.7	72.2	-98.4	2.8
bothrocara brunneum	0.60	0.94	G. Alaska	26	medium	323.0	297.1	0.4	19.1
				85	medium	1514.7	386.4	72.1	48.4
bregmaceros atlanticus	0.32	0.89	G. Mexico	26	low	48.3	43.5	25.4	31.1
				85	low	156.1	84.7	-32.3	52.5
brevoortia patronus	0.36	0.89	G. Mexico	26	high	54.5	51.3	183.1	202.4
				85	high	107.5	131.4	2513.7	1865.5
brevoortia tyrannus	0.32	0.82	SE U.S.	26	medium	128.7	156.1	-5.5	15.6
				85	low	838.6	280.7	199.2	268.3
brosme brosme	0.37	0.89	NE U.S.	26	low	147.5	97.0	25.0	47.8
				85	medium	674.4	295.0	1.9	90.2
brotula barbata	0.39	0.90	G. Mexico	26	high	4.1	6.1	-29.7	30.5
				85	high	20.1	32.4	-95.3	7.3
buccinum angulosum	0.45	0.93	E. Bering S.	26	low	41.1	27.2	-43.1	36.5
				85	low	181.3	78.2	-96.7	6.0
buccinum plectrum	0.22	0.93	E. Bering S.	26	low	39.7	23.9	-47.5	38.7
				85	low	156.5	79.3	-97.2	5.6
buccinum polare	0.41	0.94	E. Bering S.	26	low	52.6	38.8	-37.7	36.9
				85	low	206.6	79.5	-94.5	9.8
buccinum scalariforme	0.37	0.95	E. Bering S.	26	low	50.1	35.3	-41.8	42.0
				85	low	158.2	66.4	-94.6	11.6
calamus leucosteus	0.32	0.92	SE U.S.	26	medium	35.3	29.4	-7.2	27.9
				85	high	179.0	171.3	-55.6	17.1
			G. Mexico	26	low	56.4	50.0	-22.8	13.5
				85	medium	176.9	136.2	-21.1	23.1
calamus proridens	0.59	0.91	SE U.S.	26	high	19.9	36.7	1394.4	2563.2
				85	medium	262.8	212.3	15545.2	21327.5
			G. Mexico	26	high	31.3	34.3	67.9	92.8
				85	high	83.1	64.2	126.8	218.5
calappa flammea	0.31	0.97	SE U.S.	26	high	11.1	13.4	8.3	10.5
				85	high	85.4	107.3	36.5	31.9
			G. Mexico	26	medium	10.8	7.0	-9.2	9.4
				85	medium	49.4	27.5	-32.2	19.1

Species	devPA	devBiom	Region	RCP	Uncert.	Shift	sd_shift	%Hab.	sd_hab
calappa sulcata	0.41	0.98	SE U.S.	26	low	12.9	12.3	10.5	6.9
				85	high	125.6	160.9	53.4	30.3
			G. Mexico	26	medium	4.6	2.9	5.4	3.9
				85	high	8.7	6.5	17.4	9.0
callinectes ornatus	0.46	0.95	SE U.S.	26	low	13.3	10.3	46.5	40.8
				85	high	34.1	22.9	155.5	97.5
			G. Mexico	26	low	47.2	44.5	34.0	23.9
				85	low	151.2	77.9	251.3	130.3
callinectes sapidus	0.31	0.96	SE U.S.	26	low	25.7	20.3	-10.0	10.1
				85	high	263.6	262.6	21.7	54.2
			G. Mexico	26	low	10.5	5.7	6.8	9.4
				85	high	43.1	22.3	0.1	21.3
callinectes similis	0.60	0.94	SE U.S.	26	medium	30.2	32.4	108.7	106.9
				85	low	78.0	44.5	709.2	525.9
			G. Mexico	26	medium	15.2	19.0	8.3	9.3
				85	medium	29.2	24.0	-18.6	25.9
cancer borealis	0.24	0.80	E. Canada	26	high	272.6	248.1	0.2	26.4
				85	medium	709.3	354.2	-43.4	46.0
cancer irroratus	0.30	0.72	E. Canada	26	high	364.3	517.3	-30.8	55.0
				85	high	891.8	586.1	-80.9	26.5
cancer productus	0.35	0.97	West U.S.	26	low	262.9	180.9	36.9	29.9
				85	low	1377.6	401.6	121.5	106.2
caranx crysos	0.32	0.94	SE U.S.	26	low	11.3	8.3	18.1	16.6
				85	medium	132.2	132.6	170.4	88.5
			G. Mexico	26	high	24.2	18.3	24.6	20.7
				85	medium	64.2	73.8	20.5	41.2
caranx hippos	0.26	0.95	SE U.S.	26	low	33.3	22.5	27.7	20.0
				85	high	313.8	352.1	247.1	110.0
			G. Mexico	26	medium	14.1	10.6	21.8	14.3
				85	medium	22.7	16.2	121.8	34.2
carcharhinus acronotus	0.30	0.97	SE U.S.	26	low	41.6	37.8	25.5	17.0
				85	medium	168.4	101.5	231.6	99.6
			G. Mexico	26	low	21.0	17.6	10.0	11.5
				85	low	64.4	38.2	90.6	31.7
carcharhinus plumbeus	0.35	0.96	SE U.S.	26	medium	67.6	79.9	-0.6	9.0
				85	low	753.6	331.1	14.9	24.9
			G. Mexico	26	medium	31.7	25.1	-31.7	19.7
				85	medium	48.9	41.0	-91.4	4.1

Species	devPA	devBiom	Region	RCP	Uncert.	Shift	sd_shift	%Hab.	sd_hab
careproctus melanurus	0.44	0.93	G. Alaska	26	medium	325.2	300.1	3.7	11.6
				85	medium	1437.2	356.3	33.9	42.1
careproctus rastrinus	0.34	0.93	E. Bering S.	26	low	31.2	27.3	-27.6	27.4
				85	low	127.5	62.1	-83.2	22.6
caretta caretta	0.30	0.98	SE U.S.	26	low	13.8	10.4	11.3	15.2
				85	medium	137.6	101.7	100.4	47.3
			G. Mexico	26	medium	13.8	15.2	0.4	4.9
				85	low	39.1	26.9	-23.6	13.6
caulolatilus intermedius	0.47	0.91	G. Mexico	26	high	62.3	57.1	-15.2	21.6
				85	low	211.2	125.3	-82.1	16.5
centropristis ocyurus	0.43	0.93	SE U.S.	26	low	105.9	94.2	395.2	524.2
				85	medium	373.1	204.8	7522.1	8960.7
			G. Mexico	26	medium	30.2	35.2	7.4	29.7
				85	medium	134.8	92.8	-65.4	32.0
centropristis philadelphica	0.54	0.96	SE U.S.	26	low	19.3	15.8	49.7	37.8
				85	low	83.1	48.1	225.4	137.2
			G. Mexico	26	medium	25.7	25.8	2.9	6.8
				85	low	87.1	43.2	-28.4	16.9
centropristis striata	0.33	0.80	SE U.S.	26	medium	65.8	69.1	-2.9	16.7
				85	low	528.6	139.6	82.5	58.7
			G. Mexico	26	medium	54.4	43.0	-41.2	21.0
				85	medium	116.3	95.2	-93.4	7.2
centroscyllium fabricii	0.49	0.91	E. Canada	26	low	154.3	105.9	11.9	20.9
				85	low	392.0	132.5	45.8	40.1
ceramaster japonicus	0.17	0.80	E. Bering S.	26	low	113.6	60.2	-33.6	55.7
				85	low	412.7	119.0	-92.0	14.4
ceramaster patagonicus	0.21	0.83	E. Bering S.	26	low	122.2	102.2	-34.3	54.1
				85	low	275.3	131.3	-94.1	16.5
chaceon quinquedens	0.33	0.81	NE U.S.	26	high	46.2	46.0	13.1	22.1
				85	medium	188.3	181.5	-10.6	36.6
chaetodipterus faber	0.40	0.97	SE U.S.	26	medium	11.7	8.4	45.3	37.2
				85	low	31.8	21.4	329.2	150.4
			G. Mexico	26	high	21.5	14.1	53.9	46.5
				85	medium	42.0	35.5	347.8	170.2
chauliodus macouni	0.38	0.92	G. Alaska	26	high	277.4	215.6	12.4	26.0
				85	high	992.5	402.7	167.8	82.7

Species	devPA	devBiom	Region	RCP	Uncert.	Shift	sd_shift	%Hab.	sd_hab
chauliodus sloani	0.64	0.89	E. Canada	26	medium	163.2	116.4	11.5	21.8
				85	low	444.0	140.4	28.3	47.2
			G. Mexico	26	low	49.4	36.5	-38.2	18.8
				85	medium	68.6	47.6	-93.2	3.6
cheiraster dawsoni	0.23	0.18	E. Bering S.	26	low	112.8	70.5	-2.6	6.4
				85	low	303.1	137.9	0.4	19.3
chiasmodon niger	0.59	0.91	E. Canada	26	medium	182.3	117.0	1.8	16.8
				85	low	492.0	173.3	7.2	41.1
chilara taylori	0.30	0.92	West U.S.	26	medium	192.2	178.0	20.2	16.2
				85	medium	836.6	439.9	84.8	121.2
chilomycterus schoepfii	0.38	0.96	SE U.S.	26	low	27.1	22.7	-9.6	11.0
				85	medium	382.6	231.5	-14.0	40.4
			G. Mexico	26	medium	7.9	6.5	-3.7	5.8
				85	medium	24.2	13.3	-27.6	12.0
chionoecetes bairdi	0.47	0.31	G. Alaska	26	low	117.7	84.2	18.2	19.8
				85	low	354.9	154.6	149.5	87.2
chionoecetes opilio	0.62	0.93	E. Canada	26	low	96.4	77.4	-18.0	30.0
				85	low	323.5	111.9	-45.6	56.5
	0.76	0.94	E. Bering S.	26	low	52.7	36.4	-44.2	44.2
				85	low	135.3	61.2	-96.3	8.7
chionoecetes tanneri	0.63	0.91	G. Alaska	26	medium	527.3	455.7	20.2	39.7
				85	high	1833.8	437.9	187.0	116.4
chirona evermanni	0.12	0.86	E. Bering S.	26	low	78.8	49.2	-39.5	36.6
				85	low	211.5	100.5	-93.2	12.7
chlamys islandica	0.40	0.87	E. Canada	26	low	129.7	65.2	-15.5	34.3
				85	medium	229.2	129.2	-73.4	32.1
chlamys rubida	0.24	0.84	E. Bering S.	26	low	102.3	70.5	-14.3	35.3
				85	low	352.4	100.6	-72.6	44.8
chlorophthalmus agassizi	0.46	0.77	NE U.S.	26	low	183.3	127.2	34.5	31.5
				85	low	601.3	246.6	207.5	106.5
chloroscombrus chrysurus	0.57	0.94	SE U.S.	26	medium	32.3	37.8	84.1	84.2
				85	low	86.4	60.3	632.1	518.4
			G. Mexico	26	low	19.6	12.0	2.2	7.0
				85	medium	58.5	24.4	18.8	15.8
chrysaora melanaster	0.22	0.18	G. Alaska	26	low	156.7	144.7	21.3	19.3
				85	low	656.9	216.9	74.8	33.2
chrysaora quinquecirrha	0.33	0.87	G. Mexico	26	medium	66.6	52.6	87.4	100.2
				85	medium	268.8	193.6	4.8	155.9

Species	devPA	devBiom	Region	RCP	Uncert.	Shift	sd_shift	%Hab.	sd_hab
ciliatocardium ciliatum	0.19	0.96	E. Bering S.	26	medium	41.8	26.0	-46.9	48.9
				85	medium	110.0	55.7	-98.9	2.5
citharichthys arctifrons	0.34	0.70	NE U.S.	26	low	56.4	47.3	44.1	43.4
				85	medium	285.7	234.6	164.8	101.6
			G. Mexico	26	medium	103.3	97.1	-65.0	24.0
				85	high	151.5	102.2	-99.8	0.2
citharichthys macrops	0.52	0.96	SE U.S.	26	low	9.7	9.3	5.3	7.1
				85	low	63.9	53.2	16.1	23.0
			G. Mexico	26	low	15.3	12.4	-8.6	9.3
				85	medium	47.3	40.4	-36.9	15.2
citharichthys sordidus	0.66	0.93	West U.S.	26	low	236.2	165.0	37.7	29.1
				85	low	1147.4	276.6	217.3	76.1
citharichthys spilopterus	0.36	0.97	SE U.S.	26	low	5.8	3.2	28.1	21.3
				85	medium	32.5	31.7	154.3	43.3
			G. Mexico	26	medium	6.6	3.1	11.5	7.9
				85	medium	11.7	6.7	46.0	10.4
clathrina blanca	0.39	0.96	E. Bering S.	26	high	160.0	130.6	-33.0	79.6
				85	medium	290.3	100.6	-98.4	3.7
clinopegma magnum	0.43	0.92	E. Bering S.	26	medium	35.6	23.6	-31.9	44.9
				85	low	136.9	60.4	-93.9	10.2
clupea harengus	0.30	0.82	E. Canada	26	low	94.3	64.6	1.7	17.1
				85	low	380.0	144.0	-12.6	31.0
clupea pallasii	0.25	0.08	G. Alaska	26	low	203.1	183.1	25.1	22.3
				85	low	784.2	216.8	118.3	52.6
conger oceanicus	0.22	0.73	SE U.S.	26	medium	174.4	169.7	15.6	25.8
				85	low	906.0	303.5	242.4	161.9
			G. Mexico	26	low	50.0	25.2	-13.3	7.1
				85	low	98.3	32.8	-39.6	10.0
coryphaenoides acrolepis	0.67	0.91	G. Alaska	26	medium	365.5	349.0	9.6	35.7
				85	low	1497.0	495.5	309.5	214.6
coryphaenoides cinereus	0.44	0.84	E. Bering S.	26	high	528.8	659.8	26.9	36.4
				85	medium	2738.7	1198.8	619.6	706.6
coryphaenoides rupestris	0.69	0.89	E. Canada	26	medium	152.8	102.7	-1.4	11.8
				85	low	444.6	166.9	-17.6	37.0
cottunculus microps	0.43	0.89	E. Canada	26	low	107.6	82.9	0.8	13.1
				85	low	301.1	120.4	-42.1	34.0
crangon dalli	0.19	0.96	E. Bering S.	26	medium	51.2	40.1	-35.6	45.9
				85	low	124.3	47.3	-95.4	7.9

Species	devPA	devBiom	Region	RCP	Uncert.	Shift	sd_shift	%Hab.	sd_hab
crangon septemspinosa	0.22	0.75	NE U.S.	26	medium	154.6	153.9	-14.2	17.0
				85	medium	760.0	386.3	-56.5	18.0
cross papposus	0.32	0.80	E. Canada	26	low	194.7	112.7	-1.7	25.4
				85	low	365.3	175.8	-62.3	35.6
crossaster borealis	0.43	0.15	E. Bering S.	26	low	50.4	32.3	-16.5	9.9
				85	low	152.1	57.4	-48.5	8.4
crossaster papposus	0.31	0.82	E. Canada	26	high	253.9	269.3	2.1	120.5
				85	high	611.2	459.2	-54.1	55.5
cryptacanthodes maculatus	0.27	0.78	NE U.S.	26	medium	204.8	183.4	5.4	40.6
				85	high	530.4	364.6	-33.4	110.3
cryptonatica russa	0.27	0.92	E. Bering S.	26	low	16.1	9.6	-41.5	35.6
				85	low	60.8	31.9	-96.1	6.6
ctenodiscus crispatus	0.21	0.83	E. Canada	26	medium	130.1	118.6	-1.9	14.3
				85	low	620.7	295.6	-29.5	35.4
	0.33	0.43	E. Bering S.	26	high	48.3	75.0	-44.5	29.9
				85	medium	235.4	170.0	-94.5	5.2
cucumaria fallax	0.24	0.94	E. Bering S.	26	low	95.6	86.5	-23.4	75.9
				85	low	256.8	78.4	-96.4	7.1
cucumaria frondosa	0.26	0.83	E. Canada	26	low	161.9	85.7	1.9	21.1
				85	medium	504.3	300.4	-34.1	73.3
cyanea capillata	0.24	0.13	G. Alaska	26	low	138.4	120.8	12.2	12.3
				85	medium	414.1	236.5	17.4	45.6
cyclopsetta chittendeni	0.47	0.91	G. Mexico	26	medium	3.1	4.0	40.5	50.5
				85	medium	6.6	7.1	19.3	88.8
cyclopsetta fimbriata	0.36	0.97	SE U.S.	26	medium	55.7	70.2	307.5	331.1
				85	low	301.0	141.6	8018.5	8177.8
			G. Mexico	26	medium	28.5	30.6	29.5	37.4
				85	low	155.4	92.3	-21.9	64.4
cyclopterus lumpus	0.29	0.87	E. Canada	26	low	83.2	61.3	-18.8	67.6
				85	low	213.0	130.7	-67.2	73.0
cymatogaster aggregata	0.39	0.95	West U.S.	26	low	145.7	105.8	67.0	55.4
				85	low	754.9	301.3	474.5	390.3
cynoscion nothus	0.57	0.87	SE U.S.	26	medium	23.1	23.4	-2.7	21.2
				85	medium	475.4	377.6	-33.1	72.5
cynoscion regalis	0.50	0.87	SE U.S.	26	high	141.0	179.4	-20.3	19.0
				85	low	826.5	266.1	384.0	597.6

Species	devPA	devBiom	Region	RCP	Uncert.	Shift	sd_shift	%Hab.	sd_hab
dasyatis americana	0.31	0.98	SE U.S.	26	low	19.9	16.5	-0.7	9.6
				85	medium	326.5	221.8	31.0	53.3
			G. Mexico	26	medium	46.1	51.6	-20.0	16.1
				85	medium	170.5	113.4	-87.9	14.1
dasyatis centroura	0.32	0.96	SE U.S.	26	low	12.8	10.4	6.4	10.1
				85	medium	347.3	242.8	2.7	34.8
			G. Mexico	26	medium	44.4	28.3	-20.5	18.7
				85	medium	132.3	55.3	-85.1	12.5
dasyatis sabina	0.37	0.98	SE U.S.	26	low	27.2	17.7	-7.2	12.3
				85	high	561.4	482.7	17.1	66.7
			G. Mexico	26	medium	18.5	17.0	-6.5	8.4
				85	medium	56.0	45.8	-59.8	12.6
dasyatis say	0.49	0.97	SE U.S.	26	low	26.0	20.1	-5.0	8.5
				85	medium	454.2	254.4	-3.6	36.6
			G. Mexico	26	low	41.7	36.5	-19.3	15.9
				85	medium	122.8	50.6	-84.0	13.2
dasycottus setiger	0.28	0.13	G. Alaska	26	low	122.8	80.2	10.1	8.9
				85	low	419.1	129.5	46.3	19.5
decapterus macarellus	0.31	0.73	SE U.S.	26	medium	9.5	6.2	22.8	18.2
				85	medium	33.0	34.3	222.8	123.5
			G. Mexico	26	high	21.4	18.7	35.9	27.0
				85	medium	42.6	29.1	302.1	104.8
decapterus punctatus	0.28	0.87	SE U.S.	26	low	13.6	13.0	2.0	9.9
				85	low	150.5	101.5	14.7	36.6
			G. Mexico	26	high	15.4	13.2	-20.7	12.3
				85	high	46.5	32.0	-70.5	11.8
dichelopandalus leptocerus	0.37	0.71	NE U.S.	26	low	30.7	25.0	25.1	45.9
				85	medium	327.0	238.6	-56.7	59.8
diplectrum bivittatum	0.53	0.97	SE U.S.	26	high	46.5	41.4	98.1	85.7
				85	low	197.6	124.1	726.6	932.0
			G. Mexico	26	medium	86.9	72.9	28.0	40.1
				85	high	264.4	131.9	-52.3	40.3
diplectrum formosum	0.47	0.94	SE U.S.	26	medium	37.9	34.8	154.4	125.4
				85	low	201.8	82.1	1902.5	967.6
			G. Mexico	26	low	29.1	23.5	34.3	28.8
				85	low	102.1	40.2	128.0	62.8
diplopteraster multipes	0.20	0.21	G. Alaska	26	low	90.1	63.7	-3.2	7.8
				85	medium	349.1	250.1	6.3	19.7

**Species**	**devPA**	**devBiom**	**Region**	**RCP**	**Uncert.**	**Shift**	**sd_shift**	**%Hab.**	**sd_hab**
dipsacaster borealis	0.23	0.77	E. Bering S.	26	low	132.2	107.9	-24.0	24.1
				85	low	479.4	122.6	-83.7	18.0
dipsacaster eximius	0.32	0.74	West U.S.	26	medium	156.4	171.1	28.1	42.7
				85	medium	755.1	377.2	408.6	236.5
dipturus laevis	0.25	0.89	E. Canada	26	high	279.2	428.6	152.8	502.5
				85	high	875.6	558.7	-86.8	21.4
doryteuthis opalescens	0.45	0.13	West U.S.	26	low	164.9	124.1	40.2	28.8
				85	low	749.9	189.3	255.1	139.1
doryteuthis pleii	0.46	0.87	G. Mexico	26	low	56.8	39.1	98.7	96.1
				85	medium	135.9	121.3	320.5	310.2
dromalia alexandri	0.51	0.84	West U.S.	26	high	48.1	110.0	266.1	298.3
				85	high	72.8	109.7	8067.6	20773.6
duva florida	0.34	0.80	E. Canada	26	low	110.1	52.4	-20.1	23.3
				85	low	258.3	155.8	-62.5	35.8
echeneis naucrates	0.26	0.98	SE U.S.	26	medium	12.6	10.8	15.2	23.1
				85	medium	115.3	89.9	18.7	14.3
			G. Mexico	26	medium	18.5	17.3	5.1	11.8
				85	medium	105.3	83.0	-55.4	23.6
echinarachnius parma	0.32	0.82	E. Canada	26	medium	185.3	177.2	-15.2	79.4
				85	medium	832.9	399.4	-77.8	23.2
	0.16	0.89	E. Bering S.	26	medium	69.5	56.4	-37.9	59.3
				85	low	139.1	68.6	-98.5	2.9
elassochirus cavimanus	0.21	0.91	E. Bering S.	26	low	60.0	41.7	-44.6	48.9
				85	low	150.2	63.3	-98.3	3.9
elassochirus tenuimanus	0.27	0.90	E. Bering S.	26	medium	180.9	131.7	0.3	78.6
				85	low	399.5	116.9	-65.2	57.6
eleginus gracilis	0.53	0.96	E. Bering S.	26	low	28.0	26.5	10.7	41.7
				85	high	353.5	1106.8	-17.0	108.7
embassichthys bathybius	0.70	0.96	G. Alaska	26	high	311.4	275.7	-7.5	15.2
				85	medium	1343.0	367.1	83.6	69.7
enchelyopus cimbrius	0.29	0.83	E. Canada	26	low	85.7	48.6	8.4	18.4
				85	low	304.2	89.2	11.5	26.4
engraulis eurystole	0.21	0.80	NE U.S.	26	medium	172.0	197.5	160.1	444.7
				85	high	544.1	605.2	3270.0	3332.8
			G. Mexico	26	medium	112.3	90.9	-69.4	26.7
				85	high	246.8	163.3	-98.1	3.2
engraulis mordax	0.33	0.89	West U.S.	26	low	236.5	123.8	35.0	21.0
				85	low	1221.3	410.1	138.4	90.7

Species	devPA	devBiom	Region	RCP	Uncert.	Shift	sd_shift	%Hab.	sd_hab
engyophrys senta	0.33	0.90	G. Mexico	26	low	44.4	42.6	3.8	25.1
				85	medium	135.6	109.3	-59.7	34.4
eopsetta jordani	0.61	0.95	G. Alaska	26	low	347.1	260.1	20.9	16.7
				85	low	1333.3	233.6	101.2	68.1
eptatretus deani	0.38	0.92	West U.S.	26	medium	180.3	136.1	12.8	23.7
				85	medium	751.7	322.4	103.5	63.0
eptatretus stoutii	0.26	0.86	G. Alaska	26	medium	300.2	230.9	19.7	42.1
				85	medium	1383.4	389.7	68.0	69.1
equetus lanceolatus	0.54	0.96	SE U.S.	26	high	89.1	116.3	321.5	389.9
				85	low	414.0	210.7	11854.3	14511.5
			G. Mexico	26	medium	30.1	33.0	36.7	46.3
				85	low	141.3	81.8	15.9	90.7
erimacrus isenbeckii	0.35	0.97	E. Bering S.	26	medium	104.1	70.3	-35.1	72.8
				85	low	192.3	116.7	-99.0	2.3
etropus crossotus	0.46	0.97	SE U.S.	26	low	11.9	8.7	16.6	17.6
				85	low	76.4	48.8	36.8	28.5
			G. Mexico	26	low	17.6	13.2	-5.8	6.8
				85	high	40.6	31.3	-54.1	14.7
etropus cyclosquamus	0.34	0.95	SE U.S.	26	medium	16.0	10.7	14.5	20.1
				85	low	82.5	62.1	1.6	28.4
			G. Mexico	26	medium	20.6	19.3	-8.3	9.1
				85	medium	53.3	44.4	-59.6	11.8
etropus microstomus	0.30	0.81	SE U.S.	26	high	92.0	123.7	19.2	44.1
				85	low	509.0	202.0	255.4	170.1
			G. Mexico	26	medium	13.2	9.6	-12.9	8.0
				85	medium	30.9	20.0	-22.8	16.0
etrumeus teres	0.27	0.80	SE U.S.	26	low	160.9	142.1	20.6	83.7
				85	low	655.0	273.5	298.5	268.3
			G. Mexico	26	low	38.1	31.3	-39.3	18.5
				85	low	76.4	32.4	-90.6	4.4
eualus fabricii	0.38	0.84	E. Canada	26	low	84.6	64.7	-26.3	28.5
				85	medium	189.1	95.7	-79.2	24.7
eualus gaimardii	0.51	0.86	E. Canada	26	low	80.2	68.6	-29.1	29.5
				85	medium	184.7	89.8	-81.6	22.5
eualus macilentus	0.57	0.89	E. Canada	26	low	115.7	85.1	-29.7	33.1
				85	low	280.9	147.4	-77.3	31.1
eualus macrophthalmus	0.34	0.91	G. Alaska	26	low	250.0	204.0	-1.2	12.5
				85	high	1027.3	414.8	8.0	31.0

Species	devPA	devBiom	Region	RCP	Uncert.	Shift	sd_shift	%Hab.	sd_hab
eucinostomus argenteus	0.27	0.86	SE U.S.	26	high	4.3	7.2	1398.8	2458.5
				85	medium	153.4	145.5	32183.4	52135.5
			G. Mexico	26	medium	42.2	33.6	146.4	146.2
				85	low	194.5	106.4	301.4	476.7
eucinostomus gula	0.39	0.86	SE U.S.	26	high	8.9	17.4	754.7	810.3
				85	low	40.9	27.2	13753.8	14891.1
			G. Mexico	26	low	28.3	18.0	288.6	305.3
				85	medium	59.2	36.9	3470.9	2738.8
eumesogrammus praecisus	0.46	0.90	E. Canada	26	low	159.6	127.6	-30.8	27.9
				85	low	444.8	196.4	-69.1	40.8
eumicrotremus orbis	0.25	0.91	E. Bering S.	26	low	67.1	40.9	-37.1	33.9
				85	low	193.6	77.6	-91.8	14.2
eumicrotremus spinosus	0.45	0.86	E. Canada	26	low	104.1	73.6	-27.0	31.3
				85	low	205.3	110.4	-79.1	25.5
eunoe depressa	0.21	0.94	E. Bering S.	26	low	68.3	46.9	-37.5	53.6
				85	low	143.9	69.6	-97.1	6.0
eunoe nodosa	0.28	0.94	E. Bering S.	26	low	58.6	44.9	-41.8	45.4
				85	low	159.5	66.8	-96.6	7.5
euspira pallida	0.29	0.91	E. Bering S.	26	low	24.4	12.7	-40.7	36.3
				85	medium	117.5	52.3	-95.7	6.1
evasterias echinosoma	0.36	0.96	E. Bering S.	26	medium	147.5	124.0	-3.6	139.7
				85	low	284.9	125.9	-98.9	2.5
fanellia compressa	0.33	0.88	E. Bering S.	26	medium	238.0	137.5	32.4	392.4
				85	medium	367.9	176.1	-99.9	0.3
farfantepenaeus aztecus	0.70	0.95	SE U.S.	26	low	13.4	13.3	8.2	18.5
				85	medium	175.6	142.5	-19.8	16.0
			G. Mexico	26	medium	20.2	20.1	-3.4	5.4
				85	low	33.3	25.3	0.7	11.0
farfantepenaeus duorarum	0.40	0.97	SE U.S.	26	medium	19.1	16.8	12.1	31.4
				85	low	164.0	104.6	-57.2	19.7
			G. Mexico	26	medium	26.5	18.9	-12.1	14.8
				85	medium	53.1	30.4	-68.4	9.3
fowlerichthys radiosus	0.40	0.97	SE U.S.	26	low	21.5	16.7	42.7	26.0
				85	high	98.2	157.5	245.8	100.6
			G. Mexico	26	medium	6.4	4.9	4.1	5.8
				85	high	14.8	8.2	36.7	13.2
fusitriton oregonensis	0.28	0.39	G. Alaska	26	low	123.9	79.0	3.9	7.7
				85	low	400.9	127.7	10.6	11.4

Species	devPA	devBiom	Region	RCP	Uncert.	Shift	sd_shift	%Hab.	sd_hab
gadus macrocephalus	0.49	0.14	E. Bering S.	26	low	44.9	28.6	-8.1	7.0
				85	low	116.5	54.7	-47.6	15.0
gadus morhua	0.45	0.89	E. Canada	26	low	111.6	67.8	-1.7	9.5
				85	medium	428.2	213.5	-34.0	18.1
gadus ogac	0.48	0.94	E. Canada	26	medium	129.0	128.7	-36.9	24.2
				85	medium	556.1	253.2	-65.7	53.0
genyonemus lineatus	0.54	0.92	West U.S.	26	medium	66.7	47.7	126.1	142.7
				85	medium	392.1	276.7	1341.8	1895.5
gersemia rubiformis	0.27	0.85	E. Canada	26	low	126.3	75.4	-13.3	21.6
				85	low	333.5	118.8	-57.5	40.9
	0.32	0.93	E. Bering S.	26	low	42.4	38.2	-13.3	24.4
				85	high	205.4	227.0	-70.2	18.9
gibbesia neglecta	0.44	0.94	SE U.S.	26	low	15.2	10.9	87.7	87.5
				85	low	42.6	15.9	589.7	448.8
			G. Mexico	26	medium	17.0	9.5	28.8	19.1
				85	high	52.2	27.1	150.9	60.7
glebocarcinus oregonensis	0.29	0.93	E. Bering S.	26	low	150.4	99.8	2.0	89.6
				85	low	333.5	90.9	-86.0	22.3
glyptocephalus cynoglossus	0.39	0.87	E. Canada	26	low	117.5	63.8	10.7	10.8
				85	low	398.6	183.0	9.0	22.4
glyptocephalus zachirus	0.37	0.15	G. Alaska	26	low	188.8	114.7	9.7	6.7
				85	low	654.5	93.9	15.3	18.8
gonatus onyx	0.30	0.95	G. Alaska	26	low	347.0	255.9	14.2	18.4
				85	low	1120.0	275.0	51.1	36.6
gorgonocephalus eucnemis	0.22	0.43	G. Alaska	26	low	384.6	239.8	12.0	17.9
				85	low	998.0	320.3	138.3	128.2
gymnachirus texae	0.35	0.90	G. Mexico	26	low	2.3	1.6	-18.4	32.6
				85	low	7.2	5.0	-93.5	7.8
gymnelus viridis	0.35	0.84	E. Canada	26	low	95.4	74.2	-25.8	27.2
				85	low	242.7	142.9	-75.3	28.0
gymnocanthus galeatus	0.22	0.96	E. Bering S.	26	low	107.2	80.6	-39.3	43.2
				85	low	305.0	84.6	-95.0	9.8
gymnocanthus pistilliger	0.49	0.96	E. Bering S.	26	high	88.5	62.9	-2.3	124.6
				85	high	214.5	95.1	-99.0	2.2
gymnothorax saxicola	0.37	0.88	SE U.S.	26	high	22.8	36.3	875.0	1409.1
				85	medium	252.4	171.0	21903.1	30057.6
			G. Mexico	26	low	34.7	31.7	102.2	95.5
				85	low	101.6	57.2	723.2	455.0

Species	devPA	devBiom	Region	RCP	Uncert.	Shift	sd_shift	%Hab.	sd_hab
gymnura altavela	0.45	0.96	SE U.S.	26	medium	27.4	28.3	-6.4	15.7
				85	low	503.1	255.2	-52.6	23.3
			G. Mexico	26	medium	72.0	56.9	-44.9	24.8
				85	high	168.1	133.9	-98.3	3.0
gymnura micrura	0.55	0.97	SE U.S.	26	low	20.7	14.6	3.3	7.5
				85	medium	284.9	194.2	28.5	44.9
			G. Mexico	26	low	26.6	22.7	-9.6	9.8
				85	medium	64.5	38.1	-64.3	12.6
gymothorax igromargiatus	0.24	0.89	SE U.S.	26	low	52.3	39.6	66.9	36.7
				85	low	257.7	108.5	282.4	139.6
			G. Mexico	26	medium	21.8	23.2	10.5	12.2
				85	low	65.4	39.8	-10.3	29.7
haemulon aurolineatum	0.42	0.93	SE U.S.	26	medium	68.7	73.9	254.7	270.3
				85	low	319.7	153.0	8641.7	7551.5
			G. Mexico	26	medium	17.6	15.6	59.3	51.4
				85	low	57.3	22.4	366.5	174.3
haemulon plumierii	0.47	0.96	SE U.S.	26	medium	81.8	95.7	380.2	500.4
				85	low	365.7	184.3	9806.2	9282.3
			G. Mexico	26	medium	19.3	20.1	66.1	67.7
				85	medium	39.6	32.9	208.2	207.4
halichondria panicea	0.15	0.83	E. Bering S.	26	medium	108.0	109.1	-29.9	30.4
				85	high	209.1	250.4	-93.1	9.6
halichondria sitiens	0.39	0.90	E. Bering S.	26	medium	244.6	140.8	33.1	371.0
				85	medium	453.8	213.6	-99.9	0.2
halieutichthys aculeatus	0.49	0.95	SE U.S.	26	medium	20.4	14.2	105.2	81.7
				85	medium	55.7	20.5	436.0	361.3
			G. Mexico	26	medium	33.9	33.0	-5.4	9.2
				85	low	112.3	63.8	-50.2	19.8
halocynthia aurantium	0.22	0.94	E. Bering S.	26	low	95.3	86.4	-44.3	40.9
				85	low	225.4	79.4	-92.6	17.8
harengula jaguana	0.39	0.97	SE U.S.	26	medium	57.8	63.6	144.6	142.1
				85	low	130.8	74.4	1571.9	1280.3
			G. Mexico	26	medium	24.9	24.2	36.1	31.5
				85	medium	64.1	43.6	164.5	116.0
helicolenus dactylopterus	0.43	0.68	NE U.S.	26	low	186.3	148.6	34.5	32.5
				85	low	714.1	233.8	111.8	93.8
			G. Mexico	26	medium	72.7	52.5	-48.4	15.9
				85	high	98.5	47.7	-84.9	8.7

Species	devPA	devBiom	Region	RCP	Uncert.	Shift	sd_shift	%Hab.	sd_hab
hemicaranx amblyrhynchus	0.34	0.96	SE U.S.	26	medium	11.7	9.3	29.0	21.9
				85	high	53.3	66.1	246.6	131.7
			G. Mexico	26	medium	30.2	16.3	29.6	23.7
				85	medium	73.7	55.2	264.5	137.5
hemilepidotus jordani	0.29	0.92	E. Bering S.	26	low	65.0	46.4	-35.0	53.4
				85	low	180.1	53.1	-97.0	5.3
hemilepidotus papilio	0.51	0.96	E. Bering S.	26	low	24.2	11.1	-46.8	48.3
				85	low	95.8	50.1	-98.5	3.2
hemitripterus americanus	0.32	0.89	E. Canada	26	low	107.2	70.4	-4.4	7.7
				85	low	445.0	165.3	-41.3	13.8
hemitripterus bolini	0.24	0.88	E. Bering S.	26	low	52.9	34.5	-27.5	40.2
				85	low	142.1	35.5	-93.1	11.5
henricia leviuscula	0.14	0.80	E. Bering S.	26	high	91.9	185.0	-29.0	43.4
				85	high	2676.4	2288.4	758.2	2263.6
hepatus epheliticus	0.32	0.97	SE U.S.	26	high	28.5	40.7	-4.6	8.1
				85	high	183.5	212.7	3.9	25.9
			G. Mexico	26	medium	23.9	28.0	-7.2	12.5
				85	medium	79.1	64.8	-7.5	30.9
heterozonias alternatus	0.61	0.95	G. Alaska	26	high	298.9	289.5	-4.8	13.4
				85	high	1388.8	273.8	32.2	37.3
hexagrammos decagrammus	0.18	0.12	E. Bering S.	26	low	27.4	16.0	1.9	6.1
				85	low	100.6	43.0	6.8	9.7
hexagrammos stelleri	0.40	0.91	E. Bering S.	26	high	72.5	56.0	2.2	49.5
				85	medium	135.2	77.2	-73.2	39.8
hippasteria californica	0.40	0.93	G. Alaska	26	low	385.8	324.3	16.4	22.6
				85	low	1394.1	291.5	104.7	49.9
hippocampus erectus	0.23	0.96	SE U.S.	26	low	7.5	5.8	55.3	42.8
				85	medium	18.6	13.9	609.9	260.8
			G. Mexico	26	low	8.6	6.7	38.2	32.0
				85	low	17.7	9.4	432.0	186.8
hippoglossina oblonga	0.42	0.81	NE U.S.	26	low	50.2	39.4	27.3	27.7
				85	medium	307.6	271.9	69.5	50.2
hippoglossoides elassodon	0.34	0.71	G. Alaska	26	medium	166.6	154.8	16.0	33.4
				85	low	708.2	352.4	12.8	46.0
hippoglossoides platessoides	0.60	0.84	E. Canada	26	low	88.3	57.4	-4.3	13.8
				85	medium	334.4	182.8	-23.0	29.9
hippoglossus hippoglossus	0.29	0.94	E. Canada	26	low	122.1	69.3	5.5	11.6
				85	medium	440.9	262.1	-18.6	27.3

Species	devPA	devBiom	Region	RCP	Uncert.	Shift	sd_shift	%Hab.	sd_hab
hippoglossus stenolepis	0.33	0.28	E. Bering S.	26	low	81.0	50.8	7.3	5.4
				85	low	277.1	57.9	8.8	12.2
histioteuthis heteropsis	0.30	0.87	West U.S.	26	medium	217.0	205.5	8.9	14.6
				85	low	1161.6	388.3	50.6	38.7
homarus americanus	0.30	0.83	NE U.S.	26	low	60.4	45.0	7.9	23.3
				85	medium	310.0	222.5	3.4	42.4
homaxinella amphispicula	0.37	0.96	E. Bering S.	26	high	105.1	132.1	-44.4	48.3
				85	medium	168.5	106.3	-97.9	4.4
hoplunnis macrura	0.34	0.89	G. Mexico	26	low	0.5	0.4	1.0	36.3
				85	low	1.5	1.7	-75.3	24.4
hyas araneus	0.37	0.82	E. Canada	26	high	305.3	302.4	272.8	800.3
				85	high	528.9	405.2	-60.0	47.3
hyas coarctatus	0.39	0.82	E. Canada	26	medium	282.7	269.0	235.1	706.4
				85	high	596.3	431.6	-48.6	68.1
	0.54	0.95	E. Bering S.	26	medium	77.9	52.2	-36.6	65.5
				85	low	163.1	80.7	-98.6	3.0
hyas lyratus	0.24	0.21	E. Bering S.	26	low	57.4	35.8	6.7	7.9
				85	medium	145.3	89.1	32.6	18.0
hydrolagus colliei	0.53	0.96	G. Alaska	26	low	290.7	191.0	24.3	19.9
				85	low	1239.3	286.1	128.2	90.1
icelinus filamentosus	0.34	0.97	G. Alaska	26	low	284.2	195.4	20.8	27.8
				85	low	1304.2	360.9	88.0	59.7
icelus spatula	0.56	0.91	E. Canada	26	low	116.4	78.4	-34.2	36.7
				85	medium	245.1	112.4	-79.7	34.8
	0.26	0.96	E. Bering S.	26	medium	70.1	56.9	-37.3	68.7
				85	medium	116.2	73.3	-98.8	2.5
icelus spiniger	0.46	0.94	E. Bering S.	26	medium	59.2	51.5	-40.7	46.9
				85	low	131.5	33.7	-95.5	11.1
illex illecebrosus	0.37	0.72	NE U.S.	26	low	73.3	42.0	42.8	39.6
				85	medium	286.7	192.7	224.8	87.0
isopsetta isolepis	0.30	0.18	G. Alaska	26	low	261.9	202.1	48.7	52.6
				85	low	796.4	250.2	356.7	208.2
kathetostoma albigutta	0.44	0.91	SE U.S.	26	medium	70.9	76.6	192.6	145.8
				85	low	296.3	126.9	1308.7	1241.7
			G. Mexico	26	medium	52.7	38.8	27.2	36.4
				85	medium	80.1	79.3	15.5	118.8
labidochirus splendescens	0.35	0.94	E. Bering S.	26	medium	60.2	35.3	-35.4	53.5
				85	low	147.9	47.7	-96.1	7.3

Species	devPA	devBiom	Region	RCP	Uncert.	Shift	sd_shift	%Hab.	sd_hab
lagocephalus laevigatus	0.38	0.96	SE U.S.	26	low	11.8	8.4	19.6	18.9
				85	low	51.0	25.2	76.6	34.9
			G. Mexico	26	low	12.9	8.9	10.2	9.3
				85	medium	33.8	16.9	60.3	29.0
lagodon rhomboides	0.44	0.95	SE U.S.	26	medium	17.3	21.3	-0.9	16.8
				85	medium	219.6	181.1	-37.4	13.1
			G. Mexico	26	medium	22.7	28.3	3.3	9.8
				85	medium	54.9	53.3	55.1	34.0
laqueus californianus	0.24	0.74	E. Bering S.	26	low	54.3	43.2	-14.8	59.4
				85	low	177.7	69.1	-83.1	34.9
larimus fasciatus	0.45	0.95	SE U.S.	26	low	22.1	15.2	-10.6	12.3
				85	medium	265.5	226.6	-1.5	43.1
			G. Mexico	26	low	36.9	21.3	8.0	11.0
				85	low	76.6	27.1	65.8	43.3
lebbeus groenlandicus	0.39	0.85	E. Canada	26	medium	125.3	93.0	-24.4	20.1
				85	medium	231.2	155.6	-76.2	25.9
lebbeus polaris	0.47	0.85	E. Canada	26	low	153.9	95.9	-13.0	21.0
				85	low	318.5	133.9	-66.8	30.8
leiolambrus nitidus	0.31	0.92	G. Mexico	26	medium	47.4	42.8	100.8	127.8
				85	low	176.0	114.1	299.8	353.9
leiostomus xanthurus	0.48	0.94	SE U.S.	26	low	22.0	18.1	-6.8	8.1
				85	medium	278.9	250.4	26.5	54.5
			G. Mexico	26	low	16.9	8.7	7.3	7.8
				85	low	31.1	16.0	34.8	21.6
lepophidium brevibarbe	0.43	0.90	G. Mexico	26	low	0.6	0.5	11.6	53.7
				85	low	1.7	2.2	-80.3	23.8
lepophidium jeannae	0.37	0.89	SE U.S.	26	high	52.3	85.8	276.5	347.1
				85	low	368.0	174.7	2674.3	2790.6
			G. Mexico	26	medium	49.3	56.3	16.2	34.4
				85	medium	157.0	128.0	-40.2	59.5
lepophidium profundorum	0.36	0.69	NE U.S.	26	low	76.3	63.4	17.9	30.7
				85	medium	500.1	291.4	46.6	69.8
leptagonus decagonus	0.58	0.93	E. Canada	26	low	153.7	99.9	-18.6	18.2
				85	low	424.9	151.5	-60.7	29.0
leptasterias arctica	0.39	0.95	E. Bering S.	26	medium	85.3	69.9	-21.5	105.5
				85	low	148.7	84.3	-99.4	1.4

Species	devPA	devBiom	Region	RCP	Uncert.	Shift	sd_shift	%Hab.	sd_hab
leptasterias polaris	0.33	0.85	E. Canada	26	medium	186.6	170.2	-17.1	32.9
				85	medium	639.6	475.7	-74.0	33.7
	0.55	0.97	E. Bering S.	26	low	36.3	20.8	-43.3	46.0
				85	low	85.0	39.3	-97.0	5.6
leptoclinus maculatus	0.51	0.88	E. Canada	26	low	123.9	82.8	-16.8	21.6
				85	low	280.0	116.1	-70.7	27.6
	0.24	0.93	E. Bering S.	26	low	46.9	27.4	-34.3	37.4
				85	low	128.4	33.7	-92.6	11.8
lethasterias nanimensis	0.19	0.90	E. Bering S.	26	low	59.9	39.5	-39.7	38.9
				85	low	184.3	57.5	-94.3	11.5
leucoraja erinacea	0.54	0.88	E. Canada	26	high	272.6	194.6	-32.5	32.9
				85	high	765.2	556.9	-77.3	17.6
leucoraja garmani	0.41	0.80	NE U.S.	26	medium	168.5	164.9	23.0	58.9
				85	low	647.6	235.5	704.6	376.0
			G. Mexico	26	high	73.1	75.9	-60.5	25.6
				85	medium	115.3	122.2	-99.3	0.6
leucoraja ocellata	0.40	0.84	NE U.S.	26	low	118.7	104.9	-16.6	16.0
				85	medium	455.8	270.1	-64.4	28.4
leuroglossus schmidti	0.35	0.84	West U.S.	26	low	208.5	153.6	21.1	18.6
				85	low	805.7	223.2	75.0	30.9
libinia emarginata	0.32	0.90	SE U.S.	26	high	12.8	26.1	434.6	505.3
				85	medium	50.3	37.9	11514.1	7792.4
			G. Mexico	26	high	38.2	40.8	197.5	222.4
				85	high	54.7	56.5	4951.6	5014.9
limanda aspera	0.76	0.96	E. Bering S.	26	medium	69.3	58.3	103.3	375.8
				85	medium	133.7	66.2	-97.7	3.3
limanda ferruginea	0.40	0.84	E. Canada	26	low	209.0	131.7	-0.3	17.1
				85	medium	710.1	284.2	-42.4	36.5
limanda proboscidea	0.66	0.96	E. Bering S.	26	medium	54.2	64.7	-15.9	49.2
				85	high	208.0	209.4	-94.3	6.6
limanda sakhalinensis	0.53	0.97	E. Bering S.	26	low	19.9	18.4	-34.1	31.8
				85	medium	71.2	68.7	-90.8	14.4
limulus polyphemus	0.42	0.90	SE U.S.	26	high	21.9	12.0	4.4	13.9
				85	medium	85.7	69.7	134.7	92.5
			G. Mexico	26	low	28.5	20.9	53.0	43.6
				85	high	43.1	18.8	506.5	266.7
liparis atlanticus	0.13	0.69	NE U.S.	26	high	150.8	151.8	34.5	37.7
				85	medium	347.4	211.9	171.5	92.4

Species	devPA	devBiom	Region	RCP	Uncert.	Shift	sd_shift	%Hab.	sd_hab
liparis gibbus	0.41	0.95	E. Bering S.	26	high	30.6	17.5	-43.5	64.8
				85	high	203.6	353.0	-99.6	1.2
liponema brevicorne	0.32	0.27	G. Alaska	26	low	172.2	126.7	6.7	13.3
				85	low	706.5	261.5	47.6	38.7
lithodes aequispinus	0.46	0.92	E. Bering S.	26	medium	138.3	99.7	-45.9	40.9
				85	low	262.7	112.7	-96.7	7.8
lithodes couesi	0.44	0.81	West U.S.	26	medium	173.9	189.6	21.2	45.4
				85	medium	673.4	423.7	554.6	338.2
lithodes maja	0.29	0.88	E. Canada	26	high	108.6	100.5	13.5	34.8
				85	high	278.8	240.7	-40.4	51.6
litopenaeus setiferus	0.47	0.95	SE U.S.	26	low	16.1	12.6	88.6	103.5
				85	medium	43.0	30.2	867.1	925.7
			G. Mexico	26	medium	17.9	10.4	33.6	32.9
				85	low	42.3	25.8	217.9	162.4
loligo pealeii	0.43	0.63	NE U.S.	26	medium	154.9	166.7	49.4	62.0
				85	high	345.4	414.9	446.1	207.2
			G. Mexico	26	medium	104.2	106.8	-53.2	31.2
				85	medium	285.8	229.9	-87.7	12.5
lolliguncula brevis	0.56	0.96	SE U.S.	26	low	13.2	10.1	15.4	20.1
				85	medium	182.9	159.7	30.7	46.3
			G. Mexico	26	medium	13.4	11.9	-12.3	10.5
				85	low	53.5	41.2	-62.9	14.7
lophaster furcilliger	0.22	0.84	G. Alaska	26	medium	294.8	318.2	78.0	102.2
				85	medium	1034.9	673.6	553.9	220.7
lophius americanus	0.30	0.53	E. Canada	26	high	320.3	224.2	-4.2	29.4
				85	high	614.6	381.4	-21.2	46.9
			G. Mexico	26	low	54.3	25.5	-41.8	20.0
				85	medium	79.1	63.3	-92.0	3.4
lopholithodes foraminatus	0.20	0.89	West U.S.	26	low	241.3	208.6	19.9	16.9
				85	low	1357.5	404.8	55.6	43.0
luidia clathrata	0.33	0.89	G. Mexico	26	medium	61.9	45.4	132.4	130.3
				85	low	93.3	47.8	1036.6	618.4
luidia foliolata	0.48	0.92	G. Alaska	26	low	265.0	189.4	22.5	16.7
				85	low	1074.7	256.3	87.2	57.0
lumpenus lampretaeformis	0.33	0.90	E. Canada	26	low	87.4	42.5	-9.2	8.3
				85	low	253.0	88.7	-43.0	27.8
lumpenus sagitta	0.20	0.85	E. Bering S.	26	medium	40.3	35.1	-9.0	28.8
				85	medium	110.3	108.4	-69.4	32.6

Species	devPA	devBiom	Region	RCP	Uncert.	Shift	sd_shift	%Hab.	sd_hab
lutjanus campechanus	0.56	0.97	SE U.S.	26	medium	74.5	75.3	95.0	85.2
				85	medium	190.0	129.6	856.8	560.8
			G. Mexico	26	medium	16.2	21.1	7.9	8.5
				85	medium	28.1	28.1	32.3	14.7
lutjanus griseus	0.44	0.97	SE U.S.	26	high	83.4	164.0	889.2	1356.2
				85	low	463.0	234.3	96662.7	206810.4
			G. Mexico	26	medium	37.6	36.7	70.3	79.5
				85	low	205.6	114.0	71.2	177.2
lutjanus synagris	0.47	0.98	SE U.S.	26	medium	71.1	76.0	187.3	159.0
				85	low	418.2	135.8	5011.0	4890.9
			G. Mexico	26	low	35.0	29.1	103.3	85.5
				85	low	105.9	39.5	998.9	478.2
lycenchelys crotalinus	0.60	0.82	G. Alaska	26	medium	162.2	145.9	-15.4	21.2
				85	high	775.3	452.5	97.1	175.5
lycodes brevipes	0.40	0.36	E. Bering S.	26	low	131.8	81.6	-7.8	11.0
				85	high	368.7	216.5	-21.3	36.8
lycodes cortezianus	0.44	0.95	G. Alaska	26	low	255.6	196.1	2.1	10.9
				85	low	1128.6	274.1	2.8	37.7
lycodes diapterus	0.37	0.91	G. Alaska	26	low	242.4	167.0	0.2	11.3
				85	medium	1018.7	290.8	-23.8	39.4
lycodes esmarkii	0.38	0.86	E. Canada	26	low	113.8	73.6	-3.8	7.9
				85	low	413.3	149.2	-43.0	29.2
lycodes pacificus	0.39	0.95	West U.S.	26	low	239.0	160.7	49.5	42.1
				85	low	1000.8	257.6	290.3	113.6
lycodes palearis	0.40	0.16	E. Bering S.	26	low	28.5	23.7	-4.7	6.7
				85	medium	74.8	59.2	-7.2	19.7
lycodes raridens	0.57	0.96	E. Bering S.	26	medium	25.4	12.5	-42.3	40.6
				85	low	111.9	79.1	-97.4	4.2
lycodes reticulatus	0.55	0.86	E. Canada	26	low	134.1	82.1	-20.0	26.6
				85	low	284.2	126.2	-72.4	30.7
lycodes vahlii	0.44	0.84	E. Canada	26	medium	136.8	108.5	-0.1	21.5
				85	low	388.0	132.8	-37.5	45.0
lyopsetta exilis	0.53	0.88	G. Alaska	26	low	296.6	205.4	24.4	21.4
				85	high	958.3	329.8	126.8	57.3
macrourus berglax	0.78	0.89	E. Canada	26	medium	160.0	131.7	3.4	13.2
				85	low	444.3	145.7	-15.4	41.8
mactromeris polynyma	0.41	0.93	E. Bering S.	26	medium	46.8	35.4	-11.4	34.7
				85	low	195.3	82.7	-80.2	15.0

Species	devPA	devBiom	Region	RCP	Uncert.	Shift	sd_shift	%Hab.	sd_hab
malacocottus zonurus	0.45	0.78	E. Bering S.	26	medium	145.9	66.6	-51.2	42.7
				85	medium	303.5	179.8	-98.3	3.2
malacoraja senta	0.32	0.85	E. Canada	26	low	121.6	70.4	10.6	12.7
				85	medium	464.6	249.0	2.9	27.0
malacosteus niger	0.57	0.91	E. Canada	26	medium	182.9	115.4	-1.3	19.5
				85	low	352.9	161.1	5.2	40.8
mallotus villosus	0.64	0.86	E. Canada	26	low	154.2	107.8	-12.5	23.0
				85	low	346.0	181.6	-63.2	36.5
	0.45	0.92	E. Bering S.	26	high	41.2	26.8	-25.8	32.2
				85	medium	102.6	56.8	-89.9	14.2
maurolicus weitzmani	0.28	0.62	NE U.S.	26	high	214.5	219.7	90.8	114.1
				85	low	422.5	211.3	326.7	262.3
mediaster aequalis	0.23	0.19	G. Alaska	26	low	171.2	127.1	15.5	12.9
				85	low	562.6	136.3	35.9	26.1
melanogrammus aeglefinus	0.49	0.89	E. Canada	26	low	131.9	91.2	3.4	10.1
				85	low	495.2	253.2	-26.7	36.7
melanostigma atlanticum	0.30	0.80	NE U.S.	26	medium	136.0	134.6	2.7	30.1
				85	low	882.8	258.7	-26.6	46.8
menidia menidia	0.48	0.70	E. Canada	26	high	379.0	417.7	-32.4	44.4
				85	high	802.0	647.3	-81.9	21.1
menippe mercenaria	0.37	0.93	SE U.S.	26	high	51.8	33.8	104.9	82.1
				85	medium	122.4	75.6	881.5	919.7
			G. Mexico	26	medium	34.5	16.3	66.1	53.6
				85	medium	93.9	48.0	366.2	254.3
menticirrhus americanus	0.55	0.92	SE U.S.	26	low	7.7	5.0	18.1	17.1
				85	medium	67.4	72.0	56.8	43.9
			G. Mexico	26	low	16.2	8.2	-5.8	7.9
				85	medium	34.2	21.5	-31.8	14.4
menticirrhus littoralis	0.47	0.97	SE U.S.	26	low	7.0	5.9	37.3	26.0
				85	medium	57.0	69.5	197.9	78.1
			G. Mexico	26	high	9.1	5.6	17.0	11.2
				85	medium	19.1	11.4	73.8	25.6
menticirrhus saxatilis	0.33	0.70	SE U.S.	26	medium	33.4	34.9	-10.6	14.8
				85	low	431.2	232.6	-56.0	12.7
			G. Mexico	26	high	56.7	54.5	-35.3	22.5
				85	medium	82.4	96.7	-94.1	3.6
merluccius albidus	0.46	0.81	NE U.S.	26	low	104.7	92.7	55.9	54.3
				85	medium	454.5	276.0	359.6	252.8

Species	devPA	devBiom	Region	RCP	Uncert.	Shift	sd_shift	%Hab.	sd_hab
merluccius bilinearis	0.51	0.73	E. Canada	26	low	133.5	106.5	10.4	20.7
				85	low	592.6	246.2	-2.6	36.1
merluccius productus	0.62	0.93	G. Alaska	26	low	281.8	208.8	21.9	18.3
				85	low	1175.3	264.2	100.7	53.6
metacarcinus magister	0.57	0.95	G. Alaska	26	medium	168.4	203.3	231.2	434.1
				85	high	785.1	738.1	10650.2	34516.8
metridium farcimen	0.30	0.13	G. Alaska	26	low	90.1	82.9	18.1	11.2
				85	low	374.6	163.2	80.4	29.9
microgadus proximus	0.35	0.88	West U.S.	26	low	332.3	250.0	24.5	21.4
				85	low	1671.2	370.9	158.4	91.8
micropogonias undulatus	0.55	0.93	SE U.S.	26	medium	29.9	23.0	-8.3	13.2
				85	high	286.1	278.4	300.1	303.2
			G. Mexico	26	low	30.9	20.2	38.3	42.2
				85	low	102.8	43.2	513.7	374.0
microstomus pacificus	0.58	0.34	G. Alaska	26	low	258.1	170.3	9.1	8.8
				85	low	880.9	134.4	7.1	21.7
modiolus modiolus	0.19	0.91	E. Bering S.	26	low	63.3	42.1	-31.1	42.4
				85	low	194.4	55.6	-91.7	14.9
molpadia intermedia	0.23	0.31	G. Alaska	26	low	88.4	56.7	6.0	11.0
				85	medium	401.7	238.5	22.5	21.1
monacanthus ciliatus	0.56	0.90	SE U.S.	26	high	39.1	75.1	1392.7	2385.8
				85	medium	349.6	267.5	70402.9	96375.3
			G. Mexico	26	low	25.6	20.6	119.6	127.4
				85	low	117.1	65.5	551.6	580.4
moreiradromia antillensis	0.48	0.91	G. Mexico	26	medium	28.3	27.1	87.9	101.3
				85	medium	123.8	91.2	115.0	245.4
morone saxatilis	0.36	0.89	NE U.S.	26	medium	73.0	91.9	309.8	707.1
				85	high	362.5	503.3	3176.3	7799.3
mullus auratus	0.38	0.87	SE U.S.	26	high	41.7	64.3	560.1	1059.1
				85	medium	294.9	156.1	6925.0	6478.8
			G. Mexico	26	medium	47.1	54.3	68.6	72.9
				85	high	72.6	102.6	469.9	445.3
musculus discors	0.25	0.95	E. Bering S.	26	medium	76.7	47.8	-20.4	108.5
				85	medium	170.7	85.4	-98.8	2.7
mustelus canis	0.42	0.88	SE U.S.	26	medium	126.4	94.8	-16.0	27.5
				85	low	474.9	156.5	74.3	106.3
			G. Mexico	26	low	50.3	32.8	-8.9	9.6
				85	low	89.4	60.2	53.1	41.7

Species	devPA	devBiom	Region	RCP	Uncert.	Shift	sd_shift	%Hab.	sd_hab
mycale loveni	0.32	0.77	E. Bering S.	26	low	157.5	101.3	-40.5	48.5
				85	low	462.5	210.0	-95.9	9.5
myliobatis freminvillii	0.49	0.96	SE U.S.	26	low	34.7	32.7	-6.9	11.8
				85	low	607.0	296.9	-6.6	48.3
			G. Mexico	26	low	71.7	54.4	-34.8	23.5
				85	high	198.9	113.9	-95.5	7.8
myoxocephalus octodecemspinosus	0.41	0.84	E. Canada	26	low	144.7	89.1	-4.6	11.8
				85	low	525.6	141.9	-45.0	29.0
myoxocephalus scorpius	0.42	0.96	E. Canada	26	high	63.7	49.9	-22.1	24.1
				85	medium	257.5	174.1	-59.7	36.2
myxine glutinosa	0.27	0.81	E. Canada	26	low	88.2	68.3	21.0	23.1
				85	low	541.6	222.3	25.3	47.2
myxoderma platyacanthum	0.43	0.94	G. Alaska	26	medium	177.3	111.1	25.3	29.8
				85	high	404.3	272.1	143.5	102.7
nearchaster aciculosus	0.52	0.81	West U.S.	26	high	331.5	199.6	17.6	53.7
				85	low	1033.8	387.1	718.8	545.1
nemichthys scolopaceus	0.40	0.86	E. Canada	26	medium	144.4	101.1	8.9	16.9
				85	low	474.0	161.1	3.4	32.8
neomerinthe hemingwayi	0.36	0.88	SE U.S.	26	medium	24.7	21.0	177.7	140.7
				85	low	77.2	36.1	4275.5	2373.9
			G. Mexico	26	low	17.9	12.0	115.7	98.9
				85	low	38.0	19.6	3927.5	2983.3
neptunea amianta	0.37	0.19	E. Bering S.	26	low	150.4	98.6	-11.1	8.0
				85	low	387.8	120.9	-48.0	15.4
neptunea borealis	0.37	0.92	E. Bering S.	26	low	46.7	31.0	-31.4	44.1
				85	low	157.0	71.0	-93.6	10.0
neptunea heros	0.57	0.94	E. Bering S.	26	low	67.8	57.7	-29.9	43.7
				85	low	221.0	97.5	-92.5	12.9
neptunea lyrata	0.43	0.90	E. Bering S.	26	medium	95.0	83.7	-25.5	58.8
				85	medium	217.4	50.3	-88.9	23.7
neptunea pribiloffensis	0.56	0.92	E. Bering S.	26	low	51.1	31.5	-31.9	51.5
				85	low	143.2	48.7	-92.4	15.9
neptunea ventricosa	0.53	0.97	E. Bering S.	26	medium	87.1	61.9	-18.6	117.7
				85	medium	165.4	98.4	-99.0	2.2
nezumia bairdii	0.63	0.85	E. Canada	26	medium	130.8	104.9	12.9	14.7
				85	low	364.6	152.9	6.8	46.4
nezumia liolepis	0.50	0.85	West U.S.	26	high	183.0	242.0	31.3	49.2
				85	medium	1130.8	591.5	749.2	551.8

Species	devPA	devBiom	Region	RCP	Uncert.	Shift	sd_shift	%Hab.	sd_hab
nezumia stelgidolepis	0.38	0.89	West U.S.	26	high	103.9	134.5	34.5	29.3
				85	medium	283.3	225.4	260.4	167.0
occella dodecaedron	0.44	0.92	E. Bering S.	26	low	37.4	35.8	-25.9	37.6
				85	medium	181.8	126.2	-91.5	10.2
octopoteuthis deletron	0.50	0.96	G. Alaska	26	medium	296.0	289.2	0.5	18.8
				85	medium	1490.8	384.8	72.3	51.6
octopus californicus	0.47	0.98	West U.S.	26	medium	310.7	277.8	48.7	52.2
				85	low	1241.1	356.3	239.9	141.5
ogcocephalus declivirostris	0.36	0.91	G. Mexico	26	high	53.7	55.5	21.4	40.3
				85	low	210.1	136.7	-20.2	66.5
ogcocephalus parvus	0.31	0.96	SE U.S.	26	low	89.9	78.1	209.6	214.6
				85	low	281.9	163.5	2156.3	1942.5
			G. Mexico	26	medium	66.9	72.2	-4.4	21.1
				85	medium	283.3	141.9	-77.0	25.8
ogcocephalus radiatus	0.27	0.88	G. Mexico	26	medium	6.0	3.5	23.6	22.0
				85	medium	15.6	10.0	273.1	97.4
oncorhynchus keta	0.18	0.75	E. Bering S.	26	low	86.1	58.8	-5.2	23.6
				85	medium	313.5	164.9	-43.3	74.7
oncorhynchus tshawytscha	0.23	0.05	E. Bering S.	26	low	54.3	45.5	6.9	6.1
				85	low	229.9	49.2	10.7	18.0
ophidion grayi	0.22	0.88	SE U.S.	26	high	86.4	86.1	312.1	368.9
				85	low	289.0	144.0	10748.3	13151.8
			G. Mexico	26	medium	44.3	46.9	20.0	35.8
				85	low	268.2	144.6	-59.7	43.9
ophidion holbrookii	0.37	0.93	SE U.S.	26	high	82.3	91.2	269.0	297.4
				85	low	309.4	165.8	8567.6	8573.6
			G. Mexico	26	medium	24.5	24.6	34.2	44.4
				85	low	89.2	52.6	3.8	88.5
ophidion marginatum	0.30	0.78	SE U.S.	26	medium	27.3	28.8	7.9	8.9
				85	low	399.7	166.2	40.2	37.9
			G. Mexico	26	low	23.4	20.4	-23.7	13.4
				85	medium	55.3	39.1	-76.4	8.1
ophidion welshi	0.31	0.88	SE U.S.	26	high	48.9	82.5	233.7	248.7
				85	low	294.6	151.4	4441.6	7667.3
			G. Mexico	26	medium	36.6	45.6	38.4	32.0
				85	low	182.5	116.6	16.8	56.9
ophiodon elongatus	0.40	0.91	G. Alaska	26	low	385.9	295.8	27.4	37.9
				85	low	1574.8	333.7	134.7	83.9

Species	devPA	devBiom	Region	RCP	Uncert.	Shift	sd_shift	%Hab.	sd_hab
ophiolepis elegans	0.33	0.87	G. Mexico	26	medium	54.6	54.5	50.4	59.0
				85	medium	219.0	146.9	7.6	109.4
ophiopholis aculeata	0.23	0.76	E. Bering S.	26	low	66.7	46.2	-46.4	35.2
				85	low	143.8	48.9	-96.4	8.3
opisthonema oglinum	0.48	0.96	SE U.S.	26	low	13.5	9.1	7.3	11.1
				85	medium	136.0	135.1	59.0	48.3
			G. Mexico	26	low	11.8	6.7	5.2	7.1
				85	medium	32.8	18.6	4.1	18.8
opisthoteuthis californiana	0.32	0.92	West U.S.	26	medium	345.2	373.5	12.3	13.7
				85	low	1771.7	410.9	50.6	36.6
oregonia gracilis	0.20	0.18	E. Bering S.	26	low	41.2	35.6	12.7	23.2
				85	low	167.7	70.5	45.2	39.4
orthasterias koehleri	0.16	0.19	E. Bering S.	26	low	63.2	42.3	3.1	12.2
				85	low	223.2	55.6	-22.9	20.0
orthopristis chrysoptera	0.42	0.95	SE U.S.	26	low	27.6	26.6	-13.5	15.4
				85	medium	394.7	258.1	-54.9	16.3
			G. Mexico	26	medium	17.9	22.6	-14.0	11.8
				85	high	43.5	41.0	-34.1	13.5
ovalipes floridaus	0.31	0.85	G. Mexico	26	medium	42.1	31.8	176.3	187.6
				85	high	73.1	64.1	9245.1	9954.5
ovalipes ocellatus	0.36	0.82	SE U.S.	26	low	182.6	146.7	37.1	61.3
				85	low	638.2	161.2	218.4	235.2
ovalipes stephensoni	0.53	0.89	SE U.S.	26	low	13.2	11.6	20.6	27.2
				85	medium	105.9	101.4	-20.8	46.6
			G. Mexico	26	medium	53.6	42.0	-27.7	20.9
				85	medium	147.9	55.0	-91.6	11.1
pagrus pagrus	0.40	0.87	SE U.S.	26	medium	9.1	11.9	514.3	623.8
				85	medium	47.1	45.1	23662.4	18074.5
			G. Mexico	26	high	16.8	10.8	183.4	180.9
				85	medium	31.7	24.5	10745.9	12338.2
pagurus aleuticus	0.32	0.86	E. Bering S.	26	low	74.6	62.0	-19.5	51.6
				85	low	189.1	65.7	-80.9	39.2
pagurus capillatus	0.23	0.86	E. Bering S.	26	low	76.6	51.6	-32.1	53.3
				85	low	190.0	64.2	-94.8	10.2
pagurus confragosus	0.25	0.84	E. Bering S.	26	low	62.3	50.6	-21.8	59.4
				85	low	157.1	43.0	-88.3	27.8
pagurus kennerlyi	0.28	0.93	E. Bering S.	26	low	94.2	68.4	-24.1	41.4
				85	low	265.6	62.7	-81.3	32.1

Species	devPA	devBiom	Region	RCP	Uncert.	Shift	sd_shift	%Hab.	sd_hab
pagurus ochotensis	0.45	0.95	E. Bering S.	26	medium	37.6	30.9	-5.9	107.7
				85	medium	93.0	32.3	-96.8	4.7
pagurus pollicaris	0.48	0.94	SE U.S.	26	low	21.6	19.6	98.1	113.3
				85	low	57.9	20.6	652.0	597.1
			G. Mexico	26	medium	21.0	19.5	18.0	14.2
				85	high	48.7	24.2	36.9	37.2
pagurus rathbuni	0.46	0.95	E. Bering S.	26	low	49.4	32.0	-35.1	55.0
				85	low	122.6	45.3	-95.5	8.6
pagurus trigonocheirus	0.32	0.89	E. Bering S.	26	low	84.0	61.8	-35.7	52.6
				85	low	178.9	86.6	-95.3	10.4
pandalopsis ampla	0.51	0.95	West U.S.	26	high	355.3	264.3	28.6	64.6
				85	medium	1920.1	722.0	546.1	480.9
pandalopsis dispar	0.38	0.20	G. Alaska	26	low	157.9	121.3	28.7	21.8
				85	low	576.9	270.6	136.9	63.7
pandalus borealis	0.51	0.85	E. Canada	26	low	145.9	88.0	-2.1	16.1
				85	low	351.4	126.4	-49.6	38.2
pandalus eous	0.28	0.81	E. Bering S.	26	medium	39.8	49.9	-42.5	34.4
				85	low	151.1	46.4	-91.9	15.4
pandalus goniurus	0.33	0.89	E. Bering S.	26	high	116.1	94.3	75.5	218.4
				85	high	328.4	284.3	-48.7	82.6
pandalus jordani	0.26	0.17	G. Alaska	26	low	248.9	168.6	18.7	13.2
				85	low	918.9	162.2	42.9	35.5
pandalus montagui	0.48	0.85	E. Canada	26	low	137.9	83.4	-11.2	23.7
				85	low	263.7	123.7	-69.4	29.5
pandalus platyceros	0.25	0.92	West U.S.	26	low	170.7	137.6	27.1	19.3
				85	low	600.2	237.4	138.8	64.8
pandalus tridens	0.24	0.09	E. Bering S.	26	low	57.5	28.0	-14.3	15.5
				85	low	134.4	40.7	-60.6	13.7
pannychia moseleyi	0.34	0.91	West U.S.	26	low	214.4	171.9	24.5	32.3
				85	medium	802.6	296.9	103.5	69.2
paractinostola faeculenta	0.59	0.92	G. Alaska	26	low	241.8	201.2	11.8	22.1
				85	medium	1169.1	335.5	71.9	58.0
paralichthys albigutta	0.33	0.96	SE U.S.	26	medium	59.2	77.0	-18.2	14.7
				85	high	451.4	408.7	-21.3	32.2
			G. Mexico	26	medium	20.7	23.9	-10.1	13.4
				85	high	76.0	38.0	-18.3	27.5
paralichthys dentatus	0.46	0.85	NE U.S.	26	high	52.9	74.6	63.6	77.9
				85	high	137.8	153.3	277.2	243.7

Species	devPA	devBiom	Region	RCP	Uncert.	Shift	sd_shift	%Hab.	sd_hab
paralichthys lethostigma	0.30	0.97	SE U.S.	26	low	17.3	14.4	20.2	15.1
				85	high	150.1	214.1	125.2	70.5
			G. Mexico	26	low	18.5	10.7	0.6	3.9
				85	low	41.0	28.9	12.2	10.7
paralichthys squamilentus	0.23	0.96	SE U.S.	26	low	44.1	30.9	79.9	55.3
				85	low	144.0	92.9	1045.3	478.4
			G. Mexico	26	low	14.3	10.1	34.5	33.1
				85	low	46.8	25.0	498.2	262.3
paralithodes camtschaticus	0.52	0.96	E. Bering S.	26	medium	100.6	82.5	134.4	543.7
				85	low	213.2	84.5	-99.0	1.8
paralithodes platypus	0.39	0.92	E. Bering S.	26	low	42.5	26.3	-32.3	32.1
				85	low	150.4	73.7	-90.7	12.6
paralomis multispina	0.46	0.76	West U.S.	26	high	268.5	269.8	20.9	43.1
				85	medium	1284.6	613.8	760.2	658.8
parapasiphaea sulcatifrons	0.57	0.91	E. Canada	26	low	160.6	118.0	-0.2	20.7
				85	low	424.6	188.9	6.3	34.0
parapenaeus politus	0.37	0.89	G. Mexico	26	medium	27.7	22.8	87.2	82.7
				85	medium	49.2	33.5	763.0	496.1
parastichopus californicus	0.35	0.90	West U.S.	26	high	493.4	498.1	68.3	87.9
				85	low	1611.3	528.5	392.8	275.8
pareques iwamotoi	0.35	0.92	G. Mexico	26	medium	56.5	60.1	56.1	67.9
				85	low	182.6	116.8	69.0	153.1
pareques umbrosus	0.33	0.96	SE U.S.	26	medium	41.1	42.3	143.0	126.7
				85	medium	93.5	61.7	981.2	757.7
			G. Mexico	26	medium	20.9	24.8	3.5	10.0
				85	low	49.6	41.2	-11.7	23.8
parmaturus xaniurus	0.48	0.93	West U.S.	26	medium	319.6	308.6	18.1	48.4
				85	low	1422.8	524.0	27.7	85.9
parophrys vetulus	0.53	0.88	West U.S.	26	low	270.7	208.4	36.6	26.0
				85	low	1276.1	346.1	158.0	71.6
parthenopoides massena	0.35	0.90	G. Mexico	26	medium	53.7	64.7	19.9	46.2
				85	medium	316.9	165.7	-69.0	38.8
pasiphaea multidentata	0.34	0.81	NE U.S.	26	low	151.9	130.2	21.9	63.2
				85	low	759.8	215.7	16.0	114.4
pasiphaea pacifica	0.29	0.14	G. Alaska	26	low	142.7	105.2	15.8	16.2
				85	low	619.9	189.7	80.0	34.0
pasiphaea tarda	0.51	0.93	West U.S.	26	medium	239.5	167.0	32.8	39.5
				85	high	715.1	378.9	607.6	404.6

**Species**	**devPA**	**devBiom**	**Region**	**RCP**	**Uncert.**	**Shift**	**sd_shift**	**%Hab.**	**sd_hab**
patinopecten caurinus	0.23	0.13	G. Alaska	26	low	170.5	92.3	31.7	25.9
				85	low	574.1	147.2	214.1	93.0
peprilus burti	0.56	0.88	G. Mexico	26	low	2.0	1.4	27.2	34.9
				85	low	3.6	2.6	19.5	68.9
peprilus paru	0.49	0.95	SE U.S.	26	medium	16.7	12.5	0.3	10.6
				85	medium	138.4	107.2	20.3	32.5
			G. Mexico	26	low	16.5	8.9	2.9	5.1
				85	medium	22.1	10.3	1.8	13.0
peprilus simillimus	0.47	0.94	West U.S.	26	low	216.3	163.7	96.9	105.3
				85	low	880.5	324.8	781.2	675.2
peprilus triacanthus	0.48	0.39	NE U.S.	26	medium	137.5	115.4	29.7	27.1
				85	medium	346.1	252.7	212.2	105.6
			G. Mexico	26	low	16.9	16.2	1.0	8.4
				85	low	37.6	21.1	72.8	45.4
periphylla periphylla	0.32	0.78	West U.S.	26	high	282.8	194.5	14.5	36.9
				85	medium	481.8	375.4	270.2	160.2
peristedion miniatum	0.41	0.70	NE U.S.	26	low	103.6	96.5	81.0	82.1
				85	medium	467.6	356.8	528.6	479.3
			G. Mexico	26	medium	125.9	112.8	-69.7	16.3
				85	high	159.9	87.5	-99.4	0.5
persephona mediterranea	0.36	0.93	SE U.S.	26	low	20.1	14.7	75.6	89.2
				85	medium	65.3	31.4	314.1	330.0
			G. Mexico	26	low	29.4	23.3	16.2	14.1
				85	medium	55.8	29.8	13.6	27.9
petrochirus diogenes	0.22	0.95	SE U.S.	26	medium	15.5	10.3	99.7	85.6
				85	medium	43.4	22.2	1493.6	771.7
			G. Mexico	26	medium	18.3	10.6	53.6	45.8
				85	medium	30.0	12.4	699.9	383.6
phycis chesteri	0.43	0.83	E. Canada	26	low	87.8	57.0	10.2	15.1
				85	medium	361.0	230.1	0.2	26.6
pilumnus sayi	0.34	0.95	SE U.S.	26	low	43.4	29.9	85.3	64.9
				85	low	147.1	75.3	684.3	518.7
			G. Mexico	26	medium	21.4	13.7	32.9	27.0
				85	high	56.4	28.9	145.5	84.7
pisaster brevispinus	0.43	0.97	G. Alaska	26	low	350.3	250.5	27.3	20.0
				85	low	1523.6	265.5	172.0	86.2
pitar cordatus	0.36	0.92	G. Mexico	26	medium	40.9	23.4	39.7	62.6
				85	low	126.2	90.1	64.6	111.9

Species	devPA	devBiom	Region	RCP	Uncert.	Shift	sd_shift	%Hab.	sd_hab
placopecten magellanicus	0.31	0.79	NE U.S.	26	high	362.3	305.1	-7.1	31.0
				85	high	721.6	445.1	-38.3	32.8
platichthys stellatus	0.39	0.11	G. Alaska	26	medium	172.8	141.6	28.2	33.3
				85	medium	737.3	412.5	132.2	126.4
platymera gaudichaudii	0.42	0.91	West U.S.	26	low	173.8	111.1	34.3	22.5
				85	low	815.4	376.0	143.2	113.5
pleurobranchaea californica	0.53	0.91	West U.S.	26	low	231.6	165.0	38.9	35.3
				85	low	996.6	354.4	149.0	123.5
pleurogrammus monopterygius	0.43	0.88	E. Bering S.	26	medium	140.7	100.6	-37.0	48.8
				85	low	284.2	97.0	-94.2	15.2
pleuronectes quadrituberculatus	0.70	0.95	E. Bering S.	26	medium	94.5	71.4	1.9	173.3
				85	medium	183.0	71.1	-97.5	5.0
pleuronichthys decurrens	0.46	0.97	West U.S.	26	low	250.8	206.1	19.9	13.9
				85	low	1258.7	332.5	96.4	70.1
pleuronichthys verticalis	0.46	0.95	West U.S.	26	low	98.8	90.8	69.0	39.3
				85	medium	659.7	367.1	321.6	224.0
plicifusus kroeyeri	0.35	0.97	E. Bering S.	26	low	63.5	41.4	-34.6	66.3
				85	low	136.9	62.9	-97.3	5.8
podochela sidneyi	0.28	0.98	SE U.S.	26	medium	36.5	36.0	135.1	113.2
				85	low	105.0	62.0	1141.1	653.3
			G. Mexico	26	high	14.0	12.6	21.4	17.9
				85	high	28.9	23.9	123.2	44.8
pododesmus macrochisma	0.24	0.94	E. Bering S.	26	medium	90.0	58.7	-39.5	44.9
				85	low	197.6	89.2	-94.1	13.2
podothecus accipenserinus	0.59	0.28	E. Bering S.	26	low	23.6	14.5	-1.2	13.4
				85	low	86.8	25.8	-37.6	29.5
pogonias cromis	0.27	0.97	SE U.S.	26	medium	53.7	60.7	-4.7	9.1
				85	high	817.7	618.0	213.6	287.2
			G. Mexico	26	low	10.3	8.7	-3.1	4.5
				85	low	25.3	14.8	-2.6	13.1
pollachius virens	0.39	0.90	E. Canada	26	low	134.6	76.0	11.9	16.0
				85	medium	566.6	312.8	-8.2	39.4
polyacanthonotus rissoanus	0.52	0.92	E. Canada	26	low	145.5	88.2	5.3	15.2
				85	low	532.0	236.6	-8.0	34.1
polydactylus octoemus	0.35	0.86	G. Mexico	26	medium	21.1	15.2	42.8	31.5
				85	low	51.1	32.0	394.6	138.1

Species	devPA	devBiom	Region	RCP	Uncert.	Shift	sd_shift	%Hab.	sd_hab
polymixia lowei	0.44	0.69	SE U.S.	26	medium	117.5	97.2	12.6	12.3
				85	medium	658.4	343.8	94.0	74.6
			G. Mexico	26	low	101.6	62.6	-4.0	8.7
				85	low	177.5	75.9	-29.3	12.0
polystira albida	0.36	0.91	G. Mexico	26	high	49.7	29.9	40.1	52.2
				85	medium	106.9	97.7	55.4	117.3
pomatomus saltatrix	0.43	0.84	SE U.S.	26	low	148.5	132.2	21.9	90.7
				85	low	582.0	226.4	358.0	299.0
			G. Mexico	26	medium	26.4	16.1	-26.2	15.3
				85	low	73.7	44.9	-72.0	13.1
pontinus longispinis	0.44	0.87	SE U.S.	26	low	14.7	11.6	195.2	158.2
				85	low	53.8	38.7	1174.6	837.5
			G. Mexico	26	low	41.7	39.5	29.9	29.1
				85	low	118.1	59.5	54.6	56.5
pontophilus norvegicus	0.34	0.90	E. Canada	26	low	81.3	52.7	10.3	14.1
				85	medium	246.5	151.6	-5.8	18.8
poraniopsis inflata	0.19	0.91	West U.S.	26	low	280.2	227.1	33.1	51.7
				85	low	1315.8	362.6	142.9	124.0
porichthys notatus	0.50	0.90	West U.S.	26	low	215.3	125.3	39.6	34.9
				85	low	1036.4	406.4	119.5	122.1
porichthys plectrodon	0.43	0.95	SE U.S.	26	medium	32.5	22.7	101.7	80.0
				85	low	113.1	54.4	308.1	342.0
			G. Mexico	26	low	58.2	50.4	-9.7	11.4
				85	medium	170.3	90.5	-72.7	18.6
portunus gibbesii	0.49	0.94	SE U.S.	26	medium	28.7	33.3	106.6	100.1
				85	low	68.8	37.7	868.0	600.2
			G. Mexico	26	medium	17.7	13.6	13.4	9.0
				85	medium	43.4	33.0	54.0	18.2
priacanthus arenatus	0.28	0.93	SE U.S.	26	medium	38.2	34.9	65.6	69.9
				85	low	121.8	67.0	350.7	348.0
			G. Mexico	26	medium	36.1	24.6	-22.4	15.0
				85	medium	98.2	61.4	-77.3	16.4
prionotus alatus	0.31	0.86	SE U.S.	26	high	23.7	42.6	1757.4	2993.2
				85	medium	138.5	103.8	1.1E+05	1.9E+05
			G. Mexico	26	medium	36.5	29.3	212.5	196.8
				85	medium	58.4	48.6	5278.2	3762.6

Species	devPA	devBiom	Region	RCP	Uncert.	Shift	sd_shift	%Hab.	sd_hab
prionotus carolinus	0.41	0.74	SE U.S.	26	low	209.6	156.5	19.6	53.2
				85	low	594.9	245.1	352.1	169.3
			G. Mexico	26	low	42.6	26.7	-40.7	19.3
				85	low	105.3	44.8	-92.1	3.4
prionotus evolans	0.38	0.83	NE U.S.	26	medium	132.5	149.0	181.5	335.9
				85	high	332.2	311.9	1822.5	2413.8
prionotus longispinosus	0.56	0.88	SE U.S.	26	medium	52.5	66.4	180.3	235.6
				85	low	271.2	119.2	801.3	731.8
			G. Mexico	26	medium	1.4	1.5	2.7	23.6
				85	medium	2.7	3.1	-50.6	35.2
prionotus martis	0.45	0.91	G. Mexico	26	high	13.4	13.4	76.8	68.2
				85	low	34.8	25.0	421.7	244.5
prionotus ophryas	0.34	0.97	SE U.S.	26	medium	31.4	23.4	109.9	91.5
				85	low	111.2	47.1	1000.9	635.0
			G. Mexico	26	medium	17.0	12.0	27.8	22.1
				85	high	47.0	27.8	57.8	48.3
prionotus paralatus	0.52	0.90	SE U.S.	26	medium	79.5	101.0	87.3	100.2
				85	low	387.0	150.8	297.7	734.2
			G. Mexico	26	high	1.9	3.4	-18.4	36.6
				85	high	10.0	29.0	-93.0	9.6
prionotus roseus	0.37	0.97	SE U.S.	26	medium	15.3	9.8	91.8	79.5
				85	high	35.7	22.7	1822.5	995.1
			G. Mexico	26	medium	18.8	12.0	53.4	48.6
				85	low	42.6	28.7	859.2	463.0
prionotus rubio	0.33	0.95	SE U.S.	26	medium	9.2	5.1	13.1	12.4
				85	low	48.3	30.0	46.5	22.1
			G. Mexico	26	medium	12.7	13.2	-6.9	5.2
				85	high	40.0	27.8	-40.3	13.7
prionotus scitulus	0.44	0.93	SE U.S.	26	medium	15.7	19.3	-7.8	13.1
				85	high	434.7	429.1	-3.2	68.0
			G. Mexico	26	medium	27.3	22.8	-23.3	19.2
				85	medium	79.2	39.9	-82.3	14.1
prionotus stearnsi	0.54	0.89	SE U.S.	26	medium	82.7	104.5	66.7	87.9
				85	low	369.2	160.3	300.4	870.6
			G. Mexico	26	low	0.4	0.4	-8.1	44.2
				85	high	1.5	2.4	-91.7	11.1

Species	devPA	devBiom	Region	RCP	Uncert.	Shift	sd_shift	%Hab.	sd_hab
prionotus tribulus	0.32	0.95	SE U.S.	26	low	9.9	7.3	18.2	14.4
				85	low	66.1	52.1	70.9	26.3
			G. Mexico	26	medium	16.8	18.0	-2.2	4.1
				85	medium	51.4	45.8	-31.2	14.0
pristigenys alta	0.25	0.92	SE U.S.	26	high	22.4	17.1	197.0	203.0
				85	high	50.8	42.5	12873.8	13755.2
			G. Mexico	26	medium	20.2	12.5	141.2	147.5
				85	medium	31.7	23.3	9417.5	11106.2
pristipomoides aquilonaris	0.57	0.89	SE U.S.	26	high	71.7	93.9	81.3	93.5
				85	low	333.0	154.0	307.3	830.0
			G. Mexico	26	low	0.7	0.6	-12.0	34.2
				85	low	2.0	2.0	-88.2	13.5
pseudarchaster parelii	0.13	0.24	G. Alaska	26	low	128.9	76.4	7.2	7.2
				85	low	505.7	132.3	29.5	8.8
pseudopleuronectes americanus	0.41	0.87	NE U.S.	26	low	96.3	83.3	-18.9	24.8
				85	medium	394.4	235.1	-68.9	29.5
pseudostichopus mollis	0.33	0.38	G. Alaska	26	low	241.8	184.6	7.3	20.9
				85	low	876.2	351.5	60.9	56.2
psolus squamatus	0.31	0.13	G. Alaska	26	medium	52.9	28.0	-4.0	8.4
				85	medium	206.2	120.9	28.4	31.7
pteraster jordani	0.34	0.92	G. Alaska	26	low	274.0	208.1	17.3	36.9
				85	medium	1295.7	451.9	107.5	66.9
pteraster militaris	0.24	0.08	E. Bering S.	26	medium	72.8	52.6	-9.3	9.8
				85	low	228.9	101.1	-23.6	15.2
pteraster obscurus	0.38	0.95	E. Bering S.	26	low	47.7	27.0	-44.5	42.2
				85	low	161.1	70.7	-97.4	5.4
ptilosarcus gurneyi	0.17	0.88	West U.S.	26	low	293.7	163.8	40.7	25.1
				85	low	1445.1	423.1	141.3	102.4
pycnopodia helianthoides	0.27	0.14	G. Alaska	26	low	105.2	83.7	19.8	12.4
				85	low	389.1	145.9	67.4	24.9
pyrosoma atlanticum	0.35	0.66	West U.S.	26	medium	7.7	5.8	66.5	36.4
				85	medium	81.0	69.2	219.6	227.6
pyrulofusus deformis	0.27	0.97	E. Bering S.	26	medium	97.7	76.6	-14.8	117.4
				85	high	352.2	410.5	-98.4	3.4
pyrulofusus melonis	0.30	0.86	E. Bering S.	26	medium	86.9	74.1	-29.7	93.2
				85	low	183.2	90.1	-99.3	1.6

**Species**	**devPA**	**devBiom**	**Region**	**RCP**	**Uncert.**	**Shift**	**sd_shift**	**%Hab.**	**sd_hab**
rachycentron canadum	0.22	0.97	SE U.S.	26	low	9.9	8.1	21.8	15.4
				85	medium	128.6	124.1	91.8	32.4
			G. Mexico	26	medium	29.7	29.4	9.4	11.8
				85	low	129.4	75.2	-40.3	26.3
raja binoculata	0.27	0.13	G. Alaska	26	low	128.2	89.5	22.8	16.7
				85	low	428.0	95.5	86.0	28.7
raja eglanteria	0.49	0.93	SE U.S.	26	medium	65.3	63.3	-17.2	12.5
				85	low	689.7	250.5	4.3	57.0
			G. Mexico	26	high	22.4	23.4	-34.5	21.8
				85	low	77.9	51.5	-86.7	9.9
raja inornata	0.50	0.93	West U.S.	26	low	108.3	46.7	95.3	74.9
				85	medium	208.9	112.6	984.6	1487.8
raja rhina	0.42	0.22	G. Alaska	26	low	205.4	139.2	17.4	16.1
				85	low	675.3	178.3	31.8	35.0
raja texana	0.34	0.90	G. Mexico	26	high	49.3	64.4	50.5	56.8
				85	medium	200.8	159.8	-2.7	82.3
rajella fyllae	0.33	0.84	E. Canada	26	low	87.0	65.3	1.9	19.3
				85	low	182.6	72.6	-36.1	28.9
raninoides louisianensis	0.42	0.91	G. Mexico	26	high	38.5	23.1	22.7	28.4
				85	medium	105.7	87.8	22.6	62.2
rathbunaster californicus	0.36	0.95	G. Alaska	26	medium	307.7	286.2	10.1	12.2
				85	low	1395.5	305.8	36.8	52.5
reilla mulleri	0.43	0.90	G. Mexico	26	medium	33.1	20.7	221.6	283.2
				85	medium	96.3	61.4	10746.4	16003.4
reinhardtius hippoglossoides	0.71	0.87	E. Canada	26	low	83.6	61.4	-7.5	17.7
				85	low	283.4	137.7	-53.5	33.1
	0.50	0.96	E. Bering S.	26	low	33.8	27.6	-41.1	41.8
				85	low	160.4	83.2	-95.1	9.8
rhinoptera bonasus	0.32	0.96	SE U.S.	26	low	21.6	16.2	-1.3	9.8
				85	medium	292.9	213.6	38.8	45.7
			G. Mexico	26	medium	23.7	25.5	-4.9	8.6
				85	medium	92.2	65.2	-58.8	17.9
rhizoprionodon terraenovae	0.49	0.98	SE U.S.	26	low	16.6	12.3	-2.2	9.0
				85	low	149.9	103.8	-16.6	11.8
			G. Mexico	26	low	23.4	21.4	-9.6	10.1
				85	low	109.1	65.7	-69.3	15.3

Species	devPA	devBiom	Region	RCP	Uncert.	Shift	sd_shift	%Hab.	sd_hab
rhomboplites aurorubens	0.38	0.87	SE U.S.	26	high	19.4	32.0	721.3	1089.7
				85	medium	253.6	178.7	10474.9	11497.3
			G. Mexico	26	medium	27.0	28.5	76.2	70.7
				85	medium	75.7	64.1	312.0	215.6
rhynchoconger flavus	0.42	0.90	G. Mexico	26	medium	81.2	84.8	-5.0	22.0
				85	medium	284.5	123.4	-82.1	22.7
rimapenaeus constrictus	0.40	0.87	SE U.S.	26	low	17.7	11.3	64.4	84.7
				85	high	156.6	199.8	356.4	502.3
sabinea hystrix	0.54	0.89	E. Canada	26	low	175.0	98.3	-8.1	12.9
				85	low	524.5	214.0	-11.1	44.4
sabinea sarsii	0.28	0.85	E. Canada	26	low	124.1	66.8	-2.1	15.7
				85	low	271.3	88.9	-53.2	33.1
sabinea septemcarinata	0.50	0.87	E. Canada	26	medium	83.9	74.6	-18.4	25.8
				85	low	173.5	93.9	-75.2	26.0
sagenaster evermanni	0.49	0.78	West U.S.	26	high	164.7	151.8	40.5	70.7
				85	high	510.7	355.5	653.7	404.1
sardinella aurita	0.30	0.91	SE U.S.	26	low	11.5	9.5	25.0	23.9
				85	medium	104.9	112.6	78.8	43.7
			G. Mexico	26	low	10.0	6.7	0.5	4.2
				85	low	39.2	26.9	-24.1	14.5
sardinops sagax	0.30	0.94	G. Alaska	26	low	327.6	216.3	35.7	28.2
				85	low	1277.3	315.3	163.2	82.6
sarritor frenatus	0.28	0.92	E. Bering S.	26	low	76.3	53.4	-39.6	41.9
				85	low	221.0	71.4	-95.4	9.6
saurida brasiliensis	0.50	0.86	SE U.S.	26	medium	48.2	62.3	59.3	62.1
				85	low	328.0	152.2	165.0	214.0
			G. Mexico	26	low	3.0	2.4	-15.8	25.1
				85	low	5.4	3.7	-85.8	11.9
sclerocrangon boreas	0.49	0.85	E. Canada	26	medium	108.5	85.0	-18.4	34.9
				85	medium	191.3	121.9	-76.9	29.9
scomber japonicus	0.20	0.93	SE U.S.	26	high	21.0	12.1	28.6	30.2
				85	low	156.4	104.7	168.3	114.9
			G. Mexico	26	low	62.2	55.0	-11.5	14.9
				85	medium	181.9	80.5	-84.0	16.6
	0.38	0.91	West U.S.	26	low	310.2	222.7	38.1	29.7
				85	low	1604.3	364.9	189.9	116.6
scomber scombrus	0.27	0.77	NE U.S.	26	low	66.2	49.3	-0.1	16.6
				85	medium	314.9	266.1	-54.6	18.8

Species	devPA	devBiom	Region	RCP	Uncert.	Shift	sd_shift	%Hab.	sd_hab
scomberesox saurus	0.22	0.73	E. Canada	26	low	60.3	54.2	456.5	955.4
				85	medium	278.2	236.1	1885.2	2785.5
scomberomorus cavalla	0.39	0.96	SE U.S.	26	low	20.2	16.6	29.2	22.6
				85	medium	157.6	131.4	120.8	57.7
			G. Mexico	26	medium	15.9	17.2	5.4	7.2
				85	low	56.8	44.8	-27.3	19.8
scomberomorus maculatus	0.48	0.98	SE U.S.	26	low	16.8	13.1	4.0	9.4
				85	medium	265.5	242.7	40.7	45.6
			G. Mexico	26	medium	18.5	18.9	-3.0	7.9
				85	medium	76.0	61.9	-51.0	18.1
scophthalmus aquosus	0.42	0.83	NE U.S.	26	medium	46.9	57.1	-12.7	20.2
				85	high	261.7	323.6	-55.7	25.2
scorpaena brasiliensis	0.43	0.96	SE U.S.	26	medium	88.1	89.4	302.5	336.2
				85	low	349.8	168.7	11383.7	12751.2
			G. Mexico	26	medium	21.7	23.3	42.6	39.8
				85	low	77.6	43.3	124.7	93.3
scorpaena calcarata	0.39	0.96	SE U.S.	26	low	53.4	42.1	170.3	143.5
				85	low	200.7	94.0	1304.3	1228.8
			G. Mexico	26	low	27.5	22.6	32.0	32.2
				85	medium	116.0	68.5	-9.9	50.9
scyliorhinus retifer	0.48	0.79	NE U.S.	26	high	112.5	152.1	132.8	199.6
				85	medium	595.9	444.5	1900.1	2811.5
scyllarides nodifer	0.37	0.85	SE U.S.	26	high	30.0	50.5	821.5	1266.3
				85	medium	281.9	208.3	17446.4	18427.5
			G. Mexico	26	medium	17.4	21.9	71.6	73.1
				85	low	47.4	39.5	371.3	260.0
scyllarus chacei	0.44	0.98	SE U.S.	26	medium	71.7	55.6	149.9	120.3
				85	low	244.1	132.8	1954.6	1591.7
			G. Mexico	26	medium	18.7	14.1	49.2	36.4
				85	medium	53.0	32.0	211.8	87.0
sebastes aleutianus	0.31	0.82	E. Bering S.	26	medium	120.3	116.7	-34.1	31.5
				85	low	563.0	153.7	-91.5	12.8
sebastes alutus	0.38	0.22	G. Alaska	26	low	203.0	121.4	1.0	6.7
				85	low	768.1	136.9	7.0	15.2
sebastes aurora	0.43	0.97	G. Alaska	26	low	228.8	195.3	1.0	17.7
				85	medium	1237.7	395.9	-0.3	43.2
sebastes babcocki	0.32	0.88	G. Alaska	26	low	383.7	269.4	11.4	14.7
				85	low	1273.8	254.6	11.6	39.2

Species	devPA	devBiom	Region	RCP	Uncert.	Shift	sd_shift	%Hab.	sd_hab
sebastes borealis	0.40	0.86	G. Alaska	26	low	292.5	199.6	15.2	19.2
				85	medium	907.5	264.3	20.4	35.1
sebastes brevispinis	0.34	0.88	G. Alaska	26	low	395.9	285.6	17.1	24.1
				85	medium	1406.1	287.1	81.5	63.5
sebastes chlorostictus	0.29	0.88	West U.S.	26	low	196.9	124.2	24.6	15.5
				85	low	957.4	367.7	74.6	68.3
sebastes crameri	0.44	0.92	G. Alaska	26	low	257.7	196.6	7.7	13.5
				85	low	1213.0	325.7	13.4	47.5
sebastes diploproa	0.46	0.93	G. Alaska	26	low	290.0	242.8	2.2	12.1
				85	medium	1312.4	298.0	-2.6	37.1
sebastes elongatus	0.42	0.94	G. Alaska	26	low	294.2	198.2	10.6	20.1
				85	low	1294.8	320.5	52.9	54.5
sebastes entomelas	0.28	0.95	G. Alaska	26	low	337.3	301.8	9.1	15.2
				85	low	1447.5	260.5	50.3	47.0
sebastes fasciatus	0.52	0.73	NE U.S.	26	medium	57.4	73.3	12.5	66.2
				85	medium	809.2	393.4	-45.2	65.2
sebastes flavidus	0.36	0.09	E. Bering S.	26	low	78.2	65.6	-3.7	11.7
				85	low	227.4	127.5	-28.8	20.5
sebastes goodei	0.42	0.87	West U.S.	26	medium	234.5	214.0	1.3	13.9
				85	high	861.5	317.9	80.3	196.2
sebastes helvomaculatus	0.28	0.94	G. Alaska	26	low	306.4	225.1	12.1	25.5
				85	low	1423.2	357.3	80.3	62.1
sebastes jordani	0.38	0.88	West U.S.	26	low	158.5	110.1	18.7	12.8
				85	medium	736.2	434.5	64.9	89.8
sebastes melanostictus	0.28	0.85	E. Bering S.	26	low	88.1	79.0	-35.7	35.8
				85	medium	447.7	152.3	-95.6	6.2
sebastes melanostomus	0.38	0.90	West U.S.	26	medium	308.4	285.4	6.8	20.1
				85	low	1652.7	666.3	13.2	34.9
sebastes mentella	0.61	0.80	E. Canada	26	low	99.6	57.8	-8.3	28.2
				85	medium	262.2	156.4	-61.3	33.8
sebastes paucispinis	0.34	0.95	G. Alaska	26	medium	339.3	373.6	11.8	13.6
				85	low	1587.8	313.5	68.8	60.7
sebastes pinniger	0.34	0.95	G. Alaska	26	low	318.3	230.3	17.4	20.1
				85	low	1343.7	300.4	92.7	51.1
sebastes polyspinis	0.37	0.85	E. Bering S.	26	low	90.5	64.4	-28.6	56.1
				85	low	166.6	51.1	-96.9	6.7
sebastes proriger	0.27	0.84	G. Alaska	26	low	407.3	330.3	10.5	26.0
				85	medium	1714.0	324.5	130.6	90.5

Species	devPA	devBiom	Region	RCP	Uncert.	Shift	sd_shift	%Hab.	sd_hab
sebastes ruberrimus	0.17	0.86	G. Alaska	26	low	253.5	227.3	24.8	26.8
				85	medium	718.6	419.6	122.4	91.8
sebastes saxicola	0.42	0.92	G. Alaska	26	medium	299.4	315.8	7.1	19.5
				85	low	1084.2	346.4	31.0	54.5
sebastes semicinctus	0.47	0.92	West U.S.	26	low	206.5	149.2	55.9	44.6
				85	medium	644.2	363.4	355.2	251.6
sebastes variabilis	0.26	0.80	E. Bering S.	26	low	274.3	175.2	16.8	49.8
				85	low	733.9	201.5	-8.7	123.1
sebastes variegatus	0.32	0.04	G. Alaska	26	low	67.5	40.6	3.3	4.6
				85	low	266.6	83.9	22.7	10.5
sebastes wilsoni	0.19	0.91	G. Alaska	26	low	300.1	185.7	27.5	29.2
				85	low	1261.7	355.8	110.7	85.6
sebastes zacentrus	0.29	0.83	G. Alaska	26	low	447.1	368.5	18.0	26.0
				85	low	1614.5	379.2	92.0	49.6
sebastolobus alascanus	0.50	0.89	West U.S.	26	high	217.6	157.3	16.7	22.0
				85	high	682.0	268.0	144.7	109.6
sebastolobus altivelis	0.69	0.96	G. Alaska	26	high	242.2	222.5	-6.4	17.9
				85	medium	1075.6	405.8	124.2	97.7
selar crumenophthalmus	0.23	0.91	SE U.S.	26	low	14.3	9.1	25.5	15.0
				85	medium	99.0	110.6	135.5	58.0
			G. Mexico	26	medium	19.3	18.6	-0.1	6.6
				85	low	50.1	30.6	-19.9	17.0
selene setapinnis	0.42	0.90	SE U.S.	26	low	17.3	13.0	34.6	20.9
				85	high	162.3	177.2	223.9	120.2
			G. Mexico	26	medium	10.5	5.6	6.4	8.7
				85	medium	33.0	17.4	22.6	15.8
selene vomer	0.42	0.95	SE U.S.	26	low	11.6	7.3	48.0	37.0
				85	low	63.8	44.3	257.8	128.4
			G. Mexico	26	medium	15.4	16.6	9.6	9.2
				85	low	47.7	40.6	0.3	20.0
sergestes arcticus	0.50	0.85	E. Canada	26	low	123.8	102.8	2.8	14.5
				85	low	421.8	154.5	-32.3	25.8
sergia robusta	0.53	0.90	E. Canada	26	medium	151.2	127.7	14.3	22.6
				85	low	543.4	220.1	67.5	123.2
seriola dumerili	0.20	0.92	SE U.S.	26	high	67.9	103.7	445.4	520.7
				85	low	377.0	170.2	19592.7	27935.6
			G. Mexico	26	medium	52.5	52.0	56.3	64.5
				85	medium	252.5	162.1	9.9	124.2

Species	devPA	devBiom	Region	RCP	Uncert.	Shift	sd_shift	%Hab.	sd_hab
serranus atrobranchus	0.59	0.89	SE U.S.	26	medium	72.6	91.8	112.9	124.0
				85	low	300.9	131.1	748.3	1214.3
			G. Mexico	26	medium	41.7	52.9	13.1	29.1
				85	medium	101.4	110.1	-45.3	40.6
serranus phoebe	0.40	0.85	SE U.S.	26	high	27.4	59.1	1191.4	1650.4
				85	medium	151.7	141.3	2.1E+05	2.0E+05
			G. Mexico	26	high	36.0	19.6	464.5	541.9
				85	low	73.0	56.9	1.3E+05	2.0E+05
serratiflustra serrulata	0.23	0.95	E. Bering S.	26	medium	50.2	30.9	-35.0	68.4
				85	low	111.8	50.4	-99.0	2.1
serripes groenlandicus	0.18	0.94	E. Bering S.	26	low	43.0	27.2	-42.1	46.0
				85	low	109.5	48.4	-96.9	6.0
serrivomer beanii	0.60	0.91	E. Canada	26	low	156.1	105.1	3.8	18.6
				85	low	266.8	132.0	3.2	31.2
sicyonia brevirostris	0.43	0.94	SE U.S.	26	low	62.1	50.5	156.3	151.4
				85	low	188.4	78.7	2028.4	1625.2
			G. Mexico	26	low	66.1	62.3	3.4	18.5
				85	medium	259.7	140.3	-71.1	24.6
sicyonia burkenroadi	0.32	0.91	G. Mexico	26	medium	90.4	91.1	5.7	31.2
				85	medium	295.9	146.6	-76.1	30.3
sicyonia dorsalis	0.45	0.88	G. Mexico	26	low	62.2	58.3	16.0	29.4
				85	low	139.1	97.3	-47.8	39.2
siliqua alta	0.44	0.97	E. Bering S.	26	low	21.5	19.3	-26.0	28.4
				85	high	95.0	132.6	-87.8	14.4
solaster endeca	0.23	0.79	E. Canada	26	medium	127.6	110.8	-16.5	36.6
				85	high	294.1	272.7	-72.8	20.6
sphoeroides dorsalis	0.44	0.95	SE U.S.	26	medium	51.7	58.0	385.0	442.1
				85	low	234.5	109.7	9517.5	8929.8
			G. Mexico	26	low	24.2	20.0	52.2	47.8
				85	medium	58.0	44.1	263.2	149.0
sphoeroides maculatus	0.43	0.84	SE U.S.	26	medium	166.3	196.3	-13.7	21.9
				85	low	770.0	232.8	180.9	390.5
sphoeroides parvus	0.44	0.87	G. Mexico	26	medium	1.0	1.2	24.7	44.5
				85	medium	2.5	2.9	-24.2	57.0
sphoeroides spengleri	0.43	0.97	SE U.S.	26	high	48.6	74.9	283.3	244.1
				85	low	353.2	171.2	5945.9	6731.5
			G. Mexico	26	medium	25.3	25.2	111.5	89.6
				85	low	111.1	55.0	576.9	311.7

Species	devPA	devBiom	Region	RCP	Uncert.	Shift	sd_shift	%Hab.	sd_hab
sphyraena borealis	0.43	0.90	SE U.S.	26	low	12.2	6.5	44.9	31.9
				85	low	57.5	26.3	253.7	119.8
			G. Mexico	26	low	16.9	13.1	8.6	7.5
				85	low	36.8	25.3	7.4	16.2
sphyraena guachancho	0.33	0.96	SE U.S.	26	medium	19.7	15.8	88.1	70.1
				85	low	72.1	33.8	828.2	461.0
			G. Mexico	26	medium	32.9	33.2	53.7	38.5
				85	low	78.1	53.7	196.0	91.1
sphyrna tiburo	0.49	0.98	SE U.S.	26	low	18.3	13.7	35.8	29.9
				85	medium	82.3	94.3	127.0	114.5
			G. Mexico	26	medium	19.7	15.4	-15.4	11.2
				85	medium	47.2	32.3	-60.1	15.0
spirontocaris lilljeborgi	0.26	0.80	E. Canada	26	low	116.4	67.6	-10.3	16.1
				85	low	285.5	133.9	-52.7	41.6
spirontocaris spinus	0.51	0.84	E. Canada	26	low	141.4	87.3	-19.5	26.1
				85	medium	273.9	118.3	-74.3	28.2
squalus acanthias	0.41	0.91	E. Canada	26	low	96.4	71.5	2.6	8.1
				85	low	483.5	212.7	-31.6	24.4
	0.30	0.23	G. Alaska	26	low	501.4	374.8	199.7	585.0
				85	medium	1397.9	380.2	432.1	284.1
squalus suckleyi	0.38	0.63	West U.S.	26	low	194.2	153.5	34.8	19.7
				85	medium	646.0	355.1	139.4	153.8
squatina dumeril	0.31	0.94	SE U.S.	26	low	33.9	27.5	9.0	25.1
				85	low	65.4	45.3	87.9	43.1
			G. Mexico	26	medium	41.0	36.8	-15.4	15.0
				85	medium	127.8	79.2	-76.7	16.3
squilla chydaea	0.45	0.91	G. Mexico	26	medium	43.7	47.2	64.8	70.4
				85	low	145.0	96.4	256.3	235.7
squilla empusa	0.44	0.95	SE U.S.	26	low	52.1	44.5	189.9	191.7
				85	low	122.7	64.3	2853.2	2201.0
			G. Mexico	26	low	17.5	13.5	32.1	27.2
				85	low	55.2	19.5	131.2	68.3
stegophiura ponderosa	0.31	0.82	G. Alaska	26	low	278.9	232.5	13.1	23.2
				85	medium	900.9	436.8	64.7	43.4
steindachneria argentea	0.53	0.90	G. Mexico	26	medium	9.7	6.3	54.1	44.6
				85	high	15.0	8.8	580.6	303.2

Species	devPA	devBiom	Region	RCP	Uncert.	Shift	sd_shift	%Hab.	sd_hab
stellifer lanceolatus	0.43	0.97	SE U.S.	26	low	16.3	14.3	48.0	43.6
				85	low	89.4	52.8	247.7	180.8
			G. Mexico	26	high	20.6	11.7	0.2	9.2
				85	high	69.3	68.7	-47.9	27.2
stenobrachius leucopsarus	0.19	0.83	West U.S.	26	medium	218.8	206.8	22.5	18.9
				85	low	1124.1	274.8	81.6	34.9
stenocionops furcatus	0.37	0.91	G. Mexico	26	medium	40.6	35.7	136.0	136.9
				85	medium	98.4	80.0	986.5	807.7
stenorhynchus seticornis	0.36	0.98	SE U.S.	26	low	111.3	101.9	245.0	284.8
				85	medium	324.3	207.3	3430.0	3304.0
			G. Mexico	26	medium	38.2	44.7	16.6	22.2
				85	medium	149.3	94.4	-18.5	44.0
stenotomus caprinus	0.67	0.89	G. Mexico	26	high	14.8	22.6	-2.8	26.0
				85	medium	34.9	44.2	-72.9	20.5
stenotomus chrysops	0.50	0.75	NE U.S.	26	medium	183.8	203.2	43.0	100.2
				85	high	338.8	433.2	950.4	928.9
stephanolepis hispidus	0.34	0.89	SE U.S.	26	low	15.9	12.9	73.9	58.9
				85	low	48.1	19.7	637.7	306.9
			G. Mexico	26	low	9.2	6.8	25.7	19.7
				85	medium	12.7	8.4	174.3	56.3
stereomastis sculpta	0.61	0.89	E. Canada	26	low	132.5	72.8	3.0	13.5
				85	low	365.8	129.8	-16.9	26.5
stomias ferox	0.62	0.85	E. Canada	26	medium	139.3	115.6	4.3	14.3
				85	low	410.9	140.6	-12.6	32.4
stomolophus meleagris	0.58	0.94	SE U.S.	26	medium	22.1	16.4	122.5	128.8
				85	medium	91.9	95.9	2586.9	3021.9
			G. Mexico	26	medium	43.8	31.5	61.3	65.2
				85	high	145.1	152.7	1317.0	1393.6
stomphia coccinea	0.17	0.89	E. Bering S.	26	medium	49.7	39.2	-46.5	51.0
				85	low	109.2	49.1	-98.7	2.9
strongylocentrotus droebachiensis	0.34	0.81	E. Canada	26	medium	173.3	162.5	-1.6	120.9
				85	medium	872.6	462.4	-80.3	26.8
strongylocentrotus fragilis	0.32	0.30	G. Alaska	26	low	144.2	90.2	17.9	15.5
				85	low	461.3	140.5	85.2	30.3
strongylocentrotus polyacanthus	0.27	0.94	E. Bering S.	26	low	95.8	51.5	-30.6	51.2
				85	low	235.7	86.0	-89.6	18.9
styela rustica	0.33	0.88	E. Bering S.	26	medium	96.1	92.9	-40.8	53.8
				85	low	225.2	89.1	-97.0	7.3

Species	devPA	devBiom	Region	RCP	Uncert.	Shift	sd_shift	%Hab.	sd_hab
stylasterias forreri	0.23	0.86	West U.S.	26	low	327.9	245.1	27.1	36.5
				85	low	1347.2	300.0	108.2	76.3
syacium gunteri	0.52	0.88	SE U.S.	26	high	20.0	34.9	262.3	303.8
				85	low	239.5	148.9	2072.8	1481.9
			G. Mexico	26	low	1.7	1.3	25.7	38.1
				85	low	2.7	1.5	61.6	234.1
syacium papillosum	0.43	0.95	SE U.S.	26	medium	23.9	14.5	95.9	71.4
				85	low	90.3	34.4	674.4	418.2
			G. Mexico	26	medium	15.6	12.4	19.4	17.7
				85	medium	48.6	38.4	5.5	32.8
symphurus civitatum	0.24	0.91	SE U.S.	26	medium	15.0	19.9	228.6	207.2
				85	low	98.7	52.3	3200.0	1901.5
			G. Mexico	26	high	29.6	30.3	127.0	123.3
				85	medium	43.1	48.0	1362.1	984.8
symphurus diomedeanus	0.29	0.89	SE U.S.	26	high	41.0	55.5	231.4	294.7
				85	low	220.2	118.5	3240.6	2272.3
			G. Mexico	26	high	36.0	29.7	96.9	86.6
				85	high	50.2	58.6	934.9	538.5
symphurus plagiusa	0.30	0.96	SE U.S.	26	low	12.2	9.6	4.6	6.8
				85	low	68.5	48.5	4.7	12.2
			G. Mexico	26	high	16.2	13.8	-5.3	7.6
				85	medium	71.2	62.6	-63.0	16.8
synaphobranchus kaupii	0.71	0.88	E. Canada	26	medium	164.3	127.2	7.0	13.6
				85	low	475.8	156.2	-17.1	34.2
syngnathus fuscus	0.25	0.61	SE U.S.	26	low	69.3	48.8	53.1	65.0
				85	low	322.1	185.7	2286.5	2349.0
			G. Mexico	26	high	9.9	7.7	58.6	63.4
				85	medium	23.6	14.9	2484.8	1889.4
synodus foetens	0.58	0.96	SE U.S.	26	low	16.3	15.0	-4.5	8.4
				85	medium	144.9	125.4	5.0	23.9
			G. Mexico	26	medium	16.9	11.5	-9.1	9.0
				85	medium	75.1	51.8	-59.3	17.7
synodus intermedius	0.52	0.90	SE U.S.	26	high	27.3	39.3	837.0	1276.8
				85	medium	294.7	188.6	26651.7	35323.1
			G. Mexico	26	medium	22.2	22.1	89.4	96.8
				85	low	87.5	49.2	391.0	390.1

Species	devPA	devBiom	Region	RCP	Uncert.	Shift	sd_shift	%Hab.	sd_hab
synodus poeyi	0.40	0.85	SE U.S.	26	high	21.1	39.4	224.7	247.3
				85	low	298.6	169.1	1441.1	1296.7
			G. Mexico	26	high	11.6	22.1	31.4	60.2
				85	medium	53.6	65.8	-39.9	79.6
tactostoma macropus	0.36	0.97	G. Alaska	26	medium	277.5	252.3	2.3	18.3
				85	medium	1314.8	362.4	83.9	50.3
talismania bifurcata	0.42	0.85	West U.S.	26	medium	285.4	269.2	23.7	56.3
				85	medium	1091.9	510.2	456.0	313.9
tautogolabrus adspersus	0.26	0.86	NE U.S.	26	medium	110.8	103.4	-14.1	28.0
				85	medium	471.8	280.9	-68.2	29.5
tellina lutea	0.39	0.95	E. Bering S.	26	low	58.4	38.2	-34.0	57.1
				85	high	232.5	211.4	-96.7	6.0
telmessus cheiragonus	0.56	0.97	E. Bering S.	26	high	68.5	55.6	23.7	248.2
				85	high	266.2	230.4	-99.8	0.7
terebratalia transversa	0.25	0.77	G. Alaska	26	medium	247.9	239.6	62.3	71.6
				85	medium	793.9	220.4	81.5	131.2
tethyaster grandis	0.32	0.90	G. Mexico	26	high	34.0	25.4	29.6	30.5
				85	medium	83.5	72.6	67.4	70.4
thaleichthys pacificus	0.31	0.20	G. Alaska	26	low	314.8	234.7	19.3	21.8
				85	low	1010.6	160.0	64.9	35.1
theragra chalcogramma	0.55	0.27	E. Bering S.	26	low	68.5	55.5	-6.5	6.9
				85	low	176.8	90.5	-44.3	21.8
thrissacanthias penicillatus	0.53	0.90	West U.S.	26	medium	193.9	167.6	19.9	41.3
				85	high	621.3	333.7	112.6	113.3
torpedo californica	0.35	0.92	West U.S.	26	low	207.0	124.1	47.7	29.2
				85	low	858.4	323.1	219.6	132.6
trachinocephalus myops	0.47	0.91	SE U.S.	26	low	37.8	29.8	218.3	197.5
				85	low	82.3	46.4	3676.5	2174.9
			G. Mexico	26	medium	15.9	15.4	50.8	43.9
				85	low	45.7	25.5	317.8	130.3
trachinotus carolinus	0.39	0.99	SE U.S.	26	low	12.2	8.6	29.8	24.4
				85	medium	123.0	93.8	93.6	49.6
			G. Mexico	26	medium	27.2	27.9	0.3	9.2
				85	medium	117.5	77.1	-58.1	20.1
trachurus lathami	0.39	0.93	SE U.S.	26	medium	24.0	13.7	52.6	39.2
				85	low	101.2	50.7	317.2	192.7
			G. Mexico	26	medium	43.0	42.8	-9.6	13.4
				85	low	153.3	101.0	-69.0	19.9

Species	devPA	devBiom	Region	RCP	Uncert.	Shift	sd_shift	%Hab.	sd_hab
trachurus symmetricus	0.32	0.91	G. Alaska	26	low	376.7	282.6	23.8	19.0
				85	low	1462.9	281.7	102.0	62.4
trichiurus lepturus	0.41	0.94	SE U.S.	26	medium	11.5	9.3	13.1	16.5
				85	medium	80.1	76.6	113.0	82.4
			G. Mexico	26	low	32.1	25.3	22.9	23.0
				85	low	83.8	38.5	301.2	148.7
trichodon trichodon	0.31	0.86	E. Bering S.	26	medium	84.5	65.5	73.5	126.2
				85	low	279.6	139.1	161.8	320.0
trichopsetta ventralis	0.55	0.91	G. Mexico	26	medium	0.3	0.4	37.7	60.5
				85	medium	0.8	1.3	-3.6	100.6
triglops forficatus	0.26	0.93	E. Bering S.	26	low	85.8	59.5	-38.1	47.8
				85	low	255.9	73.5	-95.3	9.4
triglops macellus	0.12	0.12	G. Alaska	26	low	97.9	61.5	4.7	8.7
				85	low	377.3	77.2	10.3	19.1
triglops murrayi	0.35	0.77	E. Canada	26	low	95.4	77.0	30.3	66.6
				85	medium	438.8	273.7	16.7	90.5
triglops pingelii	0.25	0.95	E. Bering S.	26	low	65.1	46.2	-35.2	53.1
				85	low	161.8	51.0	-96.6	6.3
triglops scepticus	0.43	0.92	E. Bering S.	26	low	151.4	79.4	-50.4	47.0
				85	low	499.4	139.2	-98.6	3.3
trinectes maculatus	0.43	0.95	SE U.S.	26	low	23.1	17.2	2.9	5.4
				85	high	510.3	468.9	82.8	163.3
			G. Mexico	26	low	25.7	20.3	-22.8	13.9
				85	medium	67.0	49.4	-76.5	8.9
tritonia diomedea	0.29	0.09	E. Bering S.	26	medium	20.6	10.7	3.6	4.2
				85	medium	47.1	33.7	13.5	9.7
ulvaria subbifurcata	0.16	0.76	E. Canada	26	medium	88.6	67.9	-5.0	15.8
				85	medium	492.7	318.6	-45.3	33.2
upeneus parvus	0.50	0.97	SE U.S.	26	high	12.0	8.2	26.9	35.7
				85	low	108.5	73.0	18.9	70.5
			G. Mexico	26	low	26.4	23.3	-19.8	12.6
				85	medium	57.9	33.2	-43.3	13.2
urophycis chuss	0.41	0.78	E. Canada	26	high	330.1	254.7	-25.3	36.1
				85	high	745.1	401.6	-67.7	23.4
urophycis cirrata	0.50	0.92	G. Mexico	26	medium	44.9	45.3	3.5	16.5
				85	low	116.5	83.2	-27.6	26.9

Species	devPA	devBiom	Region	RCP	Uncert.	Shift	sd_shift	%Hab.	sd_hab
urophycis floridana	0.49	0.98	SE U.S.	26	low	26.3	21.4	44.9	29.4
				85	high	157.5	190.5	109.5	105.3
			G. Mexico	26	low	28.5	22.3	-16.1	10.3
				85	medium	77.0	41.9	-61.3	12.6
urophycis regia	0.46	0.78	SE U.S.	26	medium	129.2	114.1	30.1	31.7
				85	low	662.2	230.1	218.7	105.0
			G. Mexico	26	low	39.5	24.1	-8.5	6.4
				85	low	99.7	38.0	-28.4	7.3
urophycis tenuis	0.45	0.93	E. Canada	26	low	149.4	92.8	12.4	12.7
				85	low	563.6	238.7	7.7	29.6
urticina crassicornis	0.19	0.23	G. Alaska	26	low	167.5	112.2	37.1	30.0
				85	low	510.3	150.5	169.7	60.8
vampyroteuthis infernalis	0.44	0.95	G. Alaska	26	medium	362.6	400.9	16.6	35.5
				85	low	1630.7	561.1	230.8	104.8
volutopsius fragilis	0.26	0.89	E. Bering S.	26	medium	72.8	58.7	-29.3	59.4
				85	medium	195.6	81.1	-96.3	8.7
volutopsius middendorffii	0.29	0.98	E. Bering S.	26	low	51.8	35.5	-33.3	35.3
				85	low	180.6	93.4	-87.0	22.5
xiphopenaeus kroyeri	0.32	0.95	SE U.S.	26	medium	17.3	17.7	91.7	93.6
				85	medium	64.8	45.6	1296.2	1107.6
			G. Mexico	26	high	28.9	15.3	29.1	28.3
				85	medium	60.1	28.6	192.3	147.2
zalembius rosaceus	0.53	0.91	West U.S.	26	low	240.0	151.9	72.1	60.1
				85	low	964.3	307.1	358.3	194.8
zaniolepis frenata	0.54	0.98	West U.S.	26	low	272.6	202.3	52.7	40.8
				85	low	1216.3	392.6	270.2	124.3
zaniolepis latipinnis	0.52	0.94	West U.S.	26	low	153.0	117.1	58.6	49.3
				85	low	626.8	304.0	406.8	333.9
zaprora silenus	0.24	0.76	E. Bering S.	26	low	183.7	134.0	-3.2	19.3
				85	low	597.1	182.7	-51.7	55.7
zenopsis conchifera	0.40	0.71	E. Canada	26	high	89.0	108.9	1.7E+06	3.9E+06
				85	high	177.3	181.0	2.6E+09	7.0E+09
			G. Mexico	26	medium	0.6	0.6	-66.8	22.8
				85	high	1.5	2.0	-99.8	0.1
zoarces americanus	0.31	0.82	E. Canada	26	medium	77.8	45.0	-6.4	24.2
				85	high	263.4	92.2	-59.2	27.2
